# Traumatic Brain Injury Detection Using Electrophysiological Methods

**DOI:** 10.3389/fnhum.2015.00011

**Published:** 2015-02-04

**Authors:** Paul E. Rapp, David O. Keyser, Alfonso Albano, Rene Hernandez, Douglas B. Gibson, Robert A. Zambon, W. David Hairston, John D. Hughes, Andrew Krystal, Andrew S. Nichols

**Affiliations:** ^1^Uniformed Services University of the Health Sciences School of Medicine, Bethesda, MD, USA; ^2^Bryn Mawr College, Bryn Mawr, PA, USA; ^3^US Navy Bureau of Medicine and Surgery, Frederick, MD, USA; ^4^U.S. Army Research Institute, Fort Belvoir, VA, USA; ^5^Booz Allen Hamilton Inc., McLean, VA, USA; ^6^U. S. Army Research Laboratory, Aberdeen Proving Ground, Aberdeen, MD, USA; ^7^Naval Medical Research Center, Silver Spring, MD, USA; ^8^Duke University, Durham, NC, USA

**Keywords:** event-related potentials, EEG, traumatic brain injury, qEEG, non-linear dynamical analysis

## Abstract

Measuring neuronal activity with electrophysiological methods may be useful in detecting neurological dysfunctions, such as mild traumatic brain injury (mTBI). This approach may be particularly valuable for rapid detection in at-risk populations including military service members and athletes. Electrophysiological methods, such as quantitative electroencephalography (qEEG) and recording event-related potentials (ERPs) may be promising; however, the field is nascent and significant controversy exists on the efficacy and accuracy of the approaches as diagnostic tools. For example, the specific measures derived from an electroencephalogram (EEG) that are most suitable as markers of dysfunction have not been clearly established. A study was conducted to summarize and evaluate the statistical rigor of evidence on the overall utility of qEEG as an mTBI detection tool. The analysis evaluated qEEG measures/parameters that may be most suitable as fieldable diagnostic tools, identified other types of EEG measures and analysis methods of promise, recommended specific measures and analysis methods for further development as mTBI detection tools, identified research gaps in the field, and recommended future research and development thrust areas. The qEEG study group formed the following conclusions: (1) Individual qEEG measures provide limited diagnostic utility for mTBI. However, many measures can be important features of qEEG discriminant functions, which do show significant promise as mTBI detection tools. (2) ERPs offer utility in mTBI detection. In fact, evidence indicates that ERPs can identify abnormalities in cases where EEGs alone are non-disclosing. (3) The standard mathematical procedures used in the characterization of mTBI EEGs should be expanded to incorporate newer methods of analysis including non-linear dynamical analysis, complexity measures, analysis of causal interactions, graph theory, and information dynamics. (4) Reports of high specificity in qEEG evaluations of TBI must be interpreted with care. High specificities have been reported in carefully constructed clinical studies in which healthy controls were compared against a carefully selected TBI population. The published literature indicates, however, that similar abnormalities in qEEG measures are observed in other neuropsychiatric disorders. While it may be possible to distinguish a clinical patient from a healthy control participant with this technology, these measures are unlikely to discriminate between, for example, major depressive disorder, bipolar disorder, or TBI. The specificities observed in these clinical studies may well be lost in real world clinical practice. (5) The absence of specificity does not preclude clinical utility. The possibility of use as a longitudinal measure of treatment response remains. However, efficacy as a longitudinal clinical measure does require acceptable test–retest reliability. To date, very few test–retest reliability studies have been published with qEEG data obtained from TBI patients or from healthy controls. This is a particular concern because high variability is a known characteristic of the injured central nervous system.

## Introduction

Mild TBI is caused by initial physical trauma that shears or compresses brain tissue. The initial trauma can lead to a cascade of delayed neurodegenerative events that may include diffuse axonal injury, activation of excitotoxic inflammatory cascades, and transneuronal degeneration. While initial damage in mild traumatic brain injury (mTBI) may be minimal or occult, the chronic neurodegenerative effects can persist for weeks or months post-injury and lead to significant cognitive, sensory, and psychiatric dysfunctions (DeKosky et al., 2010). Over 75% of the 266,000 brain injuries reported during U.S. military operations from 2000 to 2012 were classified as mTBI, thus underscoring mTBI as a major health issue in the U.S. military (Hoge et al., 2008). It is anticipated that rapid and accurate mTBI detection would improve prognoses and minimize impacts to military operations; however, a technological gap exists, especially in field-based settings. To address this critical need, the Department of Defense is actively seeking new technologies capable of rapid, accurate, non-invasive, and field-capable detection of mTBI (Rigg and Mooney, 2011).

One promising avenue is measurement of brain electrical activity with quantitative electroencephalography (qEEG), in which detection of altered patterning may indicate concussion. Since qEEG is a nascent technology for mTBI detection, and significant controversy exists in the field, a comprehensive evaluation of technology/measure efficacy as a detection tool is warranted. Critically surveying the state-of-the-science also provides an opportunity to establish recommendations on specific qEEG measures or signal processing technologies of promise, thus driving advanced development decision-making.

### Background on qEEG

The electroencephalogram (EEG) records the electrical potential difference between brain electrical activities recorded between two electrodes (a single EEG channel). Multiple scalp electrodes (generally >20) are connected in various patterns called montages, resulting in a series of channels of EEG activity. Often the scalp electrodes are compared to one or more neutral reference electrodes. Detection of a scalp electrical potential requires the synchronous activity of millions of neurons over at least 10 cm^2^ of cortex. The EEG detects the synchronous occurrence of dendritic synaptic potentials (excitatory and inhibitory postsynaptic potentials) principally on the apical dendrites of cortical pyramidal neurons. Therefore, the EEG records the average membrane potential of these apical dendrites, which tends to oscillate. The physiological basis of these oscillations is a manifestation of both intrinsic properties of neurons (ionic conductances) and, importantly, network interactions (connectivity). Intrinsic resonant properties permit and promote frequency range-specific oscillations based on network activity [see Berridge and Rapp (1979)]. Different frequency oscillations are governed principally by cortico-cortical connections and thalamocortical connections to varying degrees depending on the specific oscillation. EEG oscillations exist in a broad range of frequencies from well below one Hertz to several hundred Hertz. The physiology is best understood for several “classic” frequency bands including delta (0.5–4 Hz), theta (4–7 Hz), alpha (8–13 Hz), beta (14–30 Hz), and gamma (30–100 Hz). These frequency ranges are not arbitrarily divided [see Penttonen and Buzsáki (2003)], but represent different oscillatory phenomena with unique underlying physiological mechanisms, cortical topographies, and functions, of which many are still being delineated. Different combinations of frequency bands in different quantities comprise different states of the brain (e.g., attentive wakefulness, drowsiness, various stages of sleep).

The “clinical” evaluation of the EEG typically involves a visual inspection of brain electrical activity across a range of brain states and the assessment of the topography and “quantity” of state-appropriate oscillatory activity, as well as examination for the presence of pathological potentials. For the detection of epileptiform activity, the human eye actually outperforms computerized waveform analysis despite decades of attempts to automate EEG interpretation (Harner, 2010). However, when it comes to the assessment of the topography and quantity of oscillatory activity, and what constitutes a normal or abnormal distribution of such activity, visual inspection fails considerably. Very poor inter-rater reliability is common for visual inspections of oscillatory activity, even in determining what constitutes normal vs. abnormal. Additionally, visual inspection can shed no light whatsoever on the nature of interregional interactions, both locally and globally, which constitute the basis for cognition and consciousness (Tononi et al., 1994). This requires the use of computerized algorithmic methodologies called qEEG, which we will use as a broad encompassing term for all such analyses. For example, to obtain a quantitative assessment of the amount of oscillatory activity at any and all frequencies in a particular brain region, qEEG frequency analysis transforms the original EEG data over a period of time into a representation of its frequency content, generating a continuous EEG “power spectrum.” Ultimately, however, using methodologies that require averaging of data over relatively long periods of time will be insensitive to the rich dynamics of the cortex, which operates on a multitude of timescales down to the order of milliseconds (see below).

### EEG in mTBI

Pathologically, mTBI is a complex process, resulting in neuronal dysfunction that may be manifested in EEG changes apparent even to visual inspection for a duration ranging from hours to up to a few weeks post-injury. Beyond the acute and subacute stages, the structural pathology of mTBI is characterized by diffuse axonal injury involving the white matter as well as simplification of the dendritic architecture of neurons in cortical gray matter, with a relative absence of neural loss, leading to varying and often subtle degrees of cortical atrophy or thinning (Bigler and Maxwell, 2011). Such effects below a certain threshold of severity will not manifest on standard magnetic resonance imaging (MRI) at any stage. Newer MRI methods, such as diffusion tensor imaging, may be capable of detecting these more subtle structural disturbances, but these imaging techniques are not common in practice and are still in early stages of use.

Electroencephalogram, however, may be sensitive to detecting the physiological effects of these processes. The simplified dendritic system of neurons may result in a decrease in power of fast frequencies. Even subtle relative deafferentation from the thalamus or ascending neuromodulator systems (i.e., cholinergic, noradrenergic, dopaminergic) due to axonal injury will increase the power of slow frequencies, due to an alteration of firing properties of thalamic and cortical neurons (resulting in a net increase in theta frequency activity), and due to the release of intrinsically generated network oscillatory activity normally suppressed by cholinergic afferents (from the basal forebrain) and glutamatergic afferents (from the thalamus). Additionally, and importantly, axonal injury will reduce the effectiveness of synchronization of distributed cell assemblies located throughout the cerebral cortex, which are essential for cognition. The physiological basis of the EEG, as described above, makes qEEG methodologies potentially well-suited to detect these physiological alterations in the “free-running” EEG oscillatory activity. Additionally, these pathological changes can alter the timing and amplitude of slower non-oscillatory electrical potentials, which are caused by the phase resetting of these oscillatory rhythms during the performance of cognitive tasks, the so-called event-related potentials (ERPs). Thus, the assessment of ERPs is another potentially valuable tool in the qEEG repertoire for the study of altered cortical physiology in the setting of mTBI.

What is the function of EEG oscillations, and how can a quantitative analysis of EEG activity provide insight into impaired brain function in TBI? For several decades from 1960s to 1990s, the prevailing view was that EEG activity/oscillations were epiphenomenal and unrelated to moment-to-moment cerebral cortical function, but simply reflected the general state of the brain (e.g., awake and alert, drowsy, asleep). The tide has turned dramatically and it is now understood that moment-to-moment brain function is directly linked to EEG oscillations. Cognitive processes are a function of the rapid formation and subsequent dissolution of distributed cell communication assembles on the order of several hundred milliseconds in duration (Breakspear et al., 2004), and these assemblies form transiently based on a functional (as opposed to structural) connectivity patterns established by the synchronization patterns of EEG oscillations. This synchronization allows effective communication among the members of the transient assembly (Fries, 2005). True synchronization of neuronal activities only occurs within a few millimeters. A better term may be “polychronization” (Izhikevich, 2006), as most functionally connected neurons are not truly synchronized, but that term will suffice for our purposes. Fast frequencies (especially gamma) are involved in the synchronization of relatively local cell assemblies in the processing of specific cortical representations and slower frequencies synchronize highly distributed cell assemblies that span many centimeters of cortex and may involve both hemispheres. Theta and delta frequency activity also support the synchronization of faster frequencies among multiple localized assemblies via the phenomenon of “cross-frequency coupling” (Canolty and Knight, 2010). Computerized signal analysis of EEG data has evolved significantly to address the complex interregional interactions of brain connectivity, including the causal relationships among interacting regions of brain during a cognitive act and how these relationships may be deranged. For example, in brain injury one could demonstrate altered causal relationships in which control of action is more reactive or stimulus-based, such that cortical sensory networks predominantly influence frontal-executive networks instead of vice versa. A so-called “small-world” connectivity analysis can determine whether or not the cerebral cortices in a population of patients is performing with a functional connectivity pattern that optimizes the ratio of local to long-range functional connectivity. This ratio has shown to be altered in TBI (Cao and Slobounov, 2010; Tsirka et al., 2011). Overall, recent developments in the analysis of EEG activity to characterize brain function hold tremendous promise for our ability to understand the physiology of cognitive processes and their pathophysiological derangement in neurological disorders such as traumatic brain injury.

The effects of TBI can be significant and long-lasting. Fazel et al. (2014) compared mortality rates 6 months or more after injury against a control population. They found that TBI is associated with substantially elevated risks of premature mortality, particularly from suicide, injuries, and assaults. Similarly, TBI is a significant risk factor for neuropsychiatric disorders (Rapp et al., 2013a). These results argue against the view that TBI, even mTBI, typically resolves without lasting consequences. In addition, these results establish the need to identify individuals at-risk of delayed onset neuropsychiatric disorders following brain injury. Quantitative measures of altered brain electrical behavior may provide quantitative prodromes of neuropsychiatric disorders. This possibility is encouraged by noting that changes in brain electrical behavior following TBI can be persistent. Slobounov et al. (2012) found that 85% of the mTBI patients who presented significant EEG alterations in the immediate post-injury period still presented altered EEGs up to 12 months post-injury. Segalowitz et al. (2001) found that ERPs were altered in mTBI patients in a patient group that was on average 6.4 years post-injury. De Beaumont et al. (2009) examined healthy former athletes in late adulthood (mean age 61 years) who had sustained their last sports related concussion in early adulthood (mean age at time of last injury 26 years). These participants were compared against healthy former athletes who did not have a history of concussion (average age 69 years). Participants with a history of concussion had significantly different ERPs. These results must be considered with care; however, as they represent a problem that is common to studies in this area: in almost all cases, pre-injury data are not available. It is possible, for example, that central nervous system (CNS) abnormalities in the athletes in the de Beaumont study were present prior to injury and were themselves a factor leading to injury. Nonetheless, these results and other studies summarized in this report suggest that measurement of brain electrical behavior may be a valuable complement to other assessment procedures.

### Comparative utility of qEEG for mTBI detection

Aside from the direct connection between qEEG and the physiological responses described above, EEG supplements conventional medical approaches to imaging the structure and function of the brain. EEG as a neuroimaging modality holds several advantages over more conventional medical approaches. Computed tomography (CT) and MRI are current the “gold standards” for imaging assessment of neurophysiological trauma. These techniques provide excellent spatial resolution for easily identifying lesions; however, significant limitations of CT and MRI reduce the practical utility for mTBI detection. For instance, both approaches require very large and expensive equipment, special facilities for their use, and dedicated technicians for operation. CT uses small doses of radiation, which may carry potential risks for long-term side effects if patients are scanned often. MRI uses an extremely strong magnetic field (>1 T), necessitating careful operating procedures. Perhaps most limiting for MRI is the contraindication of medical devices, implants, and any foreign ferrous metal objects in the patient’s body. Since service members may have metal fragments lodged in their bodies from the same injury event, this limitation is particularly difficult to reconcile for military use. In contrast to CT and MRI, equipment used for EEG is substantially more portable, less expensive, requires no special facilities, and, in most cases, can be applied by personnel with very minimal training. All of these characteristics contribute to a substantially higher level of fieldability and a broader operational effectiveness beyond dedicated medical centers. The fieldability of qEEG also facilitates neuroscience research outside of laboratory environments, for which more accurate “real world” data can be captured and compared to findings in the laboratory, thus driving advancements in neurotechnology applications (McDowell et al., 2013).

The data derived from EEG are also fundamentally distinct as compared to CT and MRI data. In particular, while CT and MRI have excellent spatial resolution, the resulting images (which take several minutes to acquire) are temporally static, and thus provide no direct measurement of functional, ongoing brain activity. Even the best current methods of “functional MRI” are limited to multiple seconds for the acquisition of whole-brain images. In contrast, EEG is extremely high-resolution in the time domain (EEG can be sub-millisecond) and, as described above, is a direct measurement of neuronal activity. This allows a great number of analyses (as described above and in the following sections), which capitalize on direct responses to stimuli, cognitive responses, inter-relatedness of continuous signals, and the oscillation of both discrete and cross-regional networks of brain areas.

## Literature Index of qEEG Studies Relevant to mTBI

### Literature search methodology

A literature search was conducted in peer-reviewed primary sources from PubMed and the “gray” literature (i.e., documents not peer-reviewed) from the defense technical information center (DTIC) technical report database. Keyword searches were chosen to identify studies that specifically measured qEEG in head injured groups. See Supplementary Material for keyword search terms and record returns. Studies using established statistical approaches to demonstrate differential qEEG between injured and control groups were considered particularly important. Statistical methods of high value included effect size statistics such as Cohen’s *d*, odds ratio, and area under receiver operating characteristic curve. In addition, studies exploring test–retest reliability were flagged. To efficiently filter the large number of initial hits and maintain a pertinent collection for TWG review, the following study characteristics resulted in *exclusion* from the primary qEEG literature index:

1. Studies that used severe TBI or comatose patients,
2. Studies exploring pharmacologic or biologic agent efficacy,
3. Rehabilitation or therapeutic studies (those not diagnostic in nature),
4. Studies using animal models of neurological injury, and
5. Studies exploring the use of qEEG in dysfunctions besides head injury (e.g., neurodegenerative diseases, psychological dysfunctions).

The primary qEEG literature index was further down selected to focus on studies that provide statistical evidence for *qEEG measures* as detection tools for mTBI. Therefore, the following study characteristics resulted in removal from the qEEG-refined literature index:

1. Retrospective studies (i.e., symptom catalogs)
2. Studies without direct statistical comparisons.

### Literature search results

Using the methodology described above, the primary qEEG literature index consisted of 40 studies (Supplementary Material). Twenty-five of these studies were further down selected as providing direct statistical evidence related to qEEG measure discriminatory ability. The refined literature index was subsequently used by the TWG to evaluate major qEEG measure types, power spectra, connectivity measures, and the use of discriminant functions. Each analysis of the measure types and the use of discriminant functions includes a general description of the approach, the potential utility for detection of mTBI, an evaluation of the statistical evidence to discriminate between injured and non-injured persons, research gaps and future directions to improve upon the evidentiary data, and conclusions on the overall prospects for use as an mTBI detection method. Section “Advanced Signal Processing Technologies” extends the scope of the original literature search to introduce important approaches with high perceived value (based on the opinion of this TWG) as mTBI diagnostic tools, but with low evidentiary support to date.

## Measures Represented in the Down Selected qEEG Literature Index

Examination of the down selected literature index revealed two main types of qEEG analysis investigated for mTBI detection efficacy: spectral analysis (see Table 1) and functional connectivity analysis. Each type of analysis is introduced below, followed by critical evaluation of the literature-based evidence and recommendations for future research.

**Table 1:** **Studies using spectral analysis**.

General study information (spectral analysis)	Study design				Results	
	mTBI group (*n*)	Control group (*n*)	qEEG recording condition	Criteria for TBI patient	Measures	Statistical test/comparison/significance
Chen et al. (2006a) “Electroencephalogram and evoked potential parameters examined in Chinese mild head injury patients for forensic medicine”	60	30	Eyes closed, resting, wakeful	Glasgow coma scores (13–15)	Frequency band average power	Unpaired *t*-test
						1. α1 (*p* < 0.001)
						2. θ, α2, β1, β2 (all *p* > 0.05) (NS)
					Frequency band power ratios	Unpaired *t*-test
						1. θ/α1 and θ/α2 (both *p* < 0.05)
						2. α1/α2 (*p* < 0.01)
					Frequency band power ratios and average power	Paired *t*-test Initial vs. 3 month retest (*p* > 0.05 for all comparisons) (NS)
*Notes*: this study supports qEEG as a more sensitive method for detecting mTBI compared to evoked potential recording (auditory and flash). This conclusion is somewhat contradictory to other studies in the field

Coutin-Churchman et al. (2003) “Quantitative spectral analysis of EEG in psychiatry revisited: drawing signs out of numbers in a clinical setting” *Notes*: qEEG was reported to bin normal subjects and psychiatric patients with good specificity and sensitivity. However, this study does not find any clear association between particular qEEG measures and specific disorders. In addition, the patient group mostly consisted of individuals with mood and psychological disorders, not cognitive impairments due to injury	4 (Post-traumatic headache 336 With other neurological disorders	67	Eyes closed, resting, wakeful	Post-traumatic headache diagnosis	Power spectra (all bands), converted to *Z*-score (all disorders combined)	*Z*-score abnormality defined as *Z*-score >3 or <−3
						1. Sensitivity (0.838)
						2. Specificity (0.9104)
						3. Positive predictive value (0.979)
						4. Negative predictive value (0.526)
					Absolute power and power asymmetry (all disorders combined)	Various tests
						Overall correspondence between qEEG abnormality pattern and clinical diagnosis (χ^2^ = 315.8, Cramer’s *V* = 0.305, Contingency coefficient = 0.694; *p* = 0.00001)
					Beta activity increase (all disorders combined)	Pearson’s correlation
						Medication use in all patients (Pearson χ^2^ = 7.865, df = 1, *p* = 0.005)
					Slow band decrease (all disorders combined)	Pearson’s correlation
						Medication use in all patients (χ^2^ = 2.963, df = 1, *p* = 0.085) (NS)

Gosselin et al. (2009) “Sleep following sport-related concussions” *Notes*: while this report did not find differences in sleep architecture (frequency activities) between concussed and control athletes, concussed athletes showed significant increased delta and reduced alpha activities in the waking qEEG analysis. This study also demonstrates effective combinatorial technology use (fMRI and ERP)	10	11	Eyes closed, resting, wakeful Eyes closed, asleep Frontal region (Fz, F3, F4), central region (CZ, C3, C4), parietal region (Pz, P3, P4), occipital region (O1 and O2), temporal region (T7, T8, P7, P8)	Glasgow coma scores (13–15)	Relative spectral power (all frequencies) (eyes closed asleep)	Two-way ANOVA (no significance for any region)
					Relative delta power (eyes closed, wakefulness)	Two-way ANOVA (*F*_1, 14_ = 12.7, *p* < 0.01) (all regions)
					Relative alpha power (eyes closed, wakefulness)	Two-way ANOVA (*F*_1, 14_ = 8.8, *p* < 0.05) (all regions)
					Slow to fast frequencies ratio (eyes closed, wakefulness)	Two-way ANOVA (*F*_1, 14_ = 11.5, *p* < 0.01) (all regions)

Haglund and Persson (1990)	47	50	Eyes closed, resting, wakeful	High number of boxing matches (>25)	Average power spectrum (grand averages from each frequency)	Chi square (no significant differences between all groups)
“Does Swedish amateur boxing lead to chronic brain damage? A retrospective clinical neurophysiological study”	22 High-match boxers	25 Soccer players	
*Notes*: study reports no differences in qEEG measures between patient groups, but the effort was exploratory and used grand averages for each frequency band. Importantly, inter-rater reliability was reported to be sufficient	
	25 Low-match boxers	25 Track and field players	

Korn et al. (2005) “Focal cortical dysfunction and blood-brain barrier disruption in patients with post-concussion syndrome” *Notes*: the main purpose of this study was to correlate abnormal EEG, brain imaging, and blood–brain barrier disruptions, especially as related to localization. Arciniegas’ (2011) review provides critical perspective on this study (classification of some experimental group subjects as having mTBI, but CT scans suggest a more severe form of TBI)	17	17	N/A	Glasgow coma scale >12, post-concussion syndrome (PCS) diagnosis 1 month to 7 years post-injury	Delta frequency power Alpha frequency power	Student’s *t-*test Increased delta (*p* < 0.01) Student’s *t-*test Lower alpha 1 (*p* < 0.05) Lower alpha 2 (*p* < 0.05)

Montgomery et al. (1991) “The psychobiology of minor head injury” *Notes*: the theta band was the only abnormal frequency in the qEEG analysis. However, no normative sampling or pre-injury measures, or control group measures were used. The comparison was only between immediate post-injury (approximately 24 h) and 6 weeks later. So, reduction in theta power may be considered persistent	26	None	Eyes closed, resting, wakeful	Head injury requiring overnight hospital stay; post-traumatic amnesia >12 h	Mean theta power (comparison between day 0 and 6 weeks)	Two-tailed paired *t*-tests
						1. Right temporal, T4–T6 (*p* < 0.025)
						2. Right parietal, P4–O2 (*p* < 0.01)
						3. Left temporal, T3–T5 (*p* < 0.01)
						4. Left parietal, P3–O1 (*p* < 0.01)
					Mean alpha power (comparison between day 0 and 6 weeks)	Two-tailed paired *t*-tests
						No significance at any region
					Mean delta power (comparison between day 0 and 6 weeks)	Two-tailed paired *t*-tests
						No significance at any region

Slobounov et al. (2012) “Residual brain dysfunction observed 1 year post-mild traumatic brain injury: combined EEG and balance study” *Notes*: this study suggests qEEG and balance analysis as good prognostic tools; efficacy as a diagnostic tool was not explored	49	None (used baseline testing from mTBI group pre-injury)	Eyes closed, resting, wakeful Eyes open, resting, wakeful Eyes closed, standing, wakeful Eyes open, standing, wakeful	Grade 1 mTBI (Cantu Dad Driven Revised Concussion Grading Guideline, 2006)	Alpha power suppression (from resting to standing posture) increase	*t*-Test (baseline vs. day 7 post-injury) 1. Occipital region of interest: *F* = 11.77 (1,48), *p* < 0.01 2. Parietal region of interest: *F* = 8.2 (1,48), *p* = 0.038

Tebano et al. (1988) “EEG spectral analysis after minor head injury in man” *Notes*: this report suggests differences in power band frequencies occur between healthy and patients with mTBI. This is an early study and may be considered exploratory	9 (No loss of consciousness) 9 (Reported loss of consciousness)	9	Eyes closed, resting, wakeful (baseline recording) Eyes open, resting, wakeful (recording)	Reported loss of consciousness after injury	Alpha I band power (higher in injured group)	Mann–Whitney test *U* = 28, *p* < 0.01
					Alpha II band power (reduced in injured group)	Mann–Whitney test
						*U* = 47, *p* < 0.05
					Mean frequency of total alpha band power (reduced in injured group)	Mann–Whitney test
						*U* = 46, *p* < 0.05
					Frequency band total power (if significant, reduced in injured group)	Mann–Whitney test
						1. Alpha (alpha I and II), (NS)
						2. Delta, *U* = 45, *p* < 0.05
						3. Beta II, *U* = 41, *p* < 0.025

Thatcher et al. (2001) “Estimation of the EEG power spectrum using MRI T_2_ relaxation time in traumatic brain injury” *Notes*: Nuwer et al. (2005) criticizes this study due to lack of age-matching and broad spread of TBI severity	18 (Mild-severe TBI)	11	Eyes closed, resting, wakeful	Chronic TBI diagnosis	Correlation between relative EEG frequency power and T2 relaxation time (MRI scan) (decreased alpha and beta; increased delta and theta with MRI T2 abnormalities)	ANOVA
						1. Delta (*t* = 8.876, *p* < 0.0001)
						2. Theta (*t* = 8.529, *p* < 0.0001)
						3. Alpha (*t* = −9.276, *p* < 0.0001)
						4. Beta (*t* = 2.421, *p* < 0.016)

Thatcher et al. (1991)	162	None	N/A	Closed head injury and admittance to Neurotrauma hospital center	EEG relative power	Prediction accuracy (extreme outcome scores) (1 year) 67.1%
“Comprehensive Predictions of Outcome in Closed Head-Injured Patients”	
*Notes*: a gradient of prognostic strength of diagnostic measures was EEG phase > EEG coherence > GCS-*T* > CT scan > EEG relative power	

Thornton (2003) “The electrophysiological effects of a brain injury on auditory memory functioning. The QEEG correlates of impaired memory” *Notes*: twenty-seven patients of the mTBI group were on various medications. Age was statistically different between groups (mTBI group was slightly older)	85	56	Eyes closed, resting, wakeful, during auditory memory task Eyes open, resting, wakeful, during auditory memory task	Loss of consciousness <20 min	Power band frequency measures for alpha, beta, theta, and delta (includes absolute/relative magnitude, peak amplitude, peak frequency, and symmetry)	*t*-Test 1. Increased beta1 relative power (*p* < 0.05)

Tomkins et al. (2011) “Blood–brain barrier breakdown following traumatic brain injury: a possible role in posttraumatic epilepsy” *Notes*: study focuses mostly on blood–brain barrier localization with qEEG abnormalities	37 (19 With post-traumatic epilepsy, PTE)	13	Eyes closed, wakeful	Glasgow coma scale >13	Delta power (increase)	Mann–Whitney *U* test Injured vs. controls (*p* = 0.002) (equivalent among PTE and non-PTE mTBI patients)
					Alpha power (decrease)	Mann–Whitney *U* test
						Injured vs. controls (*p* = 0.005) (only seen in PTE group, *p* = 0.01)
					Theta power (increase)	Mann–Whitney *U* test
						PTE group of mTBI patients vs. controls (*p* = 0.04)

von Bierbrauer et al. (1992) “Computer assisted vs. visual EEG-analysis in patients with minor head injury (a follow-up examination)” *Notes*: this study also provides evidence for qEEG as a relevant technology for patient diagnosis and analysis as compared to standard EEG. Original article is in German; analysis adapted from review article (Nuwer et al., 2005)	31	None	N/A	N/A	Median posterior alpha frequency (increase over time)	*t*-Test
						1. 24 h vs. 1 week (NS)
						2. 24 h vs. 3 weeks (*p* < 0.01)
						3. 24 h vs. 2 months (*p* < 0.01)
					Theta/alpha ratio (decrease over time)	*t*-Test
						1. 24 h vs. 1 week (NS)
						2. 24 h vs. 3 weeks (*p* < 0.05)
						3. 24 h vs. 2 months (*p* < 0.01)

Watson (1995) “The post-concussional state: neurophysiological aspects” *Notes*: study suggests combined use of qEEG power spectral analysis and brainstem audio evoked potential (BAEP) I–V latency analysis could provide utility to diagnose mTBI symptoms (both organic and psychological)	25	None	Eyes closed, resting, wakeful	Men; aged 14–30; uncomplicated head injury with post-traumatic amnesia <12 h	Alpha/theta ratio (at day 0, day 10, and 6 weeks post-injury)	Paired two-tailed *t*-test (day 0 vs. day 10 post-injury)
						1. Right temporal (T4–T6), *p* < 0.006
						2. Right parieto-occiptal (P4–O2), *p* < 0.02 (day 10 vs. 6 weeks) – not significant for any region

Williams et al. (2008) “Polysomnographic and quantitative EEG analysis of subjects with long-term insomnia complaints associated with mild traumatic brain injury” *Notes*: this study shows limited ability of qEEG power spectra analysis to differentiate among mTBI patients suffering from sleep disorders and control groups	9	9	Eyes closed, resting, asleep	Glasgow coma scale 13–15; loss of consciousness <20 min; hospitalization <48 h; reported sleep dysfunction	Beta 2 power (decrease in injured patients)	Two-tailed *t*-tests with Welch’s corrections (Wilcoxon rank sum test also used if outliers were apparent)
						1. Beta 2 (*F*(1, 16) = 8.9, *p* = 0.008)
					Variability in power (greater variability in mTBI patients)	Two-tailed *t*-tests with Welch’s corrections (Wilcoxon rank sum test also used if outliers were apparent)
						1. Sigma (*F*_1,16_ = 10.5, *p* = 0.005)
						2. Theta (*F*_1,16_ = 6.8, *p* = 0.019)
						3. Delta (*F*_1,16_ = 9.2, *p* = 0.008)

### Spectral analysis

#### Definition (spectral analysis)

Spectral analysis is a common form of EEG interpretation in which the distribution of signal is evaluated over various EEG frequencies. Spectral analysis is usually limited to a narrow frequency band from 0.1 to 100 Hz, which is further subdivided into several sub-bands (e.g., delta, theta, alpha, beta, and gamma bands). The boundaries of the sub-bands can differ slightly among various researchers, resulting in potential variation of results across studies. A typical sub-band range, used by Prichep et al. (2012a,b), is as follows: delta (1.5–2.5 Hz), theta (3.5–7.5 Hz), alpha (7.5–12.5 Hz), alpha1 (7.5–10.0 Hz), alpha2 (10.0–12.5 Hz), beta1 (12.5–25.0 Hz), beta2 (25.0–35.0 Hz), gamma (35.0–50.0 Hz). The spectral power for each band is obtained either by (1) calculating the power spectrum of the total signal recorded at an electrode site and then summing the contributions of the frequencies included in the band, or (2) subjecting the total signal to an appropriate passband filter and then calculating the power spectrum of the filtered signal. The resulting band power is typically averaged over electrode sites and reported either as “absolute power” or as “relative power,” which is the ratio of the band power to the total power over all bands. Other common parameters of spectral analysis include median frequency and spectral edge frequency (Dressler et al., 2004).

#### Utility for mTBI detection (spectral analysis)

A key feature of spectral analysis that allows for strong utility as an mTBI detection tool is that spectral data can be obtained from as few as two electrodes. Therefore, fielded devices do not require the costs or burden of large montages of electrodes. This feature reduces equipment burden, administration time, and cost. Simplified devices also allow for faster training and simplified analysis.

#### Evidence of efficacy (spectral analysis)

Of the 25 studies comprising the down selected qEEG literature index, 15 studies used spectral analysis as a measure to compare injured and control groups. Most of the studies report statistically significant alteration in at least one frequency band in mTBI patients compared to control groups. To visualize the overall trends in results, Table 2 summarizes the reported changes to spectral power (either absolute or relative power) across frequency sub-bands from relevant studies. The table also displays the recording montage and statistical significance from each finding. Measurements were made under eyes closed, no task, but awake conditions, with few exceptions [e.g., Tebano et al., 1988 – eyes open; Gosselin et al. (2009) – asleep condition; Thornton (2003) – EEG recorded during memory task]. While results are somewhat varied, generalizations can be inferred. For instance, mTBI injury is often associated with a decrease in alpha power and an increase in delta, beta, and theta bands. We note this evidence is not based entirely on well-designed studies with large sample groups and strong statistical significance. As an example, Thornton (2003) reported increased beta activity in the injured group, but used 27 mTBI patients that were on some type of medication. Furthermore, the age difference between injured and control groups was statistically significant. These inconsistencies could potentially skew EEG recordings. Korn et al. (2005), which reported decreased alpha and increased delta power in the injured group, has been criticized due to the potential misclassification of moderate-severe TBI patients as mTBI patients (Arciniegas, 2011). The absence of a uniformly applied criterion for defining mild TBI is a significant complicating factor. Tebano et al. (1988), inconsistent with several other studies, reported results based on very small sample sizes and recorded brain activity with a single bipolar derivation (O1–T5) using an eyes open paradigm. Other inconsistencies in the findings may be attributed to the various montages and derivations used during data acquisition sessions (see Table 2 for details). Overall, differential spectral power in injured patients is strongly suggested in the literature, but specific findings need to be confirmed based on robust studies that use similar recording conditions.

**Table 2:** **Summary of changes in spectral power associated with mTBI^a^**.

Frequency range	Decrease in spectral power	Increase in spectral power	Unchanged spectral power
Delta (1.5–3.5 Hz)	Tebano et al. (1988) (*p* < 0.01; O1–T5, relative power)	Korn et al. (2005) (*p* < 0.01; 128 channels, relative power); Gosselin et al. (2009) (*p* < 0.01, asleep, relative power); Tomkins et al. (2011) (*p* = 0.002, 23 channels)	

Theta (3.5–7.5 Hz)		Tomkins et al. (2011) (*p* = 0.04, 23 channels); Montgomery et al. (1991) (*p* < 0.025, T4–T6; *p* < 0.01, P4–O2; *p* < 0.01, T3–T5; *p* < 0.01, P3–O1)^b^	Tebano et al. (1988) (O1–T5, relative power); Chen et al. (2006a) (16 channels, specific locations unknown)

Alpha1 (7.5–10 Hz)	Korn et al. (2005) (*p* < 0.05; 128 channels, relative power)	Tebano et al. (1988) (*p* < 0.01; O1–T5); Chen et al. (2006a) (*p* < 0.01; 16 channels, specific locations unknown)	

Alpha2 (10.0–12.5 Hz)	Korn et al. (2005) (*p* < 0.05; 128 channels, relative power); Tebano et al. (1988) (*p* < 0.05; O1–T5, relative power)		Chen et al. (2006a) (16 channels, specific locations unknown)

Alpha (7.5–12.5 Hz)	Gosselin et al. (2009) (*p* < 0.05, asleep, relative power); Tomkins et al. (2011) (*p* = 0.005,only in post-traumatic epilepsy mTBI subgroup)		Tebano et al. (1988) (O1–T5, relative power)

Beta (12.5–25 Hz)			Tebano et al. (1988) (O1–T5, relative power)

Beta1 (13–32 Hz)		Thornton (2003) (*p* < 0.02, auditory memory task)	Chen et al. (2006a) (16 channels, specific locations unknown)

Beta2 (25–35 Hz)	Tebano et al. (1988) (*p* < 0.025; O1–T5, relative power); Williams et al. (2008) (*p* = 0.008, 10/20 config, asleep)	Thornton (2003) (*p* < 0.05, auditory memory task)	Chen et al. (2006a) (16 channels, specific locations unknown)

Gamma (35–50 Hz)			

Theta/alpha		Watson (1995) (*p* < 0.006, T4–T6; *p* < 0.02, P4–O2, P3–O1)^c^	

Theta/alpha1	Chen et al. (2006a) (*p* < 0.05; 16 channels, specific locations unknown)		

Theta/alpha2		Chen et al. (2006a) (*p* < 0.05; 16 channels, specific locations unknown)	

Alpha1/alpha2		Chen et al. (2006a) (*p* < 0.01; 16 channels, specific locations unknown)	

*^a^EEGs were measured in 19 channels during eyes closed, awake recording sessions, unless otherwise noted. Reported changes are in absolute power relative to normal controls, unless otherwise noted*.

*^b^Montgomery et al. (1991) noted a decrease in theta power of the same subject from day 0 to 6 weeks post-concussion suggesting that theta power may have increased as a result of the concussion and was trending back to normal 6 weeks later as the patient recovered*.

*^c^Watson (1995) found that for channel pairs T4–T6, P4–O2, P3–O1, the theta/alpha ratio had decreased from day 0 to day 10 post-injury for the same subjects suggesting that the ratio may have increased as a result of the concussion and was trending back to normal 10 days later as the patient recovered*.

Several additional studies from the literature index investigated spectral measures, but did not compare results to a control group. For example, Montgomery et al. (1991), Slobounov et al. (2012), von Bierbrauer et al. (1992), and Watson (1995) reported spectral changes in injured patients over time (theta reduction, alpha power reduction plus theta/alpha ratio changes, and alpha/theta ratio changes, respectively). Contradicting these studies, Chen et al. (2006a) reported all spectral power measures returned to normal levels after a three month retest (alpha1, theta/alpha1 ratio, theta/alpha2 ratio, and alpha1/alpha2 ratio). These results suggest spectral analyses may have utility for longitudinal evaluations, but confirmatory experiments must be matched with respect to time post-injury and recording condition.

Additionally, some studies reported no spectral differences between injured and control groups when a broad frequency window was used (Haglund and Persson, 1990; Coutin-Churchman et al., 2003; Gosselin et al., 2009). Such findings suggest power spectral analysis using sub-bands is preferred to observe significant differences in mTBI patients. We note that the patient group in the Coutin-Churchman et al. (2003) study included individuals with a wide range of psychiatric disorders (only 4/340 patients were classified with “post-traumatic headache”) and patients were not controlled for medication usage. Therefore, the study should only be considered a demonstration of the ability of spectral analysis to uncover dysfunction across many neurological conditions. Finally, Williams et al. (2008) reported higher variability in power in mTBI patients compared to normal participants (sigma, *p* = 0.005; theta, *p* = 0.019; delta, *p* = 0.008), but only limited ability of power spectra analysis to differentiate among injured and normal. Refer to Table 2 for a full listing of the 15 studies reporting use of spectral analysis.

#### Research gaps and future directions (spectral analysis)

The variability of qEEG recording conditions across studies severely impedes the formulation of strong conclusions on spectral analysis utility or effectiveness. While studies strongly suggest spectral analysis may be used as an mTBI detection tool, efforts should be made to corroborate previous findings using high-quality studies with controlled recording conditions and injured group selection criteria. In addition, the use of smaller electrode montages should be emphasized if field-based qEEG recording devices are expected to be preferred for U.S. military operations.

Spectral power averaged over all electrode sites is a global measure. It provides no information about the energy generated at individual sites or about the connectivities of different sites. This more detailed information is provided, in part, by functional connectivity measures, such as coherence and phase difference (see below), and by inter- and intra-hemispheric power asymmetries. Research should be devoted to identify the most promising spectral measures, but with the overall goal to use spectral measures in combination with other measures.

#### Summary of analysis (spectral analysis)

There are some examples in the literature suggesting that spectral analysis can be used to detect mTBI. Studies generally report altered alpha, delta, beta, and theta power as potential indicators of mTBI. However, while trends of specific spectral power alterations are apparent (decreased alpha and increased delta, beta, and theta in injured groups), the literature contains a high degree of contradictory results. This uncertainty is most likely due to poor study design and a lack of recording condition consistency. Therefore, additional studies with carefully controlled conditions must be conducted to identify the best spectral measures for mTBI detection. We anticipate spectral analysis would not be a sufficient detection tool in isolation, and, in fact, multiple studies suggest improved accuracy only when combined with other qEEG measures (Thatcher et al., 1989, 1991; Trudeau et al., 1998; Thornton, 1999, 2003; McCrea et al., 2010; Barr et al., 2012; Prichep et al., 2012a,b). Additionally, there is some evidence that depression is also marked by a decrease in alpha power and an increase in beta power similar to those seen in mTBI patients (Thornton, 2003). This finding raises the possibility of confounding test results in patient populations that may be prone to depression or PTSD. In summary, spectral analysis in and of itself does not appear to be a viable tool for mTBI detection.

### Functional connectivity measures

#### Definition (functional connectivity)

Analysis of functional connectivity quantifies the relationships between EEG signals recorded simultaneously at different sites. EEG connectivity measures fall into three groups: time domain measures, frequency-domain measures, and measures calculated from the geometry of embedded data.

Time domain measures quantify the correlation between EEG time series. They can be used to assess the connectivity of discrete feedback loops and the stereotyped propagation of signals across neural networks (Koenig et al., 2005). There are four commonly used time domain measures of functional connectivity to assess the correlation between pairs of observations: (1) Pearson product moment correlation, (2) Spearman rank order correlation, (3) Kendall rank order correlation, and (4) mutual information. Additional time domain measures of note include a normalization of mutual information [recently constructed by Reshef et al. (2011)] and EEG microstate topography, in which the occurrence of spontaneous short-lasting brain states is assessed (Koenig et al., 2005).

Frequency-domain measures quantify the correlation between spectra rather than between the original time series, or quantify the relationship between the phases of two signals. Variations on these themes that have been applied to EEG signals include coherence (Nunez et al., 1997, 1999), phase synchronization (Aviyente et al., 2011), phase locking index (Hurtado et al., 2004; Sazonov et al., 2009; Stam et al., 2009), phase locking value (Lachaux et al., 1999), imaginary coherency (Nolte et al., 2004; Stam et al., 2007), synchronization index (Gross et al., 2004), and phase lag index (Stam et al., 2007, 2009).

Measures of embedded data are more recent additions to the assessment of functional connectivity. Consider a scalar voltage time series measures at a scalp electrode, (*v*_1_, *v*_2_, …, *v*_N_). This time series is used to construct points in m-dimensional space, *Z*_i_ = (*v*_i_, *v*_i+1_, …, *v*_i+(m−1)_). While there are more complicated versions of the embedding process, in each version the original time series in this example becomes a directed trajectory in m-dimensional space, as motivated by the Takens embedding theorem (Takens, 1981). This powerful result demonstrates that, to an approximation, an intimate relationship exists between the geometry of the embedded set and the dynamical structure of the system that generated the observed time series. Now suppose two time series are measured simultaneously and used to generate two separate m-dimensional trajectories. Functional connectivity between the two original time series may be assessed by constructing measures relating the two trajectories. Examples include synchronization likelihood (Stam and van Dijk, 2002; Wendling et al., 2009) and cross recurrence diagrams (Romano et al., 2004; Richardson et al., 2008).

Distinction must also be made between connectivity measures and measures of causal relationships. Functional connectivity measures are computed from voltage signals recorded at different electrodes to determine if there is a relationship between two signals. These measures are adirectional. In contrast, causality measures specifically assess the direction of information movement. Measuring causality for mTBI detection is examined in Section “Analysis of Causal Relationships” of this report.

#### Utility for mTBI detection (functional connectivity)

Alterations in the functional connectivity of multichannel EEG have been reported in a range of neuropsychiatric disorders, including TBI (Table 3). This suggests reasonable utility of using functional connectivity measures for mTBI detection. It should be noted, however, that multichannel EEG usually requires more complex electrode montages and advanced computational capacity for data analysis. These features may be contrary to simple, lightweight devices capable of field-based use and easy operation. Additionally, the signal analysis and neuroinformatics challenges of these investigations should not be underestimated (Irimia et al., 2009, 2012; Goh et al., 2014).

**Table 3:** **Pathological conditions associated with altered functional connectivity (adapted from Bonita et al., 2014)**.

Disorder/dysfunction	Relevant publications	
Alzheimer’s disease	Georgopoulos et al. (2007)	Rosenbaum et al. (2008)
	Güntekin et al. (2008)	Stam et al. (2006, 2007, 2009)
	Locatelli et al. (1998)	Zhou et al. (2008)
Epileptic seizures	Ponten et al. (2007)	
Intra-arterial amobarbital injection	Douw et al. (2010)	
Autism spectrum disorder	Belmonte et al. (2004)	Murias et al. (2007)
	Just et al. (2004)	Rippon et al. (2007)
	Kana et al. (2007)	Vidal et al. (2006)
Brain tumors	Bartolomei et al. (2006)	
	Bosma et al. (2008)	
Multiple sclerosis	Georgopoulos et al. (2007)	
	Lenne et al. (2012)	
Preterm birth	Mullen et al. (2011)	
PTSD	Lanius et al. (2004)	
	Shaw (2002)	
Schizophrenia	Breakspear et al. (2004)	Lynall et al. (2010)
	Georgopoulos et al. (2007)	Michelyannis et al. (2006)
	Lawrie et al. (2002)	Symond et al. (2005)
Stroke	Grefkes and Fink (2012)	
Traumatic brain injury	Cao and Slobounov (2010)	Kumar et al. (2009a,b)
	Castellanos et al. (2010, 2011a,b)	Nakamura et al. (2009)
	Ham and Sharp (2012)	Sponheim et al. (2011)
	Kasahara et al. (2010)	Tsirka et al. (2011)

#### Evidence of efficacy (functional connectivity)

The various functional connectivity measures described above offers specific advantages and disadvantages as detection tools of neurological dysfunction. For example, Bonita et al. (2014) recently compared the four classical measures of time domain connectivity (Pearson product moment correlation, Spearman rank order correlation, Kendall rank order correlation, and mutual information) with multichannel EEGs obtained in two behavioral states (eyes open, no task and eyes closed, no task) from healthy control participants. It was found that mutual information distinguished between behavioral states with less data and was more robust to noise. This result was not unanticipated since mutual information is a non-linear measure that captures relationships not detected by the other three time domain measures. In addition, time domain measures have been most commonly applied to event-related data, since common reference points in time are often required for calculations (Koenig et al., 2005). EEG microstate topography analysis, however, does not require external time reference points and may be used with spontaneous qEEG recording to assess individuals with varied cognitive states or dysfunctions (Koenig et al., 1999; Lehmann et al., 2005; Müller et al., 2005). Frequency-domain measures are not robust to naïve application. For example, Schiff (2005) showed coherence calculations can produce misleading results and Guevara et al. (2005) reported synchronization values can be sensitive to the common reference signal used in EEG recordings. Insofar as we know, a comparative study of frequency-domain measures, analogous to the Bonita et al. (2014) time domain measure study, has not been conducted. Similarly, essential comparative studies have not been reported for measures of embedded data.

Specific evidence related to mTBI detection is relatively sparse: only three studies from the refined literature index investigated the efficacy of functional connectivity measures (see Table 4). Sponheim et al. (2011) reported reduced synchrony between specific frontal electrode positions for delta, beta, and gamma frequency bands. The results were statistically significant with a strong criterion (limited to *p* < 0.05 for all comparisons; Cohen’s *d* = 0.92–1.20), but we note the study used small sample group sizes (nine injured and eight normal) and follow-up studies with larger groups could improve significance. Interestingly, the use of neurocognitive assessment tools (battery of multiple tests) failed to properly classify individuals as injured or uninjured, suggesting that this new method may uncover dysfunctions below the thresholds of current mTBI detection methods. Thatcher et al. (1991) reported EEG phase was reasonably accurate in predicting outcome scores of patients 1 year post-injury (90.2% accuracy). This study also ranked the prognostic strength of EEG phase and EEG coherence above CT scan and spectral relative power. The Thatcher et al. (1991) study was longitudinal and no control group was used to assess diagnostic capacity of the functional connectivity measures. Finally, Thornton (2003) used an audio memory task paradigm and reported that phase and coherence were lowered for beta1 and beta2 spectral bands (*p* < 0.05 for each test). This study involved reasonable sample groups (85 injured, 56 controls), but has been challenged since 27 patients from the mTBI group were on medications and there was a statistically significant difference in age between the groups. Nevertheless, this study suggests functional connectivity should be further investigated as a diagnostic approach for mTBI. Details on the three studies investigating functional connectivity in mTBI are presented in Table 4.

**Table 4:** **mTBI studies using connectivity measures**.

General study information (spectral analysis)	Study design				Results	
	mTBI group (*n*)	Control group (*n*)	qEEG recording condition	Criteria for TBI patient	Measures	Statistical test/comparison/significance
Sponheim et al. (2011) “Evidence of disrupted functional connectivity in the brain after combat-related blast injury” *Notes*: this study uses a new method for calculating phase synchrony in qEEG recordings, and identifies several statistically significant synchrony disruptions in mTBI patients. Neurocognitive assessment tools (battery of multiple tests) were unable to distinguish between groups	9	8	Eyes closed, resting, wakeful	American Congress of Rehabilitation Medicine Special Interest Group on Mild Traumatic Brain Injury and the concussion grading system by the American Academy of Neurology	EEG synchrony between F7 and Fp2 (reduced)	Wilcoxon ranksum test and Cohen’s *d*
						1. Delta frequency: *p* < 0.05, *d* = 1.19
						2. Theta: *p* < 0.05, *d* = 1.07
						3. Beta-1: *p* < 0.05, *d* = 1.06
						4. Beta-2: *p* < 0.05, *d* = 1.20
					EEG synchrony between F7 and F4 (reduced)	Wilcoxon ranksum test and Cohen’s *d*
						1. Delta: *p* < 0.05, *d* = 1.00
					EEG synchrony between F8 and Fp1 (reduced)	Wilcoxon ranksum test and Cohen’s *d*
						1. Gamma: *p* < 0.05, *d* = 0.92

Thatcher et al. (1991) “Comprehensive Predictions of Outcome in Closed Head-Injured Patients” *Notes*: a gradient of prognostic strength of diagnostic measures was EEG phase > EEG coherence > GCS-*T* > CT scan > EEG relative power	162	None	N/A	Closed head injury and admittance to Neurotrauma hospital center	EEG phase	Prediction accuracy (extreme outcome scores) (1 year) 90.2%
						Correlation (intermediate scores) *R* = 0.664

Thornton (2003) “The electrophysiological effects of a brain injury on auditory memory functioning. The QEEG correlates of impaired memory” *Notes*: twenty-seven patients of the mTBI group were on various medications. Age was statistically different between groups (mTBI group was slightly older)	85	56	Eyes closed, resting, wakeful, during auditory memory task Eyes open, resting, wakeful, during auditory memory task	Loss of consciousness <20 min	Connectivity measures (includes phase and coherence)	*t*-Test 1. Lowered beta 1 phase and coherence (*p* < 0.05) 2. Lowered beta 2 phase and coherence (*p* < 0.05)

#### Research gaps and future directions (functional connectivity)

Since a limited number of studies have specifically investigated detection efficacy of mTBI, future research should be directed to corroborate previous findings. Concerns of study quality are abundant; therefore, future studies should ensure robust study design and limited reliance on medicated patient groups. The signal processing methods for coherence should also be investigated, since studies have reported misleading results depending on the analysis method.

#### Summary of analysis (functional connectivity)

In the context of mTBI, functional connectivity has not been investigated to the same extent as spectral analysis. The small number of available studies report that coherence may be a useable measure for accurate mTBI detection. However, the studies are fundamentally weak in their experimental design and need to be corroborated. Furthermore, the strongest positive results were reported for a longitudinal study. Similar to spectral analysis, coherence may be best suited as a contributing measure of a discriminant function for mTBI detection.

### Discriminant functions

While not specific to qEEG, discriminant functions were identified during the literature search as a common methodology for mTBI detection. Discriminant functions in this context comprise more than one qEEG measure and so deserve consideration here. Similar to the evaluation of spectral analysis and functional connectivity, discriminant functions are defined below, followed by an analysis of the evidentiary support for use as an mTBI detection tool.

#### Definition (discriminant functions)

The term “discriminant functions” refers to an entire class of analysis approaches in which a specific model is used to classify a set of data as belonging to a specific group (or not). Broadly defined, this involves the use of a specific rule (or set of rules), which define members of a class, and applying the rule to a set of data where membership is unknown in order to classify where the target member belongs. This is most commonly a discrete decision, but can even be used to estimate the location within a categorized continuum. Within the domain of TBI, discriminant functions may be used to classify a patient based on level of severity (e.g., severe TBI vs. mTBI) or the presence of injury (e.g., TBI vs. no injury) through the combination of various metrics (such as spectral or functional connectivity features) within a single discriminant model.

#### Evidence of efficacy (discriminant functions)

Nine studies were identified that investigate the utility of discriminant function analysis of qEEG data for the detection of mTBI (Table 5). Four studies investigate the efficacy of the same discriminant function currently under development by BrainScope, Inc. (McCrea et al., 2010; Naunheim et al., 2010a,b; Naunheim and Casner, 2010; Barr et al., 2012; O’Neil et al., 2012; Prichep et al., 2012a,b). McCrea et al. (2010) introduces the BrainScope method and device in which seven qEEG features comprise the discriminant function. The group does not provide complete details in this paper, but report the discriminant function consists of measures of asymmetry, coherence, and spectral power in the beta frequency range. Of note, the device only acquires data from three frontal electrode sites, and thus discrimination cannot be based on large network interactions. Twenty-eight concussed athletes were compared to age-matched controls and the data indicated statically significant differences between injured and control groups immediately after injury and 8 days post-injury, but not at day 45 post-injury. While specifics of the discriminant function are missing, the study is sound and provides evidence that the BrainScope discriminant function is efficacious as a detection tool immediately after injury.

**Table 5:** **mTBI studies using discriminant functions**.

General study information (spectral analysis)	Study design				Results	
	mTBI group (*n*)	Control group (*n*)	qEEG recording condition	Criteria for TBI patient	Measures	Statistical test/comparison/significance
Barr et al. (2012) “Measuring brain electrical activity to track recovery from sport-related concussion” *Notes*: this study provides further evidence for the BrainScope discriminant function as a potential mTBI diagnostic tool and may suggest utility after acute phases of injury. Commercial funds (BrainScope) were used to complete this study	59	31	Eyes closed, resting, wakeful	Concussion (diagnosed at hospital admittance)	BrainScope index (proprietary discriminant function index)	*t*-Test, corrected for unequal *n*’s (TBI vs. control)
						1. Day of injury (*t* = 3.75, *p* = 0.0004)
						2. Day 8 (*t* = 2.76, *p* = 0.008)
						3. Day 45 (*t* = 1.49, *p* = 0.15) (NS)
					Course of recovery (day of injury to day 8–45)	One-way ANOVA (TBI group) 1. Day of injury compared to day 8 and day 45 (*F* = 3.2; *p* = 0.046)
					Course of recovery, control group	One-way ANOVA (control group) 1. Day of injury compared to day 8 and day 45 (*F* = 0.5; *p* = 0.67) (NS)

Leon-Carrion et al. (2008) “A QEEG index of level of functional dependence for people sustaining acquired brain injury: the Seville independence index (SINDI)” *Notes*: investigates validity and reliability of a qEEG discriminant function for evaluating patient functional dependence. This function is less likely to be accurate for various levels of mTBI severity	40 (TBI)	40 (TBI patients, FIM + FAM score binning)	Eyes closed, resting, wakeful	CT or MRI scan	Mean SINDI score	One-way ANOVA Group 1 vs. Group 2 vs. Group 3 (*F* = 48.9, *p* < 0.0001)
		Group 1: complete dependence			Correlation between new discriminant and FIM + FAM scores	Pearson coefficient *R* = 0.81 (*p* < 0.001)
		Group 2: modified dependence Group 3: independence			Correlation between predicted scores from multiple regression and FIM + FAM scores	Pearson coefficient *R* = 0.85 (*p* < 0.0001)
					Leave-one-out method (discriminant function validation)	Classification accuracy (96.65%)
					External validation (33 patient group)	Classification accuracy (75%)

McCrea et al. (2010) “Acute effects and recovery after sport-related concussion: a neurocognitive and quantitative brain electrical activity study” *Notes*: this study used the BrainScope device, currently under commercial development. The BrainScope TBI index discriminant is based on 7 qEEG variables identified as good measures for comparison and differentiation (by ANOVA) and converted to standard *z*-scores	28	28	Eyes closed, resting, wakeful Frontal electrode site data acquisition only (FP1, FP2, AFz)	Concussion diagnosis (using American Academy of Neurology Guideline for Management of Sports Concussion)	Multivariate discriminant (BrainScope TBI index) combining various measures across frequency bands (main variables included measures of asymmetry, coherence, high beta power, and low beta power)	MANOVA
						Comparison between injured and control groups
						1. Baseline: *F* = 1.65, *p* = 0.164 (NS)
						2. At injury: *F* = 4.4, *p* = 0.002
						3. Day 8: *F* = 2.53, *p* = 0.04
						4. Day 45: *F* = 0.6, *p* = 0.74 (NS)

O’Neil et al. (2012) “Quantitative brain electrical activity in the initial screening of mild traumatic brain injuries” *Notes*: this study describes the BrainScope Index (a qEEG discriminant function) as an effective screening tool for emergency room patients with suspected TBI. Note that BrainScope funded this study and authors are BrainScope employees or consultants	119	None	Eyes closed, resting, wakeful	American congress of rehabilitation criteria	TBI-Index	Sensitivity = 94.7
						Specificity = 49.4
						Positive predictive value = 47.4
						Negative predictive value = 95.3
						Positive likelihood value = 1.92
						Negative likelihood value = 0.10
						Odds ratio = 18.5
					TBI-Index plus New Orleans Criteria (NOC)	Sensitivity = 97.0
						Specificity = 50.6
						Positive predictive value = 48.05
						Negative predictive value = 97.62
						Positive likelihood value = 1.97
						Negative likelihood value = 0.06
						Odds ratio = 36.1
					Correlation between TBI-Index and NOC	Pearson correlation
						*R* = 0.33, *p* < 0.0001

Prichep et al. (2012a) “Classification of traumatic brain injury severity using informed data reduction in a series of binary classifier algorithms” *Notes*: this method provides evidence for algorithms that can be used in portable devices to effectively score suspected mTBI patients based on qEEG recording analysis. The sensitivities and specificities were reported to be better than neurocognitive assessment tools, such as the MACE. A wide-scale multicenter trial is underway	633	None	Eyes closed, resting, wakeful	Glasgow coma scale >8 Category 1: no evidence of acute injury and no visible CT scan abnormality) Category 2: no visible CT scan abnormality; neurocognitive assessment abnormalities Category 3: presence of acute injury (visible CT scan abnormality)	Custom algorithm 1 (captures wide range of measures)	Classification of Category 3 from Categories 1 and 2
						Sensitivity = 96.3%
						Specificity = 77.5%
						Positive predictive value = 47.1%
						Negative predictive value = 99.0%
						AUC = 0.911
						Cohen’s *d* = 1.94
					Custom algorithm 2 (captures wide range of measures)	Classification of Category 1 from Categories 2 and 3
						Sensitivity = 80.5%
						Specificity = 73.9%
						Positive predictive value = 85.4%
						Negative predictive value = 66.5%
						AUC = 0.797
						Cohen’s *d* = 1.29

Thatcher et al. (2001) “An EEG Severity Index of Traumatic Brain Injury” *Notes*: Nuwer et al. (2005) criticizes this paper indicating that factors such as drowsiness and medication are well known to effect EEG results, contrary to the results of this study	108 (40 mTBI, 25 moderate TBI, 2 severe TBI) 503 (cross-validation)	None	Eyes closed, resting, wakeful	mTBI subgroup: Glasgow coma score 13–15; posttraumatic amnesia <1 h; loss of consciousness <20 min	EEG severity index (discriminant function)	Classification accuracy
						mTBI accuracy = 95.08%
						Pearson correlation
						1. EEG index and neuropsychological performance (various tests) (*p* < 0.05)
						*t*-Test
						1. Mild vs. moderate (*p* < 0.0001)
						2. Mild vs. severe (*p* < 0.000001)
						3. Moderate vs. severe (*p* < 0.00001)

Thatcher et al. (1989) “EEG discriminant analysis of mild head trauma” *Notes*: this study showed that EEG discriminant functions can be accurately (> 90%) be used to classify normal subjects vs. those with mild TBI. Nuwer contends that results from use of this discriminant function have not been published since 1989 and the TBI patients were on a variety of medications that could skew the results	608	108	N/A	Glasgow coma score 13–15; loss of consciousness <20 min	Thatcher discriminant function (comprises 20 measures) accuracy	1. 204 mTBI subjects vs. 83 controls (94.8% accuracy)
						2. 130 mTBI subjects vs. 21 controls (95.4% accuracy for mTBI, 90.5% for normals)
						3. Test-retest classification accuracy using 93 patients ranged from 77.8 to 92.3%
						4. Off-site test with 70 new patients (92.8% accuracy for TBI patients and 100% for normals)
					Thatcher discriminant score change (for injured patients) over 2 weeks	Pearson correlation
						*R*^2^ = 29.9%, *p* < 0.68 (NS)
					Thatcher discriminant score change (for injured patients) vs. number of days from injury to EEG test	Pearson correlation *R*^2^ = 48.3%, *p* < 0.38 (NS)

Thornton (1999) “Exploratory investigation into mild brain injury and discriminant analysis with high frequency bands (32–64 Hz)” *Notes*: introduces a discriminant function for mTBI based on high frequency frontal coherence (2945 variables). Author acknowledged the study as exploratory and preliminary. Combination of both Thatcher and new discriminant functions resulted in perfect accuracy	32	52	Eyes closed, resting, wakeful	Hit head on part of car during car accident; loss of consciousness <2 min or none	Thatcher discriminant (over 1 year post-injury)	Accuracy (79% for injured group)
					New high-frequency (up to 64 Hz) discriminant	Accuracy (89% for injured group across all time periods; 100% for injured group within 1 year of injury)
						False hit rate (normal sample = 52%
					New high-frequency discriminant plus Thatcher discriminant	Accuracy = 100%
					FP1 position variables	Levene’s test for homogeneity of variances *p* < 0.05 for all variables
					Relative power	
					Coherence theta	
					Coherence beta 2	
					Phase beta2	

Trudeau et al. (1998) “Findings of mild traumatic brain injury in combat veterans with PTSD and a history of blast concussion” *Notes*: this study provides data that suggests the Thatcher discriminant function can be used to differentiate previous blast-induced mTBI among veterans suffering from PTSD from those PTSD patients who did not suffer a blast mTBI. While the discriminant function was not able to accurately bin TBI vs. non-TBI, the sample size was small and the range in age and post-injury timeframe was quite large	27 (blast history)	16 (no-blast history)	Eyes closed, wakeful	PTSD patient with blast history (note: control group also PTSD patients)	Thatcher discriminant score	*t*-Test
						Blast group vs. no-blast group (*t* = 5.4, df = 41, *p* < 0.0001)
						*t*-Test
						Regrouping of TBI history vs. no-TBI history (*t* = 0.32, df = 41, *p* = 0.75) (NS)
						ANOVA
						ADHD, other mTBI history, TBI history, or psychoactive substance abuse (no significance)

The Barr et al. (2012) study is similar to the McCrea study, but recorded qEEG from a larger cohort of mTBI patients and healthy controls and used a five electrode montage. Similar results were reported as well; statistically significant discriminant function indices were observed on the day of injury and at day 8 post-injury, but not at day 45 post-injury. A larger difference (yielding higher significance criterion) was observed compared to the McCrea et al. (2010) study, implying that the discriminant function was altered compared to the McCrea version. However, no details were provided about the specific measures that comprise the function; thus this possibility cannot be confirmed.

O’Neil et al. (2012) also report on the utility of the BrainScope discriminant function, but do not use a control group. The study compares the classification outcome of the discriminant function (called the TBI-index) to classification by medical examination and CT scan. The sensitivity of the discriminant function was good (94.7%), but specificity and positive predictive value were poor (approximately 50%). Problems with the study design are not evident, but the results suggest improvement of the BrainScope discriminant function is required to attain reasonable specificity and avoidance of false negatives.

Prichep et al. (2012a) provides data from a large mTBI group of 633 patients. Although the study design was similar to O’Neil et al. (2012), the project was substantially improved by including comparison to other approaches and improving the complexity of the analysis. For example, Prichep et al. compared the BrainScope discriminant function to a more relevant group of common TBI assessment tools (e.g., military acute concussion evaluation) and inclusion of an “artifact detection module” to minimize EEG noise and artifacts. The discriminant function itself was significantly more complex and includes variables such as spectral analysis measures, information theory-based measures, scale-free brain activity measures, fractal dimension measures, functional connectivity measures, and various multivariate measures. Two algorithms were devised to separate various levels of severity of TBI and specificity was improved compared to O’Neil et al. (2012). Specifically, the new discriminant function was able to differentiate between mTBI and moderate/severe TBI patients with a sensitivity of 80.5% and a specificity of 73.9%. Positive predictive value was also improved compared to the O’Neil data (85.4%), but negative predictive value was poorer (66.5%). These results indicated the newer BrainScope discriminant function was able to detect mTBI, differentiate it from more severe forms, and decrease incidence of false negative classification. Overall, this paper provides excellent evidence that a well-designed discriminant function may be a serviceable mTBI detection tool.

Leon-Carrion et al. (2008) described another discriminant function not associated with BrainScope. However, this discriminant function, the Seville independence index (SINDI), was built to classify patients according to the level of functional dependence, such as complete dependence, modified dependence, and independence. These classifications are less meaningful for initial detection of injury, but rather are used to predict the level of care required during the rehabilitation phase. The discriminant function comprised nine qEEG variables including four coherence variables, three phase lag variables, one absolute amplitude variable, and one absolute asymmetry variable at specific frequency bands. Accuracy was 100% for classification between complete dependence (*n* = 15) and independence (*n* = 14), which may correlate with severe TBI and mTBI, respectively. In addition, the SINDI scores correlated well (*p* < 0.0001) with established tests of functional dependence (Functional Independence Measure + Functional Assessment Measure). Overall, this paper reports the qEEG-based discriminant function may be used to predict the level of functional dependence in injured patients, but does not investigate the diagnostic potential of SINDI.

The remaining papers in the literature index present results based on a collection of discriminant functions from the same group. The original discriminant function comprised 20 measures of coherence, phase, amplitude asymmetry, and relative power at various frequency bands (Thatcher et al., 1989). The function was reported to be >90% accurate in discriminating mTBI patients (Glasgow coma score 13–15; loss of consciousness <20 min) from healthy controls in a large dataset (total of 608 mTBI patients and 108 controls). It also reported good test–retest accuracy using 93 patients (77.8–92.3%). The paper, however, has received strong criticism since the TBI patients were on a variety of medications (Nuwer et al., 2005). The same group published another report on another discriminant function that used 16 measures of EEG coherence, phase, and amplitude asymmetry (Thatcher et al., 2001). Notably, none of the measures used in the 2001 study were the same as those in the 1989 study. This discriminant function was able to classify mTBI from moderate TBI with high probability of success (*t*-test, *p* < 0.0001). Overall classification accuracy of mTBI patients from controls was also high (95%). This study was criticized since it was assumed that medications did not alter EEG, even though such an effect is widely accepted in the field (Nuwer et al., 2005). Nuwer’s argument does not, however, question the overall study design or methodology [unlike the criticism leveled against Thatcher et al. (1989)]. The Thatcher et al. (1999) discriminant function was also tested in concert with another discriminant function and the authors found the discriminant to maintain reasonable classification accuracy (79%) over 1 year post-injury. Supplementation with a new high-frequency discriminant function improved accuracy to 100% in a sample group of 32 mTBI injured patients and 52 controls. Use of the high-frequency discriminant function alone was reported to provide not only high accuracy (89%) but also a high false hit rate (52%). These studies also suggest high potential value of using a discriminant function as a mTBI detection tool.

#### Research gaps and future directions (discriminant functions)

The primary challenge in the use of discriminant functions with qEEG is in deciding the most appropriate functions to use, which is directly tied to which features are most relevant. The problems here are threefold. First, there are an extremely large number, potentially hundreds, of possible analytic approaches and data features, which could be used as the basis for classification. These range from time-based measures, such as ERPs, to frequency-domain analytics, to more complex time-frequency hybrid features such as cross-channel phase relationships described above. Second, the function or class of functions used for discrimination must be relatively specific to the state being detected, so as to include both a high successful “hit” rate, and decrease as much as possible the occurrence of false-identifications. That is, in the example of mTBI, the functions must be precise enough to discriminate not only the occurrence of mTBI from no trauma but also the occurrence of trauma from other states such as extreme exhaustion, sleepiness, or cognitive fatigue, which may share similar response features. Finally, in most cases discrimination requires either some level of *ad hoc* adjustment or comparison against a known baseline in order to perform accurately for multiple individuals, due to the high variability between individuals, and between situations. Access to a state considered to be “ground truth” is required. While this is generally not a problem in research, gaming, or repeated-access medical applications in which the conditions of detection system can be predicted and controlled, access to the patient will most likely occur only after trauma has occurred.

#### Summary of analysis (discriminant functions)

qEEG-based discriminant functions represent a promising solution for detecting mTBI, since the use of multiple measures in the evaluation process improves specificity and sensitivity. Studies with large sample sizes have reported strong discriminatory abilities of discriminant functions (Thatcher et al., 2001; Prichep et al., 2012a). Criticism of earlier studies has been warranted due to the known effects of medication on the EEG, but improved study design of more recent studies (i.e., Prichep et al., 2012a) suggests discriminant functions may be considered highly valuable mTBI detection tools.

## Additional Measures Not Represented in the Down Selected qEEG Literature

Additional EEG-related technologies not represented in the original literature search are also relevant to mTBI detection and deserve consideration here. Many of these technologies are based on newer mathematical analysis techniques not previously investigated with respect to mTBI. Relevant technologies include assessment using ERPs, information dynamics, symbolic dynamics, analysis of causal relationships, and graph theory. Each technology is introduced below and includes a summary of the available evidence for mTBI detection utility based on additional literature reviews.

### Event-related potentials

Event-related potentials are stereotyped responses to a discrete sensory, cognitive, or motor event. An ERP is distinguished from an evoked potential (EP) by having a longer latency and in being altered by the cognitive significance of the stimulus to the subject. EPs may be referred to as “exogenous” because they are strongly dependent on the physical properties of the stimulus being used to elicit the response (*e.g*., auditory vs. visual stimulation). Conversely, ERPs may be described as “endogenous” in nature because, although they are also related to some external event, they are heavily influenced by higher level cognitive processes and are less dependent on the stimulus modality or physical characteristics of the stimulus. Can ERPs reliably diagnose mTBI? Gaetz and Bernstein (2001) reviewed the use of ERPs in the assessment of traumatic brain injury. Specifically, they considered alterations in the amplitudes and latencies of well characterized components of ERPs, for example, the P3, as indicators of injury. They concluded:

Visual P3 latency seems to be the most sensitive electrophysiologic procedure covered in this review. All studies using this technique to assess mTBI have found differences in P3 latency compared with normal controls. In addition, the P3 word technique may be very useful for the simultaneous assessment of PTSD, malingering, and brain injury. This procedure seems to be sensitive to injury while resistant to false positives when a 2.5 SD normal limit is used.

and,

An electrophysiologic assessment battery may be the most effective method to detect differences in MTBI subjects who experience cognitive dysfunction.

In addition, Lew et al. (2006) also suggested that “longer latency ERP components hold promise in predicting recovery of higher cognitive functions.” Since deficits in emotional processing may be an element in the clinical presentation of TBI, ERP investigations using emotionally valenced stimuli (Catani, 2003; Lew et al., 2005b; Solbakk et al., 2005) may be particularly informative with this patient population.

There are, however, two notable caveats that are unique to the collection of ERPs. First, because responses are analyzed in respect to the occurrence of a specific event, there is a necessity for a more complex data acquisition system than in the passive collection of qEEG data (Spencer, 2005). Specifically, a secondary device, system, or method must be available to provide the stimulus used to drive the ERP (examples include a PC and monitor, audio amplifier, LED driver, or vibrotactile stimulator). The stimulation device must be very tightly coordinated with the EEG data acquisition system (on a sub-millisecond time scale), so that accurate time-locking of the ERP to the original event(s) can be achieved. While this level of integration is not technically challenging, it adds bulk, complexity, and additional use effort on behalf of the device operator and requires special EEG acquisition hardware, which is designed for integration with other devices. It should be noted, however, that ERPs may be used in clinical settings with fairly minimalistic equipment.

A second caveat of ERPs that stems from the reliance upon a stimulation paradigm is the intimate, but somewhat limiting, specificity between the stimulus used and the type of brain activity, which will occur in response to that event. By definition, the response elected by an ERP is related to a specific event, and (in a good paradigm) will include response *only* to that event, because of the extreme complexity and high specificity of various sensory regions of the brain. For example, at least in a normal healthy brain, auditory-evoked-potentials will not activate the occipital cortex (which is primarily dedicated to vision), while visual-evoked-potentials tend to be isolated to this very region. As a result, the type of paradigm used to elicit ERPs is critical to their understanding and analysis, and leaves opportunity for abnormalities in other, non-stimulated brain regions to be overlooked. In the case of mTBI, which can manifest in a wide variety of manners and stem from many possible neurophysiological dysfunctions, it is a substantial challenge to identify a single paradigm, which might be sufficient to cover all, or even the majority of potential cases.

Besides the studies referenced above, a large number of other published reports have obtained ERPs from TBI patients (Table 6). However, the collective experience does not yet provide a definitive answer as to whether ERP is an accurate diagnostic tool (Rapp and Curley, 2012). To date, the examination of ERPs obtained from TBI patients has been limited to reports of differences in amplitude and latencies of identified components of the ERP.

**Table 6:** **Index of ERP studies of traumatic brain injury**.

Arciniegas and Topkoff (2004)	Gaetz and Weinberg (2000)	Olbrich et al. (1986)
Arciniegas et al. (2005)	Gaetz and Bernstein (2001)	Onofrj et al. (1991)
Baguley et al. (1997)	Geisler et al. (1999)	Papanicolaou et al. (1984)
Bennouna et al. (2007)	Giaquinto (2004)	Perlstein et al. (2006)
Broglio et al. (2009)	Gosselin et al. (2006)	Polo et al. (2002)
Campbell et al. (1986)	Gosselin et al. (2011)	Pontifex et al. (2009)
Campbell et al. (1990)	Gosselin et al. (2012)	Potter and Barrett (1999)
Campbell and DeLugt (1995)	Heinze et al. (1992)	Potter et al. (2001)
Catani (2003)	Kaipio et al. (1999)	Potter et al. (2002)
Chen et al. (2006a)	Kaipio et al. (2001)	Pratap-Chand et al. (1988)
Clark et al. (1992)	Kane et al. (1996)	Rappaport et al. (1990)
Cremona-Meteyard and Geffen (1994)	Keren et al. (1998)	Reinvang (1999)
Curry (1980)	Lachapelle et al. (2008)	Reinvang et al. (2000)
Curry et al. (1986)	Lavoie et al. (2004)	Reuter and Linke (1989)
Deacon-Elliott and Campbell (1987)	Lew (2001)	Rizzo et al. (1978)
Deacon-Elliott et al. (1987)	Lew (2005)	Rugg et al. (1988)
Deacon et al. (1991)	Lew et al. (1999)	Rugg et al. (1993)
Deacon et al. (1991)	Lew et al. (2002)	Sangal and Sangal (1996)
De Beaumont et al. (2007a)	Lew et al. (2003)	Segalowitz et al. (1992)
De Beaumont et al. (2007b)	Lew et al. (2004)	Segalowitz et al. (1997)
De Beaumont et al. (2009)	Lew et al. (2005a)	Segalowitz et al. (2001)
di Russo and Spinelli (2010)	Lew et al. (2005b)	Solbakk et al. (1999)
Drake and John (1987)	Lew et al. (2008)	Solbakk et al. (2000)
Duncan et al. (2003)	Lew et al. (2006)	Solbakk et al. (2002)
Duncan et al. (2005)	Lew et al. (2007a,b)	Solbakk et al. (2005)
Dupuis et al. (2000)	Mazzini (2004)	Spikman et al. (2004)
Dywan and Segalowitz (1996)	Mazzini et al. (1999)	Unsal and Segalowitz (1995)
Elting et al. (2008)	Mazzini et al. (2001)	Viggiano (1996)
Folmer et al. (2011)	Münte and Heinze (1994)	von Bierbrauer and Weissenborn (1998)
Ford and Khalil (1996a)	Olbrich et al. (1986)	Wang et al. (2004)
Ford and Khalil (1996b)	Onofrj et al. (1991)	Werner and Vanderzant (1991)
Gaetz et al. (2000)	Papanicolaou et al. (1984)	
	Perlstein et al. (2006)	

### Advanced signal processing technologies

This section provides evidence that the clinical utility of qEEG may be advanced significantly by expanding the set of measures that are computed from EEG signals. The mathematical field of dynamical systems theory has expanded dramatically during the past 30 years. Research activity is continuing and, indeed, accelerating. To date, the application of these new methods has been mostly confined to the analysis of physical and financial systems. These methods have not yet received systematic application in EEG. Four areas are particularly promising: information dynamics, symbolic dynamics, measures of causal relationships, and measures of network dynamics derived from graph theory.

#### Information dynamics

A digitized EEG signal is a sequence of voltage measurements. As classically defined, the Shannon entropy (Shannon, 1948) of this signal is insensitive to the sequential order of the voltage measurements. That is, if the voltage measurements are randomly shuffled, the same value of Shannon entropy will be obtained. Kolmogorov (1958, 1959) and Sinai (1959) extended the concept of entropy by constructing a generalization of the original concept that quantifies the sequential structure of a signal. Kolmogorov–Sinai (K–S) entropy has been described as the rate at which a system generates information (Eckmann and Ruelle, 1985), which may also be considered the central function of the CNS. K–S entropy and related measures provide the mathematical foundation of information dynamics (Deco and Schürmann, 2001). While K–S entropy is a limiting process requiring, by definition, an infinite amount of data, it is possible to construct approximations computed with finite data sets. The most commonly utilized approximations are approximate entropy (Pincus, 1991) and sample entropy (Richman and Moorman, 2000). Approximate entropy and sample entropy have been used to quantify EEG records (Bruhn et al., 2000; Abásolo et al., 2005; Sabeti et al., 2009; Yuan et al., 2011; Yun et al., 2012), heart interbeat interval sequences (Pincus and Goldberger, 1994; Ho et al., 1997), and human postural sway data (Ramdani et al., 2009). Insofar as we know, they have not been used in the analysis of EEGs obtained from TBI patients.

A variant of sample entropy is multiscale entropy (Costa et al., 2002). The original time series is used to construct a sequence of coarse-grained time series. The Scale 2 time series is obtained by averaging every two values. The Scale 3 time series is obtained by averaging every three values, and so on. The sample entropy is calculated for each time series to produce entropy as a function of temporal scale. The procedure was originally applied to the analysis of heart interbeat interval sequences and was found to discriminate between healthy controls and patients presenting congestive heart failure, atrial fibrillation or advanced age (Costa et al., 2002, 2005). The same method has been used in the analysis of EEG signals to discriminate between control participants and those with autism spectrum disorder (Bosl et al., 2011; Catarino et al., 2011), Alzheimer’s disease (Park et al., 2007; Mizuno et al., 2010), schizophrenia (Takahashi et al., 2010), and Parkinson’s disease (Chung et al., 2013) patients. Insofar as we know, multiscale entropy has not been used to analyze EEG records obtained from TBI patients. The approach was, however, used to analyze intracranial pressure records obtained from TBI patients where it was found that multiscale entropy correlates with outcome.

There are technical issues associated with estimation of multiscale entropy that require attention. Nikulin and Brismar (2004) argued (in our view, correctly) that the scaling length in the embedding space used to calculate sample entropy should be adjusted with the temporal scale of the time series. In their calculations with simulated data, the temporal scale dependence of entropy was lost when this modification was introduced. Costa et al. (2004) replied and argued that in the case of the heart interbeat interval data analyzed in their paper, scale dependence is preserved. Wu et al. (2013) noted that the coarse graining procedure used in Costa et al. (2002), and in other papers using the method, reduces the length of the data set with each increase in temporal scale length. Substituting a moving average retains much of the multiscale characteristics of the original implementation of multiscale entropy without introducing the loss of reliability that results from attempting entropy calculations with small data sets. Systematic validation calculations are required to confirm this assertion.

#### Symbolic dynamics

As will be seen, the terms entropy and complexity lose specificity, rather than gain it, as a function of time. Complexity measures derived from symbolic dynamics share the important property of sequence sensitivity with K–S entropy and its variants. As the term complexity is used here, there is a fundamental difference between symbolic dynamic measures and K–S entropy. Approximate entropy and sample entropy are computed directly from the voltage values. In the case of symbolic dynamic measures, the original sequence of voltages is converted to a symbol sequence. Complexity is a measure of the orderliness or lack of orderliness of this symbol sequence. The procedure for converting voltages to a symbol sequence can be simple. For example, the symbol “A” is entered into the sequence if the value of the voltage is greater than the median or the symbol “B” is entered if the voltage value is less than the median (note that the use of the median, rather than the mean, is statistically important). More elaborate procedures for generating a symbol sequence from a voltage are possible. A complicating clarification must be introduced, while the immediate emphasis here is on measures computed from symbol sequences, some of these measures, such as mutual information, can be recast in versions that utilize continuous variables such as voltages. Sequence sensitive measures of symbol sequences include topological entropy (Crutchfield and Packard, 1983), metric entropy [also in Crutchfield and Packard (1983)], forbidden word entropy (D’Alessandro and Politi, 1990), context free grammar complexity (Jiménez-Montanó, 1984), Lempel–Ziv complexity (Lempel and Ziv, 1976), mutual information (Gallager, 1968; Cellucci et al., 2006), *n*-th order entropy (Ebeling, 1997), and conditional entropy (Steuer et al., 2001).

Xu et al. (1994) found that the forbidden word complexity of EEG data is sensitive to the participant’s behavioral state (e.g., eyes open vs. eyes closed recording conditions), mental load, and sleep stage. Watanabe et al. (2003) found that EEG Lempel–Ziv complexity changes in response to work load and eyes open vs. eyes closed recording conditions. Symbol sequence complexity measures have also been used in the analysis of clinical EEG records including assessment of schizophrenia and depression (Li et al., 2008; Sabeti et al., 2009), diagnosis of Alzheimer’s disease (Abásolo et al., 2006), anesthetic depth (Fan et al., 2011), and seizure detection (Hu et al., 2006). Our limited literature review on symbolic dynamics did not locate papers using these methods in the analysis of EEG data obtained from TBI patients.

#### Analysis of causal relationships

The measures of functional connectivity described in Section “Functional Connectivity Measures” are used to establish the degree of association between two simultaneously recorded signals. However, these measures are insensitive to the direction of information movement. Measures of causal relationship extend the analysis of functional connectivity by addressing directionality. The operationalization of causality that was used to construct mathematical measures of directionality was published by Wiener in 1956 (Wiener, 1956). Stated simply, if measuring Signal A improves the prediction of Signal B, then the process generating Signal A is, to some degree, a causal driver of the process generating Signal B. This relationship is not necessarily unidirectional, as measuring Signal B may also improve the prediction of Signal A. The relative magnitude of these relationships can be used to define net information flow.

Mathematical methods of assessing the direction of information movement fall into four groups: linear regression methods (Granger, 1969; Sims, 1972; Kaminski et al., 2011), information theory methods (Kaneko, 1986; Vastano and Swinney, 1988; Schreiber, 2000), frequency-domain methods (Baccalá and Sameshima, 2001; Nolte et al., 2008), and methods constructed with embedded data (Rulkov et al., 1995; Le Van Quyen et al., 1998, 1999; Arnhold et al., 1999).

There is a prior literature describing the application of these methods to scalp EEG signals (Inouye et al., 1983, 1993; Mars and Lopes da Silva, 1983; Mars et al., 1985; Hesse et al., 2003; Chen et al., 2006b). It would seem plausible to speculate that TBI alters network relationships, and that post-injury alterations of information flow direction would provide a sensitive assessment of CNS damage. Unfortunately, this optimistic anticipation has not been realized. To this end, several problems must be addressed. The first set of difficulties is specific to EEG analysis. Haufe et al. (2011) showed that because of volume conduction, all EEG channels drive each other. Their simulations indicate that Granger causality used with the usual statistical tests can lead to spurious relationships. The same authors subsequently constructed procedures that can, to a degree, address these difficulties (Haufe et al., 2013). The second set of difficulties exists independently of the special problems associated with EEG. Namely, mathematical analysis shows that spurious identifications of causality can occur (He and Maekawa, 2001; Breitung and Swanson, 2002; Albo et al., 2004).

An additional complication should be considered: causal relationships are not static. Consider two processes, Process A and Process B. Suppose that Process A is a strong causal driver of Process B from time *t*_0_ to time *t*_1_. Now suppose this relationship reverses and Process B is a strong driver of process A from time *t*_1_ to time *t*_2_. If the analysis is conducted over time *t*_0_ to time *t*_2_, evidence of any causal relationship may be lost. Therefore, the identification of transitions between domains of dynamical behavior is a first step in causal analysis. The identification of transitions between different dynamical regimes is not, however, as straightforward as one might suppose, and this characteristic difficulty is particularly true of EEG data. While noise contamination can be a main cause, the problem can be more subtle. For example, transitions in CNS activity form a temporal hierarchy (Kiebel et al., 2008; Perdikis et al., 2011a,b; Papo, 2013), and a transition that is dynamically significant on a time scale of 1 s may not be significant on a time scale of 1 min (Rapp et al., 2013b). Causal relationships will show similar time-scale dependence. Therefore, as is often the case, the translation of an elegant mathematical concept to practical application in EEG can be more difficult than initially anticipated.

#### Graph theory

Viewed mathematically, a network is a collection of nodes and edges (lines). When applied to EEG, the nodes are electrodes and the edges are the connections between them. There are three possible characterizations of edges. In the simplest case, the edge is either present or absent. This is implemented by selecting a connectivity measure from the previously described measures and selecting a threshold. A connection between two electrodes is deemed to be present if the connectivity measure’s value exceeds the threshold and absent if the value is less than the threshold. A second possible characterization of an edge is a weighted edge where the strength of the connection between two electrodes is determined by the value of the selected connectivity measure. The third characterization of an edge is the directed edge where, in addition to a weighting, the direction of information transfer is determined using a causality measure.

Once the geometry of the network has been determined, it is possible to use the methods of graph theory to provide a concise, quantitative description. The most commonly employed metrics are the clustering coefficient and the path length (Watts and Strogatz, 1998). The validity of these measures in characterizing the CNS is suggested by Smit et al. (2008). The investigators examined EEG records from 574 monozygotic or dizygotic twins and found that 46–89% of individual differences in the clustering coefficient and 37–62% of individual differences in path length are heritable. Alterations of network geometries as determined by EEG, magnetoencephalography (MEG), or MRI have been found in clinical populations, including TBI patients (Table 7).

**Table 7:** **Studies reporting altered network geometries in neuropsychiatric disorders**.

Disorder/dysfunction	Relevant publications
Alzheimer’s disease	Stam et al. (2007)
CNS tumor	Bartolomei et al. (2006)
Depression	Leistedt et al. (2009), Park et al. (2014)
Epilepsy	Bernhardt et al. (2011), Ponten et al. (2007), van Dellen et al. (2009)
Schizophrenia	Li et al. (2008), Rubinov et al. (2009)
Traumatic brain injury	Cao and Slobounov (2010), Castellanos et al. (2011a), Castellanos et al. (2011b), Nakamura et al. (2009), Tsirka et al. (2011), Zourdakis et al. (2011), Irimia et al. (2013a), Irimia et al. (2013b), Goh et al. (2014)

Once again, however, initial enthusiasm must be tempered by analyses showing spurious identifications of network properties. Bialonski et al. (2011) examined binary networks (an edge is either present or absent) constructed by a simple threshold criterion. These results suggest that “small world” topologies in networks constructed from empirical data “may be partly or solely due to finite length and frequency content of time series.” They suggest that random networks constructed from surrogate data may provide important comparisons for results obtained from multichannel EEG [surrogate data calculations are an important statistical procedure for validating the results obtained with experimental data, Theiler et al. (1992), Prichard and Theiler (1994)]. It should be noted, however, that inappropriately constructed surrogates can result in the false-positive rejection of the surrogate null hypothesis, that is, the procedure can indicate the presence of deterministic non-linear structure in a signal constructed from linearly filtered random numbers (Rapp et al., 2001). Blinowska and Kaminski (2013) found that networks constructed from adirectional measures (correlation, coherence, mutual information, synchronization likelihood, transfer entropy) can result in spurious correlations. Indeed, they report that spurious correlations can outnumber true correlations. Giving all connections an equal weight (a binary network) exacerbates this problem. They recommend directional, causally dependent measures (directed transfer function, partial directed coherence), but as noted above volume conduction in the CNS introduces significant problems when directional measures are applied to EEG signals.

## Discussion

### Controversy within the qEEG field

The earlier literature describing the application of qEEG technologies in the assessment of TBI has been marked by strident disagreement. In 1989, Thatcher et al. published a study based on EEG records obtained from 608 patients with mTBI and 108 healthy control participants. In this study, qEEG-derived measures discriminated between the two groups with a sensitivity of 96.6% and specificity of 89.1% (as outlined presently, the reports of specificity require reconsideration). Subsequently, the same group constructed a qEEG-based Severity Index of TBI and obtained a sensitivity of 95.5% and specificity of 97% (Thatcher et al., 2001).

In 1997, in part in response to the Thatcher et al. (1989) report, a highly critical review of clinical qEEG was published (Nuwer, 1997). Nuwer concluded “on the basis of current clinical literature, opinions of most experts, and proposed rationales for their use, qEEG remains investigational for clinical use in post-concussion syndrome, mild or moderate head injury, learning disability, attention disorders, schizophrenia, depression, alcoholism, and drug abuse…” Nuwer continued, “Because of the very substantial risk of erroneous interpretations, it is unacceptable for EEG brain mapping or other qEEG techniques to be used clinically by those who are not physicians highly skilled in clinical EEG interpretation.”

Vigorous responses to the 1997 Nuwer paper were published by Thatcher et al. (1999), and by Hoffman et al. (1999). The Nuwer paper has been an element in court actions, for example, County Court at Law No. 1, Travis County Texas, Cause No. 227,520. As summarized by Thatcher et al. (2003), the judge in the Travis County case admitted qEEG evidence in a TBI case and denied admission of the 1997 Nuwer paper (see also Zawski v. Giggs). In 2005, Nuwer et al. returned to the subject and focused specifically on the application of qEEG in the assessment of TBI. Nuwer et al. concluded that “overall the disadvantages of qEEG panels and diagnostic discriminants presently outweigh the advantages of these studies in the diagnosis of MTBI.” In spite of this assessment, qEEG as a potential TBI detection tool has still received a great deal of attention since Nuwer’s 2005 paper. This more recent research argues against an unreservedly negative assessment of qEEG utility. Indeed, while the evidence supporting the sole use of qEEG (as defined by the TWG) for the assessment of mTBI is weak it would be unwarranted to conclude that EEG is not a valued metric in the search for the proper assessment protocol for mTBI. Upon expansion of the definition of “qEEG” to include other EEG-based datasets (e.g., ERPs and EPs), EEG will most likely be a necessary component of any assessment protocol for mTBI. In addition, evidence suggests that the value of qEEG in the assessment of mTBI will increase as the methods used in the analysis of these signals become more sophisticated.

Evidence supporting the value of more elegant analysis procedures for EEG in the assessment of mTBI comes in part from McCrea et al. (2010), Prichep et al. (2012a,b), and Tsirka et al. (2011). Prichep et al. (2012a,b) and McCrea et al. (2010) used a modest EEG montage to collect short epochs of EEG that were subjected to rigorous artifact detection algorithms: traditional and non-traditional univariate and multivariate analysis procedures that feed into an informed data reduction protocol. This protocol controls for replicability, over-fitting, and cross-validation of metrics. In addition, we see the value of integration of various demographic, neurocognitive, and self-report datasets along with the physiological datasets to achieve increased sensitivity and specificity for assessment of mTBI. Tsirka et al. (2011) adds positive assessment value to this approach by using small world network metrics, which utilize mean power analysis, and synchronization likelihood parameters to assess suboptimal network organization in EEGs obtained from mTBI victims.

### Specificity of EEG based mTBI detection

Published reports suggest that dynamical measures calculated from EEGs are altered following TBI, that is, the sensitivity of these measures to TBI is supported. Reports of high specificity must, however, be interpreted with care. While high specificities have been obtained in carefully constructed clinical studies, which compare TBI patients against healthy control participants, dynamical measures calculated from EEG records thus far are not specific to TBI. This lack of specificity is indicated by the results summarized in Table 8. Altered EEG synchronization is observed following traumatic brain injury, but it is also observed in most neuropsychiatric disorders. Therefore, while it may be possible to discriminate between TBI patients and healthy individuals using these measures alone, it will probably not be possible to discriminate between TBI and depression, schizophrenia, or several other clinical conditions.

**Table 8:** **Studies reporting altered synchronization of EEGs in various neuropsychiatric disorders**.

Disorder/dysfunction	Relevant publications
AD/HD	Barry et al. (2002, 2003, 2005)
Alcohol abuse	Georgopoulos et al. (2007), Kamarajan et al. (2005)
Alexithymia	Matsumoto et al. (2006), Symond et al. (2005)
Autism	Grice et al. (2001), Just et al. (2004), Orekhova et al. (2007), Rippon et al. (2007), Welsh et al. (2005), Wickelgren (2005)
Bipolar disorders	O’Donnell et al. (2004)
Dementia	Adamis et al. (2005), Adler et al. (2003), Brunovsky et al. (2003), Georgopoulos et al. (2007), Koenig et al. (2005), Leuchter et al. (1987), Prichep et al. (2006), Stam et al. (2003)
Depression	Armitage et al. (1999), Lee et al. (2007), Linkenkaer-Hansen et al. (2005), Llinás et al. (1999), Park et al. (2007)
Hallucinations	Baldeweg et al. (1998)
HIV dementia	Fletcher et al. (1997)
Migraine	Angelini et al. (2004)
Multiple sclerosis	Georgopoulos et al. (2007)
Neuropsychiatric disorders: general reviews	Herrmann and Demiralp (2005), Llinás et al. (1999), Schnitzler and Gross (2005), Stam et al. (2003), Stam (2005), Uhlhaas and Singer (2006)
Parkinson’s disease	Akbari and Gharibzadeh (2009), Levy et al. (2000), Raz et al. (2000), Stoffers et al. (2007)
Post-traumatic stress disorder	Kolassa and Elbert (2007)
Schizophrenia and other psychotic disorders	Basar-Eroglu et al. (2009), Bob (2007), Bob et al. (2008), Breakspear et al. (2004), Brenner et al. (2003), Bressler (2003), Cho et al. (2006), Ferrarelli et al. (2012), Ford and Mathalon (2005), Ford et al. (2007, 2008), Georgopoulos et al. (2007), Green et al. (1999), Hirano et al. (2008), Koukkou et al. (1995), Kwon et al. (1999), Lee et al. (2003a,b), Lewis et al. (1999, 2005), Light et al. (2006), Pinault (2008), Ramos-Loyo et al. (2009), Rockstroh et al. (2007), Roopun et al. (2008), Siegel et al. (2006), Spencer et al. (2003), Spencer et al. (2004, 2008a,b), Spencer and McCarley (2006), Symond et al. (2005), Tononi and Edelman (2000), Uhlhaas and Singer (2006), Uhlhaas et al. (2008), van der Stelt and Belger (2007), Vierling-Claassen et al. (2008), Whittington (2008), Williams et al. (2008), Winterer et al. (2000)
Traumatic brain injury	Dockree et al. (2004), Hoffman et al. (1995), Kumar et al. (2009a,b), Roche et al. (2004), Slewa-Younan et al. (2002), Thatcher et al. (1999, 2001), Thatcher (2000, 2006)

Importantly, the absence of specificity does not preclude clinical utility of qEEG as an evaluation tool. Non-specific measures are particularly important in longitudinal assessment. The classical example is body temperature. Fever is non-specific, but body temperature is nonetheless a central clinical measure. Nevertheless, an important technical point on reliability must be emphasized here. Temperature would not be an effective longitudinal measure if it varied randomly between 85° and 105°. Stated differently, meaningful longitudinal application requires acceptable test–retest reliability. To date, very few test–retest reliability studies of dynamical measures calculated from EEGs have been published. Thus, systematic test–retest determinations in healthy participants are essential next steps in the construction of a clinically useful qEEG technology.

### Reliability of qEEG measures

The reliability of quantitative EEG measures is crucial to their clinical utility. This has been recognized at least since the pioneering work of Kennard and Schwartzman (1957). Nonetheless, test–retest reliabilities are not typically reported and this deficiency is a significant factor limiting the widespread application of qEEG. EEG reliabilities depend on the electrode site(s), the kind of electrode technology used, the frequency band, sampling frequency, the resolution of the digitizer, the duration of the recording epoch, the behavioral task implemented during the recording, the qEEG measure computed, the procedure used to compute it, and the retest interval. Additionally, there has been no agreement in the literature concerning the choice of statistical measure used to quantify EEG reliability. Measures that have been used in EEG studies include Cornbach alpha (Burgess and Gruzelier, 1993; Lund et al., 1995), rank correlation (Gasser et al., 1985; Van Albada et al., 2007), intraclass correlation coefficient (Fein et al., 1983; Tomarken et al., 1992; Gudmussen et al., 2007; Ambrosius et al., 2008; Gram et al., 2014), Pearson correlation coefficient (Fein et al., 1984; Salinsky et al., 1991; Tomarken et al., 1992; Kondacs and Szabo, 1999), Fisher’s *r* (Pollock et al., 1991), two-way analysis of variance (Sloan and Fenton, 1993), coefficient of variation (Maltez et al., 2004; Fingelkurts et al., 2006), and the coefficient of determination (Fingelkurts et al., 2006). This diversity of statistical analysis has made it impossible to obtain a clear understanding of the reliability of many qEEG measures.

We recommend using the intraclass correlation coefficient to report test–retest reliability. Several considerations have motivated this recommendation including the intraclass correlation coefficient’s immediate relationship to the standard error of measurement and the minimal detectable difference. These variables in turn help inform the identification of the minimal clinically important difference, where it is explicitly recognized that clinical significance is determined only in part by statistical significance (Jaeschke et al., 1989; Crosby et al., 2003).

Important conditions should be met when using the intraclass correlation coefficient in reliability studies. This is not a single measure but a class of measures. Shrout and Fleiss (1979) constructed six intraclass correlation measures and McGraw and Wong (1996) constructed ten. The choice of intraclass correlation coefficient is crucial to the analysis. Depending on the formula used, large differences in the numerical value of the coefficient can be obtained with the same data. Guidelines for the choice of appropriate statistical model for a given study, and hence the choice of the appropriate intraclass correlation coefficient, are given in Müller and Büttner (1994) who included a decision tree to summarize the selection process. As noted by Krebs (1986), it is therefore essential to identify the form of coefficient when reporting results. A specification of the coefficient’s confidence interval is also a critical element in the data report (Stratford, 1989). Identification of the confidence interval is important because the interval must be considered when interpreting the coefficient. For example, Morrow and Jackson (1993) have shown that a high value of the coefficient, which suggests that the measure is reliable, can have a large confidence interval, which argues against this interpretation. Donner and Wells (1986) provide a comparison of procedures for constructing confidence intervals for the coefficient. We suggest the Wald method that was used by Zou (2012) to construct an algorithm for estimating the sample size required in studies that use the intraclass correlation coefficient as the statistical measure of reliability.

Two additional issues should be considered in reliability studies. First, reliability is population dependent. A measure that presents adequate reliability in one population may fail to do so in a different population. Examples are found in a study of EEG reliability (Lund et al., 1995) comparing healthy controls and schizophrenics and in a study by Fein et al. (1983) that compared EEG reliabilities obtained with healthy controls and dyslexic participants. Population-dependent determination of reliability is particularly important in the investigation of traumatic brain injury because it has long been recognized that high variability is a characteristic of an injured CNS (Head, 1926; Bleiberg et al., 1997). Second, distributions presented by EEG measures can be markedly non-normal (Gasser et al., 1982). Non-normal distributions can reduce test–retest reliability (Dunlap et al., 1994). Van Albada and Robinson (2007) have shown that a transformation to a near normal distribution can improve EEG test–retest reliability.

## Conclusion and Recommendations

### Electrophysiological assessment and mTBI detection

Based on our critical evaluations of qEEG as an mTBI assessment tool, the following general conclusions are made:

1. qEEG provides, at best, an imperfect assessment of mTBI. The published literature does indicate, however, that it can be an important complement to other assessment procedures.
2. TBI is not a discrete clinical entity. Different injury events can initiate very different pathophysiological processes. It follows that, just as there is not a single test for cancer, there is not a single test for TBI.
3. Reports of high specificity of qEEG evaluations of TBI must be interpreted with care. High specificities have been reported in carefully constructed clinical studies in which healthy control participants were compared against a carefully selected TBI population. The published literature indicates, however, that similar abnormalities in qEEG measures are observed in other neuropsychiatric disorders. While it may be possible to distinguish a clinical patient form a healthy control with this technology, these measures are unlikely to discriminate between, for example, major depressive disorder, bipolar disorder, or TBI. The specificities observed in these clinical studies may well be lost in real world clinical practice.
4. The absence of specificity does not preclude clinical utility. Efficacy as a longitudinal clinical measure does, however, require acceptable test–retest reliability. To date, very few test–retest reliability studies have been published with qEEG data obtained from TBI patients. This is a particular concern because high variability is a known characteristic of the injured CNS.
5. Specific measures and analysis methods were qualitatively assessed based on the degree of testing in mTBI patients, the usefulness for mTBI detection as a unique entity, and the usefulness as a component in a discriminant function for mTBI detection. Table 9 provides a summary of our findings. For example, spectral analysis has been tested extensively in mTBI patients, but its usefulness as a mTBI detection tool (in isolation) is low. The usefulness of spectral analysis as a component in a discriminant function, however, is more promising. Note that some methods require additional investigation before qualitative scores of usefulness can be assigned, but the TWG considers the methods to hold considerable promise.

**Table 9:** **Summary of TWG conclusions on electrophysiological measures for mTBI detection (0 < 1 < 2 < 3 < 4)**.

Measure/analysis method	History of testing in mTBI patients	Usefulness for mTBI detection as a unique entity	Usefulness for mTBI detection as part of a discriminant function
Spectral analysis	4	0	2
Connectivity measures	3	1	3
ERPs	4	2	4
Information dynamics	0	Unknown^a^	Unknown^a^
Symbolic dynamics	2	3	Unknown^a^
Analysis of casual relationships	2	2	Unknown^a^
Graph theory	1	3	Unknown^a^

*^a^Unknown, but high perceived potential*.

### Recommendations for research and continued advanced development

The qEEG TWG recommends the following actions to best advance qEEG and other electrophysiological assessment methods as mTBI detection tools:

1. Reliability in a healthy population is a necessary, but not sufficient, property of a useful clinical measure. Systematic test–retest reliability studies of qEEG measures should be conducted as the first step in the rigorous development of a clinical qEEG technology. Reliability must be established for each candidate measure.
2. We define the clinical validity of a measure as its ability to discriminate between a clinical population and healthy controls and, ideally, its ability to discriminate between different clinical populations. For any given measure, systematic validity studies are required once reliability has been established as outlined in the first recommendation. In the first instance, these studies should compare results obtained with well characterized TBI patients and healthy controls. If the results of these studies are positive, they can be expanded to include other psychiatric populations.
3. Evidence indicates that ERPs can identify abnormalities in cases where EEGs alone are non-disclosing. It should be noted that the same signal amplifiers are used for EEGs and ERPs. Thus, there is no technical barrier to incorporating ERP measurements in an EEG evaluation, as long as careful considerations are made to ensure precise time-locking between stimulus and recording instrumentation. Reliability and validity evaluations must also be performed with ERP-derived measures.
4. The mathematical methods used in signal analysis in other application areas have expanded enormously during the past 30 years. Important growth areas include non-linear dynamical analysis, complexity measures, analysis of causal interactions, graph theory, and information dynamics. These methods have received limited application in the analysis of EEG records obtained from TBI patients. It seems possible that, as was the case in other research domains, the systematic application of these methods in the analysis of mTBI will be similarly useful. The standard mathematical procedures used in the characterization of mTBI EEG abnormalities should be expanded to incorporate these methods of analysis.
5. Reliability and validity study data should, following de-identification, be made available on a publically accessible website for download and reanalysis by other investigators. As this is standard practice in genomics research and astronomy, it should also be standard practice in neuropsychiatry. This recommendation is motivated in part by the rapid advances being made in signal analysis. Public access to well documented EEG records will make it possible for investigators throughout the world to test the reliability and validity of new mathematical measures as they are developed.
6. As argued in the second conclusion, the diversity of underlying neuropathology in the TBI population precludes the development of a single test for TBI. As a potential solution, an integrated neuropsychiatric assessment platform can be developed that will permit simultaneous acquisition of EEG records including ERPs, heart rate variability data, and eye tracking records during a neuropsychological assessment. This system should include an onboard signal analysis capability and be capable of incorporating additional data types including genomic, epigenetic, proteomic/biomarker, and imaging data. The result would be a comprehensive, statistically valid characterization of the CNS.

## Conflict of Interest Statement

The authors declare that the research was conducted in the absence of any commercial or financial relationships that could be construed as a potential conflict of interest.

## Supplementary Material

The Supplementary Material for this article can be found online at http://www.frontiersin.org/Journal/10.3389/fnhum.2015.00011/abstract

## References

[B1] Abásolo D. Hornero R. Espino P. Alvarez D. Poza J. (2006). Entropy analysis of the EEG background activity in Alzheimer’s disease patients. Physiol. Meas. 27, 241–253. 10.1088/0967-3334/27/3/003 16462011

[B2] Abásolo D. Hornero R. Espino P. Poza J. Sánchez C. I. de la Rosa R. (2005). Analysis of regularity in the EEG background activity of Alzheimer’s disease patients with approximate entropy. Clin. Neurophysiol. 116, 1826–1834. 10.1016/j.clinph.2005.04.001 15979403

[B3] Adamis D. Sahu S. Treloar A. (2005). The utility of EEG in dementia: a clinical perspective. Int. J. Geriatr. Psychiatry 20, 1038–1045. 10.1002/gps.1393 16250070

[B4] Adler G. Brassen S. Jajcevic A. (2003). EEG coherence in Alzheimer’s dementia. J. Neural Transm. 110, 1051–1058 10.1007/s00702-003-0024-8 12928837

[B5] Akbari A. Gharibzadeh S. (2009). Beta oscillations as the cause of both hyper- and hypokinetic symptoms of movement disorders. J. Neuropsychiatry Clin. Neurosci. 21, 352 10.1176/jnp.2009.21.3.352 19776327

[B6] Albo Z. Di Prisco G. V. Chen Y. Rangarajan G. Truccolo W. Feng J. (2004). Is partial coherence a viable technique for identifying generators of neural oscillations? Biol. Cybern. 90, 318–326 10.1007/s00422-004-0475-5 15221392

[B7] Ambrosius U. Lietzenmaier S. Wehrle R. Wichniak A. Kalus S. Winkelmann J. (2008). Hereditability of sleep electroencephalogram. Biol. Psychiatry 64, 344–348 10.1016/j.biopsych.2008.03.002 18405882

[B8] Angelini L. de Tommaso M. Guido M. Hu K. Ivanov PCH. Marinazzo D. (2004). Steady state visual evoked potentials and phase synchronization in migraine patients. Phys. Rev. Lett. 93, 038103. 10.1103/PhysRevLett.93.038103 15323876

[B9] Arciniegas D. B. (2011). Cholinergic dysfunction and cognitive impairment after traumatic brain injury. Part 2: evidence from basic and clinical investigations. J. Head Trauma Rehabil. 26, 319–323 10.1097/HTR.0b013e31821ebfb3 21734513

[B10] Arciniegas D. B. Anderson C. A. Topkoff J. Mcallister T. W. (2005). Mild traumatic brain injury: a neuropsychiatric approach to diagnosis, evaluation, and treatment. Neuropsychiatr. Dis. Treat. 1, 311–327. 18568112 PMC2424119

[B11] Arciniegas D. B. Topkoff J. L. (2004). Applications of the P50 evoked response to the evaluation of cognitive impairments after traumatic brain injury. Phys. Med. Rehabil. Clin. N. Am. 15, 177–203. 10.1016/S1047-9651(03)00104-9 15029905

[B12] Armitage R. Hoffman R. F. Rush A. J. (1999). Biological rhythm disturbance in depression. Psychol. Med. 29, 1435–1448. 10.1017/S0033291799001300 10616950

[B13] Arnhold J. Grassberger P. Lehnertz K. Elger C. E. (1999). A robust method for detecting interdependences: application to intracranially recorded EEG. Physica D 134, 419–430 10.1016/S0167-2789(99)00140-2

[B14] Aviyente S. Bernat E. M. Evans W. S. Sponheim S. R. (2011). A phase synchrony measure for quantifying functional integration in the brain. Hum. Brain Mapp. 32, 80–93. 10.1002/hbm.21000 20336687 PMC6870375

[B15] Baccalá L. A. Sameshima K. (2001). Overcoming the limitations of correlation analysis for many simultaneously processed neural structures. Prog. Brain Res. 130, 33–47 10.1016/S0079-6123(01)30004-3 11480285

[B16] Baguley I. J. Felmingham K. L. Lahz S. Gordon E. Lazzaro I. Schotte D. E. (1997). Alcohol abuse and traumatic brain injury: effect on event related potentials. Arch. Phys. Med. Rehabil. 78, 1248–1253. 10.1016/S0003-9993(97)90339-7 9365356

[B17] Baldeweg T. Spence S. Hirsch S. R. Bruzelier J. (1998). Gamma-band electroencephalographic oscillations in a patient with somatic hallucinations. Lancet 352, 620–621 10.1016/S0140-6736(05)79575-1 9746027

[B18] Barr W. B. Prichep L. S. Chabot R. Powell M. R. McCrea M. (2012). Measuring brain electrical activity to track recovery from sport-related concussion. Brain Inj. 26, 58–66. 10.3109/02699052.2011.608216 22107157

[B19] Barry R. J. Clarke A. R. Johnstone S. J. (2003). A review of electrophysiology in attention-deficit/hyperactivity disorder: I. Qualitative and quantitative electroencephalography. Clin. Neurophysiol. 114, 171–183. 10.1016/S1388-2457(02)00362-0 12559224

[B20] Barry R. J. Clarke A. R. McCarthy R. Selikowitz M. (2002). EEG coherence in attention-deficit/hyperactivity disorder: a comparative study of two DSM-IV types. Clin. Neurophysiol. 113, 579–585. 10.1016/S1388-2457(02)00036-6 11956003

[B21] Barry R. J. Clarke A. R. McCarthy R. Selikowitz M. Johnstone S. J. Hsu C.-I. (2005). Age and gender effects in EEG coherence: II. Boys with attention deficit/hyperactivity disorder. Clin. Neurophysiol. 116, 977–984. 10.1016/j.clinph.2004.10.002 15792908

[B22] Bartolomei F. Bosma I. Klein M. Baayen J. C. Reijneveld J. C. Postma T. J. (2006). Distributed functional connectivity in brain tumour patients: evaluation by graph analysis and synchronization matrices. Clin. Neurophysiol. 117, 2039–2049. 10.1016/j.clinph.2006.05.018 16859985

[B23] Basar-Eroglu C. Schmidt-Fehr C. Mathes B. Zimmermann J. Brand A. (2009). Are oscillatory brain responses generally reduced in schizophrenia during long sustained attentional processing? Int. J. Psychophysiol. 71, 75–83. 10.1016/j.ijpsycho.2008.07.004 18725254

[B24] Belmonte M. K. Allen G. Beckel-Mitchner A. Boulanger L. M. Carper R. A. Webb S. H. (2004). Autism and abnormal development of brain connectivity. J. Neurosci. 24, 9228–9231 10.1523/JNEUROSCI.3340-04.2004 15496656 PMC6730085

[B25] Bennouna M. Greene V. B. Defranoux L. (2007). Cholinergic hypothesis in psychosis following traumatic brain injury and cholinergic hypothesis in schizophrenia: a link? Encephale 34(4 Pt 1), 616–620. 10.1016/S0013-7006(07)92062-X 18033152

[B26] Bernhardt B. C. Chen Z. He Y. Evans A. C. Bernasconi N. (2011). Graph-theoretical analysis reveals disrupted small-world organization of cortical thickness correlation networks in temporal lobe epilepsy. Cereb. Cortex 21, 2147–2157. 10.1093/cercor/bhq291 21330467

[B27] Berridge M. J. Rapp P. E. (1979). A comparative survey of the function, mechanism and control of cellular oscillators. J. Exp. Biol. 81, 217–279. 390080 10.1242/jeb.81.1.217

[B28] Bialonski S. Wendler M. Lehnertz K. (2011). Unraveling spurious properties of interaction networks with tailored random networks. PLoS ONE 6:e22826. 10.1371/journal.pone.0022826 21850239 PMC3151270

[B29] Bigler E. D. Maxwell W. L. (2011). Neuroimaging and neuropathology of TBI. Neurorehabilitation 28, 63–74. 10.3233/NRE-2011-0633 21447905

[B30] Bleiberg J. Garmoe W. S. Halpern E. L. Reeves D. L. Nadler J. D. (1997). Consistency of within-day and across-day performance after mild brain injury. Neuropsychiatry Neuropsychol. Behav. Neurol. 10, 247–253. 9359122

[B31] Blinowska K. J. Kaminski M. (2013). Functional brain networks: random, “small world” or deterministic? PLoS ONE 8:e78763. 10.1371/journal.pone.0078763 24205313 PMC3813572

[B32] Bob P. (2007). Consciousness and co-consciousness, binding problem and schizophrenia. Neuro Endocrinol. Lett. 28, 723–726. 18063944

[B33] Bob P. Palus M. Susta M. Glaslova K. (2008). EEG phase synchronization in patients with paranoid schizophrenia. Neurosci. Lett. 447, 73–77. 10.1016/j.neulet.2008.09.055 18835328

[B34] Bonanni E. Borghetti K. D. Fabbrini M. Maestri M. Cignoni F. Sartucci F. (2006). Quantitative EEG analysis in post-traumatic anosmia. Brain Res. Bull. 71, 69–75. 10.1016/j.brainresbull.2006.08.004 17113930

[B35] Bonita J. D. Ambolode L. C. Rosenberg B. M. Cellucci C. J. Watanabe T. A. Rapp P. E. (2014). Time domain measures of inter-channel EEG correlations: a comparison of linear, nonparametric and nonlinear measures. Cogn. Neurodyn. 8, 1–15. 10.1007/s11571-013-9267-8 24465281 PMC3890093

[B36] Bosl W. Tierney A. Tager-Flusberg H. Nelson C. (2011). Response: infant EEG activity as a biomarker for autism: a promising approach or a false promise? BMC Med. 20:60 10.1186/1741-7015-9-60 21599951 PMC3128840

[B37] Bosma I. Douw L. Bartolomei F. Heimans J. J. van Dijk B. W. Postma J. (2008). Synchronized brain activity and neurocognitive function in patients with low grade glioma: a magnetoencephalography study. Neuro Oncol. 10, 734–744. 10.1215/15228517-2008-034 18650489 PMC2666250

[B38] Breakspear M. Brammer M. J. Bullmore E. T. Das P. Williams L. M. (2004). Spatiotemporal wavelet resampling for functional neuroimaging data. Hum. Brain Mapp. 23, 1–25. 10.1002/hbm.20045 15281138 PMC6871944

[B39] Breakspear M. Williams L. M. Stam C. J. (2004). A novel method for the topographic analysis of neural activity reveals the formation and dissolution of ‘dynamic cell assmeblies’. J. Comput. Neurosci. 16, 49–68. 10.1023/B:JCNS.0000004841.66897.7d 14707544

[B40] Breitung J. Swanson N. R. (2002). Temporal aggregation and spurious instantaneous causality in multiple time series models. J. Time Ser. Anal. 23, 651–665 10.1111/1467-9892.00284

[B41] Brenner C. A. Sporns O. Lysaker P. H. O’Donnell B. F. (2003). EEG synchronization to modulated auditory tones in schizophrenia, schizoaffective disorder, and schizotypal personality disorder. Am. J. Psychiatry 160, 2238–2240. 10.1176/appi.ajp.160.12.2238 14638599

[B42] Bressler S. L. (2003). Cortical coordination dynamics and the disorganization syndrome in schizophrenia. Neuropsychopharmacology 28, 535–539. 12827142 10.1038/sj.npp.1300145

[B43] Broglio S. P. Pontifex M. B. O’Connor P. Hillman C. H. (2009). The persistent effects of concussion on neuroelectric indices of attention. J. Neurotrauma 26, 1463–1470. 10.1089/neu.2008-0766 19331519

[B44] Bruhn J. Röpcke H. Rehberg B. Bouillon T. Hoeft A. (2000). Electroencephalogram approximate entropy correctly classifies the occurrence of burst suppression pattern as increasing anesthetic drug effect. Anesthesiology 93, 981–985. 10.1097/00000542-200010000-00018 11020750

[B45] Brunovsky M. Matousek M. Edman A. Cerrena K. Krajea V. (2003). Objective assessment of the degree of dementia by means of EEG. Neuropsychobiology 48, 19–26. 10.1159/000071824 12886036

[B46] Burgess A. Gruzelier J. (1993). Individual reliability of amplitude distribution in topographical mapping of EEG. Electroencephalogr. Clin. Neurophysiol. 86, 219–223. 10.1016/0013-4694(93)90101-Z 7682923

[B47] Campbell K. Houle S. Lorrain D. Deacon-Elliott D. Proulx G. B. (1986). Event-related potentials as an index of cognitive functioning in head-injured patients. Electroencephalogr. Clin. Neurophysiol. 38(Suppl.), 486–488.2035948

[B48] Campbell K. B. DeLugt D. R. (1995). “Event related potential measures of cognitive deficits following closed head injury,” in Event Related Potentials and Cognition: Handbook of Neuropsychology, Vol. 10, ed. Johnson R. Jr. (New York, NY: Elsevier), 269–297.

[B49] Campbell K. B. Suffield J. B. Deacon D. L. (1990). Electrophysiological assessment of cognitive disorder in closed head-injured outpatients. Electroencephalogr. Clin. Neurophysiol. Suppl. 41, 202–215. 10.1016/B978-0-444-81352-7.50025-X 2289430

[B50] Canolty R. T. Knight R. T. (2010). The functional role of cross-frequency coupling. Trends Cogn. Sci. (Regul. Ed.) 14, 506–515 10.1016/j.tics.2010.09.001 20932795 PMC3359652

[B51] Cao C. Slobounov S. (2010). Alteration of cortical functional connectivity as a result of traumatic brain injury revealed by graph theory, ICA and sLORETA analyses of EEG signals. IEEE Trans. Neural. Syst. Rehabil. Eng. 18, 11–19. 10.1109/TNSRE.2009.2027704 20064767 PMC2945220

[B52] Castellanos N. P. Bajo R. Cuesta P. Villacorta-Atienza J. A. Paúl N. Garcia-Prieto J. (2011a). Alteration and reorganization of functional networks: a new perspective in brain injury study. Front. Hum. Neurosci. 5:90. 10.3389/fnhum.2011.00090 21960965 PMC3177176

[B53] Castellanos N. P. Leyva I. Buldú J. M. Bajo R. Paúl N. Cuesta P. (2011b). Principles of recovery from traumatic brain injury: reorganization of functional networks. Neuroimage 55, 1189–1199. 10.1016/j.neuroimage.2010.12.046 21195199

[B54] Castellanos N. P. Paúl N. Ordóñez V. E. Demuynck O. Bajo R. Campo P. (2010). Reorganization of functional connectivity as a correlate of cognitive recovery in acquired brain injury. Brain 133, 2365–2381. 10.1093/brain/awq174 20826433

[B55] Catani C. (2003). Affective Stimulus Processing Following Traumatic Brain Injury. Doctoral Dissertation, Universität Konstanz, Konstanz.

[B56] Catarino A. Churches O. Baron-Cohen S. Andrade A. Ring H. (2011). Atypical EEG complexity in autism spectrum conditions: a multiscale entropy analysis. Clin. Neurophysiol. 122, 2375–2383. 10.1016/j.clinph.2011.05.004 21641861

[B57] Cellucci C. J. Albano A. M. Rapp P. E. (2006). Statistical validation of mutual information calculations: comparisons of alternative numerical algorithms. Phys. Rev. E Stat. Nonlin. Soft Matter Phys. 71(6 Pt 2), 066208. 10.1103/PhysRevE.71.066208 16089850

[B58] Chen X. P. Tao L. Y. Chen A. C. (2006a). Electroencephalogram and evoked potential parameters examined in Chinese mild head injury patients for forensic medicine. Neurosci. Bull. 22, 165–170. 17704845

[B59] Chen Y. Bressler S. L. Ding M. (2006b). Frequency decomposition of conditional Granger causality and application to multivariate neural field potential data. J. Neurosci. Methods 150, 228–237. 10.1016/j.jneumeth.2005.06.011 16099512

[B60] Cho R. Y. Konecky R. O. Carter C. S. (2006). Impairments in frontal cortical gamma synchrony and cognitive control in schizophrenia. Proc. Natl. Acad. Sci. U.S.A. 103, 19878–19883. 10.1073/pnas.0609440103 17170134 PMC1750867

[B61] Chung C. C. Kang J. H. Yuan R. Y. Wu D. Chen C. C. Chi N. F. (2013). Multiscale entropy analysis of electroencephalography during sleep in patients with Parkinson disease. Clin. EEG Neurosci. 44, 221–226. 10.1177/1550059412475066 23545244

[B62] Clark C. R. O’Hanlon A. P. Wright M. J. Geffen G. M. (1992). Event-related potential measurements of deficits in information processing following moderate to severe closed head injury. Brain Inj. 6, 509–520. 10.3109/02699059209008148 1393185

[B63] Costa M. Goldberger A. L. Peng C.-K. (2002). Multiscale entropy analysis of complex physiologic time series. Phys. Rev. Lett. 89, 068102. 10.1103/PhysRevLett.89.068102 12190613

[B64] Costa M. Goldberger A. L. Peng C.-K. (2004). Costa, Goldberger and Peng reply. Phys. Rev. Lett. 92, 089804 10.1103/PhysRevLett.92.089804

[B65] Costa M. J. Goldberger A. L. Peng C.-K. (2005). Multiscale entropy of biological signals. Phys. Rev. E Stat. Nonlin. Soft Matter Phys. 71, 021906 10.1103/PhysRevE.71.021906 15783351

[B66] Coutin-Churchman B. Anez Y. Uzcategui M. Alvarez L. Vergara F. Mendez L. O. (2003). Quantitative spectral analysis of EEG in psychiatry revisited: drawing signs out of numbers in a clinical setting. Clin. Neurophysiol. 114, 2294–2306. 10.1016/S1388-2457(03)00228-1 14652089

[B67] Cremona-Meteyard S. I. Geffen G. M. (1994). Event related potential indices of visual attention following moderate to severe closed head injury. Brain Inj. 8, 541–558. 10.3109/02699059409151006 7987290

[B68] Crosby R. D. Kolotkin R. L. Williams G. R. (2003). Defining clinically meaningful change in quality of life. J. Clin. Epidemiol. 56, 395–407 10.1016/S0895-4356(03)00044-1 12812812

[B69] Crutchfield J. P. Packard N. H. (1983). Symbolic dynamics of noisy chaos. Physica 7D, 201–223.

[B70] Curry S. H. (1980). Event-related brain potentials as indicants of structural and functional damage in closed head injury. Prog. Bain Res. 54, 507–515 10.1016/S0079-6123(08)61668-4 7220959

[B71] Curry S. H. Woods D. L. Low M. D. (1986). Applications of cognitive ERPs in neurosurgical and neurological patients. Electroencephalogr. Clin. Neurophysiol. Suppl. 38, 469–484. 3466781

[B72] D’Alessandro G. Politi A. (1990). Hierarchical approach to complexity with applications to dynamic systems. Phys. Rev. Lett. 64, 1609–1612. 10.1007/s10916-012-9920-5 10041441

[B73] De Beaumont L. Brisson B. Lassonde M. Jolicoeur P. (2007a). Long-term electrophysiological changes in athletes with a history of multiple concussions. Brain Inj. 21, 631–644. 10.1080/02699050701426931 17577714

[B74] De Beaumont L. Lassonde M. Leclerc S. Théoret H. (2007b). Long-term and cumulative effects of sports concussion on motor inhibition. Neurosurgery 61, 329–336. 10.1227/01.NEU.0000280000.03578.B6 17762745

[B75] De Beaumont L. Théoret H. Mongeon D. Messier J. Leclerc S. Tremblay S. (2009). Brain function decline in healthy retired athletes who sustained their last concussion in early adulthood. Brain 132, 695–708. 10.1093/brain/awn347 19176544

[B76] Deacon D. Breton F. Ritter W. Vaughan H. G. Jr. (1991). The relationship between N2 and N400: scalp distribution, stimulus probability, and task relevance. Psychophysiology 28, 185–200. 10.1111/j.1469-8986.1991.tb00411.x 1946885

[B77] Deacon-Elliott D. L. Campbell K. B. (1987). P3 evoked by visual feedback in normals and closed head-injured subjects. Electroencephalogr. Clin. Neurophysiol. Suppl. 40, 664–669. 3480191

[B78] Deacon-Elliott D. Campbell K. B. Suffield J. B. Proulx G. B. (1987). Electrophysiological monitoring of closed head injury: III. Cognitive evoked potentials. Cogn. Rehabil. 5, 12–21.

[B79] Deco G. Schürmann B. (2001). Information Dynamics: Foundations and Applications. New York, NY: Springer-Verlag.

[B80] DeKosky S. T. Ikonomovic M. D. Gandy S. (2010). Traumatic brain injury – football, warfare and long-term effects. N. Engl. J. Med. 363, 1293–1296 10.1056/NEJMp1007051 20879875

[B81] di Russo F. Spinelli D. (2010). Sport is not always healthy: executive brain dysfunction in professional boxers. Psychophysiology 47, 425–434. 10.1111/j.1469-8986.2009.00950.x 20030763

[B82] Dockree P. M. Kelly S. P. Roche R. A. R. Hogan M. J. Reilly R. B. Robertson I. H. (2004). Behavioral and physiological impairments of sustained attention after traumatic brain injury. Brain Res. Cogn. Brain Res. 20, 403–414. 10.1016/j.cogbrainres.2004.03.019 15268918

[B83] Donner A. Wells G. (1986). A comparison of confidence interval methods for the intraclass correlation coefficient. Biometrics 42, 401–412. 10.2307/2531060 3741978

[B84] Douw L. van Dellen E. Baayen J. C. Klein M. Velis D. N. Alpherts W. C. J. (2010). The lesioned brain: still a small world? Front. Hum. Neurosci. 4:174. 10.3389/fnhum.2010.00174 21120140 PMC2991225

[B85] Drake M. John K. (1987). Long-latency auditory evoked potentials in the postconcussive syndrome. Electroencephalogr. Clin. Neurophysiol. 5, 19–21.

[B86] Dressler O. Schneider G. Stockmanns G. Kochs E. F. (2004). Awareness and the EEG power spectrum: analysis of frequencies. Br. J. Anaesth. 93, 806–809. 10.1093/bja/aeh270 15377585

[B87] Duncan C. C. Kosmidis M. H. Mirsky A. F. (2003). Event-related potential assessment of information processing after closed head injury. Psychophysiology 40, 45–59. 10.1111/1469-8986.00006 12751803

[B88] Duncan C. C. Kosmidis M. H. Mirsky A. F. (2005). Closed head injury-related information processing deficits: an event related potential analysis. Int J. Psychophysiol. 58, 133–157 10.1016/j.ijpsycho.2005.05.011 16203052

[B89] Dunlap W. P. Chen R. S. Greer T. (1994). Skew reduces test-retest reliability. J. Appl. Psychol. 79, 310–313 10.1037/0021-9010.79.2.310

[B90] Dupuis F. Johnston K. M. Lavoie M. Lepore F. Lassonde M. (2000). Concussions in athletes produce brain dysfunction as revealed by event-related potentials. Neuroreport 11, 4087–4092. 10.1097/00001756-200012180-00035 11192633

[B91] Dywan M. J. Segalowitz S. J. (1996). Self- and family ratings of adaptive behavior after traumatic brain injury: psychometric scores and frontally generated ERPs. J. Head Trauma Rehabil. 11, 79–95 10.1097/00001199-199604000-00008

[B92] Ebeling W. (1997). Prediction and entropy of nonlinear dynamical systems and symbolic sequences with LRO. Physica D 109, 42–52 10.1016/S0167-2789(97)00157-7

[B93] Eckmann J. P. Ruelle D. (1985). Ergodic theory of chaos and strange attractors. Rev. Mod. Phys. 57, 617–656. 10.1103/RevModPhys.57.61712780039

[B94] Elting J. W. Maurits N. van Weerden T. Spikman J. de Keyser J. van der Naalt J. (2008). P300 analysis techniques in cognitive impairment after brain injury: comparison with neuropsychological and imaging data. Brain Inj. 22, 870–881. 10.1080/02699050802403581 18850345

[B95] Fan Y. Ji M. Zang L. Wang W. Yin Q. Xu J. (2011). Comparison of epidural tramadol-ropivacaine and fentanyl-ropivacaine for labor analgesia: a prospective randomized study. Ups. J. Med. Sci. 116, 252–257. 10.3109/03009734.2011.601532 22066973 PMC3207300

[B96] Fazel S. Wolf A. Pillas D. Lichtenstein P. Längström N. (2014). Suicide, fatal injuries and other causes of premature mortality in patients with traumatic brain injury. JAMA Psychiatry 71, 326–333. 10.1001/jamapsychiatry.2013.3935 24430827 PMC4058552

[B97] Fein G. Galin D. Johnstone J. Yingling C. D. Marcus M. Kiersch M. F. (1983). EEG power spectra in normal and dyslexic children: reliability during passive conditions. Electroencephalogr. Clin. Neurophysiol. 55, 399–405 10.1016/0013-4694(83)90127-X 6187532

[B98] Fein G. Galin D. Yingling C. Johnstone J. (1984). EEG spectra in 10-12 year old boys are stable over 1 to 3 years. Electroencephalogr. Clin. Neurophysiol. 58, 517–518 10.1016/0013-4694(84)90041-5 6209101

[B99] Ferrarelli F. Sarasso S. Guller Y. Riedner B. A. Peterson M. J. Bellesi M. (2012). Reduced natural oscillatory frequency of frontal thalamocortical circuits in schizophrenia. Arch. Gen. Psychiatry 69, 766–774. 22474071 10.1001/archgenpsychiatry.2012.147 PMC3394893

[B100] Fingelkurts A. A. Fingelkurts A. A. Ermolaev V. A. Kaplan A. Y. (2006). Stability, reliability and consistency of the compositions of brain oscillations. Int. J. Psychophysiol. 59, 116–126. 10.1016/j.ijpsycho.2005.03.014 15946755

[B101] Fletcher D. J. Raz J. Fein G. (1997). Intra-hemispheric alpha coherence decreases with increasing cognitive impairment in HIV patients. Electroencephalogr. Clin. Neurophysiol. 102, 286–294. 10.1016/S0013-4694(96)96071-X 9146488

[B102] Folmer R. L. Billings C. J. Diedesch-Rouse A. C. Gallun F. J. Lew H. L. (2011). Electrophysiolgoical assessments of cognitive and sensory processing in TBI: applications for diagnosis, prognosis and rehabilitation. Int. J. Psychophysiol. 82, 4–15. 10.1016/j.ijpsycho.2011.03.005 21419179

[B103] Ford J. M. Krystal J. H. Mathalon D. H. (2007). Neural synchrony in schizophrenia: from networks to new treatments. Schizophr. Bull. 33, 848–852. 10.1093/schbul/sbm062 17567628 PMC2632315

[B104] Ford J. M. Mathalon D. H. (2005). Corrollary discharge dysfunction in schizophrenia: can it explain auditory hallucinations? Int. J. Psychophysiol. 58, 179–189. 10.1016/j.ijpsycho.2005.01.014 16137779

[B105] Ford J. M. Roach B. J. Faustman W. O. Mathalon D. H. (2008). Out-of-synch and out-of-sorts: dysfunction of motor-sensory communication in schizophrenia. Biol. Psychiatry 63, 736–743. 10.1016/j.biopsych.2007.09.013 17981264 PMC2330266

[B106] Ford M. R. Khalil M. (1996a). Evoked potential findings in mild head traumatic head injury. 1. Middle latency component augmentation and cognitive component attenuation. J. Head Trauma Rehabil. 11, 1–15 10.1097/00001199-199606000-00004

[B107] Ford M. R. Khalil M. (1996b). Evoked potential findings in mild traumatic brain injury. 2. Scoring system and individual discrimination. J. Head Truma Rehabil. 11, 16–21 10.1097/00001199-199606000-00005

[B108] Fries P. (2005). A mechanism for cognitive dynamics: neuronal communication through neuronal coherence. Trends Cogn. Sci. (Regul. Ed.) 9, 474–480. 10.1016/j.tics.2005.08.011 16150631

[B109] Gaetz M. Bernstein D. (2001). The current status of electrophysiologic procedures for the assessment of mild traumatic brain injury. J. Head Trauma Rehabil. 16, 386–405. 10.1097/00001199-200108000-00008 11461660

[B110] Gaetz M. Goodman D. Weinberg H. (2000). Electrophysiological evidence for the cumulative effects of concussion. Brain Inj. 14, 1077–1088. 10.1080/02699050050203577 11147580

[B111] Gaetz M. Weinberg H. (2000). Electrophysiological indices of persistent post-concussion symptoms. Brain Inj. 14, 815–832. 10.1080/026990500421921 11030455

[B112] Gallager R. G. (1968). Information Theory and Reliable Communication. New York, NY: John Wiley and Sons.

[B113] Gasser T. Bacher P. Steinberg H. (1985). Test-retest reliability of spectral parameters of the EEG. Electroencephalogr. Clin. Neurophysiol. 60, 312–319. 10.1016/0013-4694(85)90005-7 2579798

[B114] Gasser T. Bucher P. Mochs J. (1982). Transformation towards the normal distribution of broad band spectral parameters of the EEG. Electroencephalogr. Clin. Neurophysiol. 53, 119–124. 10.1016/0013-4694(82)90112-2 6173196

[B115] Geisler M. W. Schlotfeldt C. R. Middleton C. B. Dulay M. F. Murphy C. (1999). Traumatic brain injury assessed with olfactory event-related potentials. J. Clin. Neurophysiol. 16, 77–86. 10.1097/00004691-199901000-00008 10082095

[B116] Georgopoulos A. P. Karageorgiou E. Leuthold A. C. Lewis S. M. Lynch J. K. Alonso A. A. (2007). Synchronous neural interactions assessed by magnetoencephalography: a functional biomarker for brain disorders. J. Neural Eng. 4, 349–355. 10.1088/1741-2560/4/4/001 18057502

[B117] Giaquinto S. (2004). Evoked potentials in rehabilitation. A review. Funct. Neurol. 19, 219–225. 15776789

[B118] Goh S. Y. Irimia A. Togerson C. M. Van Horn J. D. (2014). Neuroinformatics challenges to the structural, connectomic, functional and electrophysiological multimodal imaging of human traumatic brain injury. Front. Neuroinformatics 8:19. 10.3389/fnin.2014.00019 24616696 PMC3935464

[B119] Gosselin N. Bottari C. Chen J. K. Huntgeburth S. C. de Beaumont L. Petrides M. (2012). Evaluating the cognitive consequences of mild traumatic brain injury and concussion by using electrophysiology. Neurosurg. Focus 33, E7:1–7. 10.3171/2012.10.FOCUS12253 23199430

[B120] Gosselin N. Bttari C. Chen J.-K. Petrides M. Tinawi S. de Guise E. (2011). Electrophysiology and functional MRI in post-acute mild traumatic brain injury. J. Neurotrauma 28, 329–341. 10.1089/neu.2010.1493 21309680

[B121] Gosselin N. Lassonde M. Petit D. Leclerc S. Mongrain V. Collie A. (2009). Sleep following sport-related concussions. Sleep Med. 10, 35–46. 10.1016/j.sleep.2007.11.023 18226956

[B122] Gosselin N. Thériault M. Leclerc S. Montplaisir J. Lassonde M. (2006). Neurophysiological anomalies in symptomatic and asymptomatic concussed athletes. Neurosurgery 58, 1151–1161. 10.1227/01.NEU.0000215953.44097.FA 16723894

[B123] Gram M. Graversen C. Olesen S. S. Drewes A. M. (2014). Dynamic spectral indices of the electroencephalogram provide new insights into chronic pain. Clin. Neurophysiol. 10.1016/j.clinph.2014.07.027 25213351

[B124] Granger C. W. J. (1969). Investigating causal relations by econometric models and cross-spectral methods. Econometrica 37, 424–438 10.2307/1912791

[B125] Green M. R. Nuechterlein K. H. Breitmeyer B. Mintz J. (1999). Backward masking in unmedicated schizophrenia patients in psychotic remission: possible reflection of aberrant cortical oscillations. Am. J. Psychiatry 156, 1367–1373. 10484946 10.1176/ajp.156.9.1367

[B126] Grefkes C. Fink G. R. (2012). Disruption of motor network connectivity post-stroke and its noninvasive neuromodulation. Curr. Opin. Neurol. 25, 670–675. 10.1097/WCO.0b013e3283598473 23108249

[B127] Grice S. J. Spratling W. Karmiloff-Smith A. Halit H. Csibra G. de Haan M. (2001). Disordered visual processing and oscillatory brain activity in autism and Williams syndrome. Neuroreport 12, 2697–2700. 10.1097/00001756-200108280-00021 11522950

[B128] Gross J. Schmitz F. Schnitzler I. Kessler K. Shapiro K. Hommel B. (2004). Modulation of long range neuroal synchrony reflects temporal limitations of visual attention in humans. Proc. Natl. Acad. Sci. U.S.A. 101, 13050–13055. 10.1073/pnas.0404944101 15328408 PMC516515

[B129] Gudmussen S. Runarsson T. P. Sigurdsson S. Eiriksdottir G. Johnsen K. (2007). Reliability of quantitative EEG features. Clin. Neuropshysiol. 118, 2162–2171 10.1016/j.clinph.2007.06.018 17765604

[B130] Guevara R. Pérez Velazquez J. L. Nenadovic V. Wennberg R. Senjanović G. Dominguez L. G. (2005). Phase synchronization measurements using electroencephalographic recordings. What can we really say about neuronal synchrony? Neuroinformatics 3, 301–314. 16284413 10.1385/NI:3:4:301

[B131] Güntekin B. Saatci E. Yener G. (2008). Decrease of evoked delta, theta and alpha coherences in Alzheimer patients during a visual oddball task. Brain Res. 1235, 109–116. 10.1016/j.brainres.2008.06.028 18598686

[B132] Haglund Y. Persson H. E. (1990). Does Swedish amateur boxing lead to chronic brain damage. 3. A retrospective clinical neurophysiological study. Acta Neurol. Scand. 82, 353–360. 10.1111/j.1600-0404.1990.tb03316.x 2127153

[B133] Ham T. E. Sharp D. J. (2012). How can investigation of network function inform rehabilitation after traumatic brain injury? Curr. Opin. Neurol. 25, 662–669. 10.1097/WCO.0b013e328359488f 23108248

[B134] Harner R. (2010). Automatic EEG spike detection. Clin. EEG Neurosci. 40, 262–270 10.1177/155005940904000408 19780347

[B135] Haufe S. Nikulin V. V. Müller K. R. Nolte G. (2013). A critical assessment of connectivity measures for EEG data: a simulation study. Neuroimage 64, 120–133. 10.1016/j.neuroimage.2012.09.036 23006806

[B136] Haufe S. Treder M. S. Gugler M. F. Sagebaum M. Curio G. Blankertz B. (2011). EEG potentials predict upcoming emergency brakings during simulated driving. J. Neural Eng. 8, 056001. 10.1088/1741-2560/8/5/056001 21799241

[B137] He Z. Maekawa K. (2001). On spurious Granger causality. Econ. Lett. 73, 307–313 10.1016/S0165-1765(01)00498-0

[B138] Head H. (1926). Aphasia and Kindred Disorders of Speech. Cambridge: Cambridge University Press.

[B139] Heinze H.-J. Münte T. F. Gobiet W. Niemann H. Ruff R. M. (1992). Parallel and serial visual search after closed head injury: electrophysiological evidence for perceptual dysfunctions. Neuropsychologia 30, 495–514. 10.1016/0028-3932(92)90054-P 1641115

[B140] Herrmann C. S. Demiralp T. (2005). Human EEG gamma oscillations in neuropsychiatric disorders. Clin. Neurophysiol. 116, 2719–2733. 10.1016/j.clinph.2005.07.007 16253555

[B141] Hesse W. Möller E. Arnold M. Schack B. (2003). The use of time-variant EEG Granger causality for inspecting directed interdependencies of neural assemblies. J. Neurosci. Methods 124, 27–44. 10.1016/S0165-0270(02)00366-7 12648763

[B142] Hirano S. Hirano Y. Maekawa T. Obayashi C. Oribe N. Kuroki T. (2008). Abnormal neural oscillatory activity to speech sounds in schizophrenia: a magnetoencephalolgraphy study. J. Neurosci. 28, 4897–4903. 10.1523/JNEUROSCI.5031-07.2008 18463243 PMC6670738

[B143] Ho K. K. L. Peng C.-K. Mietus J. E. Larson M. G. Levy D. Goldberger A. L. (1997). Predicting survival in heart failure cases and controls using fully automated methods for deriving nonlinear and conventional indices of heart rate dynamics. Circulation 96, 842–848. 10.1161/01.CIR.96.3.842 9264491

[B144] Hoffman D. A. Lubar J. F. Thatcher R. W. Sterman M. B. Rosenfeld P. J. Striefel S. (1999). Limitations of the American Academy of Neurology and American Clinical Neurophysiology Soociety paper on QEEG. J. Neuropsychiatry Clin. Neurosci. 11, 401–407. 10.1176/jnp.11.3.401 10440020

[B145] Hoffman D. A. Stockdale S. Hicks L. L. Schwaninger B. A. (1995). Diagnosis and treatment of head injury. J. Neurother. 1, 14–21 10.1300/J184v01n01_03

[B146] Hoge C. W. McGurk D. Thomas J. L. Cox A. L. Engel C. C. Castro C. A. (2008). Mild traumatic brain injury in U.S. soldiers returning from Iraq. N. Engl. J. Med. 358, 453–463 10.1056/NEJMoa072972 18234750

[B147] Hu J. Gao J. Principe J. C. (2006). Analysis of biomedical signals by the lempel-Ziv complexity: the effect of finite data size. IEEE Trans. Biomed. Eng. 53(12 Pt 2), 2606–2609. 10.1109/TBME.2006.883825 17152441

[B148] Hurtado J. M. Rubchinskiy L. L. Sigvardt K. A. (2004). Statistical method for detecting of phase locking episodes in neural oscillations. J. Neurophysiol. 91, 1883–1898. 10.1152/jn.00853.2003 15010498

[B149] Inouye T. Shinosaki K. Iyama A. Matsumoto Y. (1993). Localization of activated areas and directional EEG patterns during mental arithmetic. Electroencephalogr. Clin. Neurophysiol. 86, 224–230. 10.1016/0013-4694(93)90102-2 7682924

[B150] Inouye T. Shinosaki K. Yagasaki A. (1983). The direction of spread of alpha activity over the scalp. Electroencephalogr. Clin. Neurophysiol. 55, 290–300. 10.1016/0013-4694(83)90207-9 6186461

[B151] Irimia A. Goh S. Y. M. Torgerson C. M. Chambers M. C. Kikinis R. Van Horn J. D. (2013a). Forward and inverse electroencephalographic modeling in health and in acute traumatic brain injury. Clin. Neurophysiol. 124, 2129–2145. 10.1016/j.clinph.2013.04.336 23746499 PMC3805748

[B152] Irimia A. Goh S.-Y. M. Torgerson C. M. Stein N. R. Chambers M. C. Vespa P. M. (2013b). Electroencephalographic inverse localization of brain activity in acute traumatic brain injury as a guide to surgery, monitoring and treatment. Clin. Neurol. Neurosurg. 115, 2159–2165. 10.1016/j.clineuro.2013.08.003 24011495 PMC3807758

[B153] Irimia A. Swinney K. R. Wikswo J. P. (2009). Partial independence of bioelectric and biomagnetic fields and its implications for electroencephalography and cardiology. Phys. Rev. E Stat. Nonlin. Soft Matter Phys. 79, 051908. 10.1103/PhysRevE.79.051908 19518481 PMC3818693

[B154] Irimia A. Van Horn J. D. Halgren E. (2012). Source cancelation profiles of electroencephalography and magnetoencephalography. Neuroimage 59, 2464–2474. 10.1016/j.neuroimage.2011.08.104 21959078 PMC3254784

[B155] Izhikevich E. M. (2006). Polychronization: computing with spikes. Neural Comput. 15, 1511–1523 10.1162/089976603321891783 12816564

[B156] Jaeschke R. Singer J. Guyatt G. H. (1989). Measurement of health status: ascertaining the minimal clinically important difference. Control. Clin. Trials 10, 407–415. 10.1016/0197-2456(89)90005-6 2691207

[B157] Jiménez-Montanó M. A. (1984). On the syntactic structure of protein sequences and the concept of complexity. Bull. Math. Biol. 46, 641–659 10.1016/S0092-8240(84)80064-6

[B158] Just M. A. Cherkassky V. L. Keller T. A. Minshew J. W. (2004). Cortical activation and synchronization during sentence comprehension in high-functioning autism: evidence of underconnectivity. Brain 127, 1811–1821. 10.1093/brain/awh199 15215213

[B159] Kaipio M. L. Alho K. Winkler I. Escera C. Surma-aho O. Näätänen R. (1999). Event-related brain potentials reveal covert distractibility in closed head injuries. Neuroreport 10, 2125–2129. 10.1097/00001756-199907130-00024 10424686

[B160] Kaipio M. L. Novitski N. Tervaniemi M. Alho K. Ohman J. Salonen O. (2001). Fast vigilance decrement in closed head injury patients as reflected by the mismatch negativiy (MMN). Neuroreport 12, 1517–1522. 10.1097/00001756-200105250-00043 11388440

[B161] Kamarajan C. Porjesz B. Jones K. Chorlian D. Padmanabhapillai A. Rangaswamy M. (2005). Event-related oscillations in offspring of alcoholics: neurocognitive disinhibition as a risk for alcoholism. Biol. Psychiatry 59, 625–634. 10.1016/j.biopsych.2005.08.017 16213472 PMC3766847

[B162] Kaminski J. Brzezicka A. Wrobel A. (2011). Short-term memory capacity (7 ± 2) predicted by theta to gamma cycle length ratio. Neurobiol. Learn. Mem. 95, 19–23. 10.1016/j.nlm.2010.10.001 20951219

[B163] Kana R. K. Keller T. A. Minshew N. Just M. A. (2007). Inhibitory control in high function autism: decreased activation and underconnectivity in inhibition networks. Biol. Psychiatry 62, 198–206. 10.1016/j.biopsych.2006.08.004 17137558 PMC4492460

[B164] Kane N. M. Curry S. H. Rowlands C. A. Manara A. R. Lewis T. Moss T. (1996). Event-related potentials – neurophysiological tools for predicting emergence and early outcome from traumatic coma. Intensive Care Med. 22, 39–46. 10.1007/BF01728329 8857436

[B165] Kaneko K. (1986). Lyapunov analysis and information flow in coupled map lattices. Physica D 23, 436–477 10.1016/0167-2789(86)90149-1

[B166] Kasahara M. Menon D. K. Salmond C. H. Outtrim J. G. Taylor Tavares J. V. Carpenter T. A. (2010). Altered functional connectivity in the motor network after traumatic bain injury. Neurology 75, 168–176 10.1212/WNL.0b013e3181e7ca58 20625170 PMC2905928

[B167] Kennard M. A. Schwartzman A. E. (1957). A longitudinal study of electroencephalographic frequency patterns in mental hospital patients and normal controls. Electroencephalogr. Clin. Neurophysiol. 9, 263–274 10.1016/0013-4694(57)90059-7 13414650

[B168] Keren O. Ben-Dror S. Stern M. J. (1998). Event related potentials as an index of cognitive function during recovery from severe closed head injury. J. Head Trauma Rehabil. 13, 13–30. 9582176 10.1097/00001199-199806000-00003

[B169] Kiebel S. J. Dunizeau J. Friston K. J. (2008). A hierarchy of time scales in the brain. PLoS Comput. Biol. 4:e1000209. 10.1371/journal.pcbi.1000209 19008936 PMC2568860

[B170] Koenig T. Lehmann D. Merlo M. C. Kochi K. Hell D. Koukkou M. (1999). A deviant EEG brain microstate in acute, neuroleptic-naive schizophrenics at rest. Eur. Arch. Psychiatry Clin. Neurosci. 249, 205–211. 10.1007/s004060050088 10449596

[B171] Koenig T. Prichep L. Dierks T. Hubl D. Wahlund L. O. John E. R. (2005). Decreased synchronization in Alzheimer’s disease and mild cognitive impairment. Neurobiol. Aging 26, 165–171. 10.1016/j.neurobiolaging.2004.03.008 15582746

[B172] Kolassa I.-T. Elbert T. (2007). Structural and functional neuroplasticity in relation to traumatic stress. Curr. Dir. Psychol. Sci. 16, 321–325. 10.1016/j.neuroimage.2011.01.024 21255654 PMC3062748

[B173] Kolmogorov A. N. (1958). A metric invariant of transient dynamical systems and automorphisms in Lebsegue spaces. Dokl. Acad. Nauk USSR 119, 861–864.

[B174] Kolmogorov A. N. (1959). Entropy per unit time as a metric invariant of automorphisms. Dokl. Akad. Nauk. SSSR 124, 754–755.

[B175] Kondacs A. Szabo M. (1999). Long-term intra-individual variability of the background EEG in normals. Clin. Neurophysiol. 110, 1708–1716. 10.1016/S1388-2457(99)00122-4 10574286

[B176] Korn A. Golan H. Melamed I. Pascual-Marqui R. Friedman A. (2005). Focal cortical dysfunction and blood-brain barrier disruption in patients with postconcussion syndrome. J. Clin. Neurophysiol. 22, 1–9. 10.1097/01.WNP.0000150973.24324.A7 15689708

[B177] Koukkou M. Lehmann D. Federspiel A. Merlo M. C. (1995). EEG reactivity and EEG activity in never-treated acute schizophrenics, measured with spectral parameters and dimensional complexity. J. Neural Transm. Gen. Sect. 99, 89–102. 10.1007/BF01271472 8579811

[B178] Krebs D. E. (1986). Declare your ICC type. Phys. Ther. 66, 1431. 3749277 10.1093/ptj/66.9.1431

[B179] Kumar R. Gupta R. K. Husain M. Chaudhry C. Srivastava A. Saksena S. (2009a). Comparative evaluation of corpus callosum DTI metrics in acute mild and moderate traumatic brain injury: its correlation with neuropsychometric tests. Brain Inj. 23, 675–685. 10.1080/02699050903014915 19557571

[B180] Kumar S. Rao S. L. Chandramouli A. Pillai S. V. (2009b). Reduction of functional brain connectivity in mild traumatic brain injury during working memory. J. Neurotrauma 26, 665–676. 10.1089/neu.2008-0644 19331523

[B181] Kwon J. S. O’donnell B. F. Wallenstein G. V. Greene R. W. Hirayasu Y. Nestor P. G. (1999). Gamma frequency-range abnormalities to auditory stimulation in schizophrenia. Arch. Gen. Psychiatry 56, 1001–1005. 10565499 10.1001/archpsyc.56.11.1001 PMC2863027

[B182] Lachapelle J. Bolduc-Teasdale J. Ptito A. McKerral M. (2008). Deficits in complex visual information processing after mild TBI: electrophsyiological markers and vocational outcome prognosis. Brain Inj. 22, 265–274. 10.1080/02699050801938983 18297598

[B183] Lachaux J. P. Rodriguez E. Martinerie J. Varela F. J. (1999). Measuring phase synchrony in brain signals. Hum. Brain Mapp. 8, 194–208. 10.1002/(SICI)1097-0193(1999)8:4<194::AID-HBM4>3.0.CO;2-C 10619414 PMC6873296

[B184] Lanius R. A. Wiliamson P. C. Densmore M. Boksman K. Neufeld R. W. Gati J. S. (2004). The nature of traumatic memories: a 4-T fMRI functional connectivity analysis. Am. J. Psychiatry 161, 36–44. 10.1176/appi.ajp.161.1.36 14702248

[B185] Lavoie M. E. Dupuis F. Johnston K. M. Leclerc S. Lassonde M. (2004). Visual P300 effects beyond symptoms in concussed college athletes. J. Clin. Exp. Neuropsychol. 26, 55–73 10.1076/jcen.26.1.55.23936 14972694

[B186] Lawrie S. M. Buechel C. Whalley H. C. Frith C. D. Friston K. J. Johnstone E. C. (2002). Reduced frontotemporal functional connectivity in schizophrenia associated with auditory hallucinations. Biol. Psychiatry 51, 1008–1011. 10.1016/S0006-3223(02)01316-1 12062886

[B187] Le Van Quyen M. Adam C. Baulac M. Martinerie J. Varela F. J. (1998). Nonlinear interdependencies of EEG signals in human intracranially recorded temporal lobe seizures. Brain Res. 792, 24–40. 10.1016/S0006-8993(98)00102-4 9593809

[B188] Le Van Quyen M. Martinerie J. Adam C. Varela F. J. (1999). Nonlinear analyses of interictal EEG map the brain interdependencies in human focal epilepsy. Physica D 127, 250–266 10.1016/S0167-2789(98)00258-9

[B189] Lee J. S. Yang B. H. Lee J. H. Choi J. H. Choi I. G. Kim S. B. (2007). Detrended fluctuation analysis of resting EEG in depressed outpatients and healthy controls. Clin. Neuorphysiol. 118, 2489–2496. 10.1016/j.clinph.2007.08.001 17890151

[B190] Lee K.-H. Williams L. M. Breakspear M. Gordon E. (2003a). Synchronous gamma activity: a review and contribution to an integrative neuroscience model of schizophrenia. Brain Res. Rev. 41, 57–78. 10.1016/S0006-8993(02)03841-6 12505648

[B191] Lee K.-H. Williams L. M. Haig A. Gordon E. (2003b). “Gamma (40 Hz) phase synchronicity” and symptom dimension in schizophrenia. Cogn. Neuropsychiatry 8, 57–71. 10.1080/713752240 16571550

[B192] Lehmann D. Faber P. L. Galderisi S. Herrmann W. M. Kinoshita T. Koukkou M. (2005). EEG microstate duration and syntax in acute, medication-naive, first-episode schizophrenia: a multi-center study. Psychiatry Res. 138, 141–156. 10.1016/j.pscychresns.2004.05.007 15766637

[B193] Leistedt S. J. Coumans N. Dumont M. Lanquart J. P. Stam C. J. Linkowski P. (2009). Altered sleep brain functional connectivity in acutely depressed patients. Hum. Brain Mapp. 30, 2207–2219. 10.1002/hbm.20662 18937282 PMC6870637

[B194] Lempel A. Ziv J. (1976). On the complexity of finite sequences. IEEE Trans. Inf. Theory 22, 75–81 10.1109/TIT.1976.1055501

[B195] Lenne B. Blano J. L. Nandrino J. L. Gallois P. Hautecoeur P. Pezard L. (2012). Decrease of mutual information in bain electrical activity of patients with relapsing-remitting multiple sclerosis. Behav. Neurol. 27, 201–212. 10.3233/BEN-120278 23242355 PMC5214621

[B196] Leon-Carrion J. Martin-Rodriguez J. F. Damas-Lopes J. Barroso Y. Martin J. M. Dominguez-Morales M. R. (2008). A QEEG index of level of functional dependence for people sustaining acquired brain injury: the Seville independence index (SINDI). Brain Inj. 22, 61–74. 10.1080/02699050701824143 18183510

[B197] Leuchter A. F. Spar J. E. Walter D. O. Weiner H. (1987). Electroencephalographic spectra and coherence in the diagnosis of Alzheimer’s-type multi-infarct dementia. A pilot study. Arch. Gen. Psychiatry 44, 993–998. 10.1001/archpsyc.1987.01800230073012 3314770

[B198] Levy R. Hutchison W. D. Lozano A. M. Dostrovsky J. O. (2000). High-frequency synchronization of neuronal activity in the subthalamic nucleus of parkinsonian patients with limb tremor. J. Neurosci. 20, 7766–7775. 11027240 10.1523/JNEUROSCI.20-20-07766.2000 PMC6772896

[B199] Lew H. L. (2001). Combined use of cognitive P300 response and somatosensory evoked potentials (SSEP) in predicting outcomes of severe traumatic brain injury (TBI) patients. Am. J. Phys. Med. Rehabil. 80, 310–311. 10.1097/00002060-200301000-00009 12510186

[B200] Lew H. L. (2005). Rehabilitation needs of an increasing population of patients: traumatic brain injury, polytrauma, and blast related injuries. J. Rehabil. Res. Dev. 42, xiii–xv 10.1682/JRRD.2005.07.0124 16320135

[B201] Lew H. L. Chen C. P. C. Chen M. J. L. Hsu T. H. C. Tang S. F. T. Date E. S. (2002). Comparing the effects of different speech targets on cognitive event-related potentials: theoretical implications for evaluating brain injury. Am. J. Phys. Med. Rehabil. 81, 524–528 10.1097/00002060-200207000-00011 12131180

[B202] Lew H. L. Dikmen S. Slimp J. Temkin N. Lee E. H. Newell D. (2003). The use of somatosensory evoked potentials and cogntive event-related potentials in predicting out-comes of patients with severe traumatic brain injury. Am. J. Phys. Med. Rehabil. 82, 53–61. 10.1097/00002060-200301000-00009 12510186

[B203] Lew H. L. Gray M. Poole J. H. (2007a). Temporal stability of auditory event-related potentials in healthy individuals and patients with traumatic brain injury. J. Clin. Neurophysiol. 24, 392–397. 10.1097/WNP.0b013e31814a56e3 17912063

[B204] Lew H. L. Gray M. Poole J. H. Chow E. Salerno R. M. Date E. S. (2007b). Test-retest reliability of ERPs in TBI and healthy individuals. J. Head Trauma Rehabil. 21, 430 10.1097/00001199-200609000-00050

[B205] Lew H. L. Lee E. H. Pan S. S. L. Date E. S. (2004). Electrophysiological abnormalities of auditory and visual information processing in patients with traumatic brain injury. Am. J. Phys. Med. Rehabil. 83, 428–433. 10.1097/PHM.0b013e3181911102 15166686

[B206] Lew H. L. Poole J. H. Alvarez S. Moore W. (2005a). Soldiers with occult traumatic brain injury. Am. J. Phys. Med. Rehabil. 84, 393–398 10.1097/01.phm.0000163703.91647.a7 15905652

[B207] Lew H. D. Poole J. H. Chiang J. Y. P. Lee E. H. Date E. S. Warden D. (2005b). Event related potential in facial affect recognition: potential clinical utility in patients with traumatic brain injury. J. Rehabil. Res. Dev. 42, 29–34. 10.1682/JRRD.2004.05.0056 15742247

[B208] Lew H. L. Pan S. L. Lee E. H. (2008). “Electrophysiological assessment techniques: evoked potentials and electroencephalography,” in *Brain Injury Medicine: Principles and Practice*, eds Zasler N. D. Katz D. Zafonte R. (New York: Demos Medical Publishing), 157–165.

[B209] Lew H. L. Poole J. H. Castenada A. Salerno R. M. Gray M. (2006). Prognostic value of evoked potentials and event-related potentials in moderate to severe brain injury. J. Head Trauma Rehabil. 21, 350–360. 10.1097/00001199-200607000-00006 16915010

[B210] Lew H. L. Slimp J. Price R. Massagli T. L. Robinson L. R. (1999). Comparison of speech-evoked v. tone-evoked P300 response: implications for predicting outcomes in patients with traumatic brain injury. Am. J. Phys. Med. Rehabil. 78, 367–371. 10.1097/00002060-199907000-00014 10418844

[B211] Lewis D. Pierri J. Volk D. Melchitzky D. Woo T. U. (1999). Altered GABA neurotransmission and prefrontal cortical dysfunction in schizophrenia. Biol. Psychiatry 46, 616–626. 10.1016/S0006-3223(99)00061-X 10472415

[B212] Lewis D. A. Hashimoto T. Volk D. W. (2005). Cortical inhibitory neurons and schizophrenia. Nat. Rev. Neurosci. 6, 312–324. 10.1038/nrn1648 15803162

[B213] Li Y. Tong S. Liu D. Gai Y. Wang X. Wang J. (2008). Abnormal EEG complexity in patients with schizophrenia and depression. Clin. Neurophysiol. 119, 1232–1241. 10.1016/j.clinph.2008.01.104 18396454

[B214] Light G. A. Hsu J. L. Hsich M. H. Meyer-Gomes K. Sprack J. Swerdlow N. R. (2006). Gamma band oscillations reveal neural network cortical coherence dysfunction in schizophrenic patients. Biol. Psychiatry 60, 1231–1240. 10.1016/j.biopsych.2006.03.055 16893524

[B215] Linkenkaer-Hansen K. Monto S. Rytsälä H. Suominen K. Isometsä E. Köhkönen S. (2005). Breakdown of long range temporal correlations in theta oscillations in patients with major depressive disorder. J. Neurosci. 25, 10131–10137. 10.1523/JNEUROSCI.3244-05.2005 16267220 PMC6725784

[B216] Llinás R. Ribary U. Jeanmonod D. Kronberg E. Mitra P. P. (1999). Thalamocortical dysrhythmia: a neurological and neuropsychiatric syndrome characterized by magnetoencephalography. Proc. Natl. Acad. Sci. U.S.A. 96, 15222–15227. 10.1073/pnas.96.26.15222 10611366 PMC24801

[B217] Locatelli T. Cursi M. Liberati D. Franceschi J. Comi G. (1998). EEG coherence in Alzheimer’s disease. Electroencephalogr. Clin. Neurophysiol. 106, 229–237 10.1016/S0013-4694(97)00129-6 9743281

[B218] Lund T. R. Sponheim S. R. Iacono W. G. Clementz B. A. (1995). Internal consistency reliability of resting EEG power spectra in schizophrenic and normal subjects. Psychophysiology 32, 66–71. 10.1111/j.1469-8986.1995.tb03407.x 7878171

[B219] Lynall M. E. Bassett D. S. Kerwin R. McKenna P. J. Kitzbichler M. Muller U. (2010). Functional connectivity and brain networks in schizophrenia. J. Neurosci. 30, 9477–9487. 10.1523/JNEUROSCI.0333-10.2010 20631176 PMC2914251

[B220] Maltez J. Hyllienmark K. Nikulia V. V. Brismar T. (2004). Time course and variability of power in different frequency bands of the EEG during resting conditions. Clin. Neurophysiol. 34, 195–202. 10.1016/j.neucli.2004.09.003 15639128

[B221] Mars N. J. I. Lopes da Silva F. H. (1983). Propagation of seizure activity in kindled dogs. Electroencephalogr. Clin. Neurophysiol. 56, 194–209. 10.1016/0013-4694(83)90074-3 6191951

[B222] Mars N. J. I. Thompson P. M. Wilkus R. J. (1985). Spread of epileptic seizure activity in humans. Epilepsia 26, 85–94. 10.1111/j.1528-1157.1985.tb05192.x 3971952

[B223] Matsumoto A. Ichikawa Y. Kanayama N. Ohira H. Iidaka T. (2006). Gamma band activity and its synchronization reflect the dysfunctional emotional processing in alexithymic persons. Psychophysiology 43, 533–540. 10.1111/j.1469-8986.2006.00461.x 17076809

[B224] Mazzini L. (2004). Clinical applications of event-related potentials in brain injury. Phys. Med. Rehabil. Clin. N. Am. 15, 163–175. 10.1016/S1047-9651(03)00101-3 15029904

[B225] Mazzini L. Pisano F. Zaccala M. Miscio G. Gareri F. Galante M. (1999). Somatosensory and motor evoked potentials at different stages of recovery from severe traumatic brain injury. Arch. Phys. Med. Rehabil. 80, 33–39. 10.1016/S0003-9993(99)90304-0 9915369

[B226] Mazzini L. Zaccala M. Gareri F. Giordano A. Angelino E. (2001). Long latency auditory-evoked potentials in severe traumatic brain injury. Arch. Phys. Med. Rehabil. 82, 57–65. 10.1053/apmr.2001.18076 11239287

[B227] McCrea M. Prichep L. S. Powell M. R. Chabot R. Barr W. B. (2010). Acute effects and recovery after sports-related concussion: a quantitative brain electrical activity study. J. Head Trauma Rehabil. 25, 283–292. 10.1097/HTR.0b013e3181e67923 20611046

[B228] McDowell K. Chin-Teng L. Oie K. S. Tzyy-Ping J. Gordon S. Whitaker K. W. (2013). Real-world neuroimaging technologies. IEEE 1, 131–149.

[B229] McGraw K. O. Wong S. P. (1996). Forming inferences about some intraclass correlation coefficients. Psychol. Methods 1, 30–46 10.1037/1082-989X.1.1.30

[B230] Michelyannis S. Pachou E. Stam C. J. Breakspear M. Bitsios P. Vourkas M. (2006). Small-world networks and disturbed functional connectivity in schizophrenia. Schizophr. Res. 87, 60–66. 10.1016/j.schres.2006.06.028 16875801

[B231] Mizuno T. Takahashi T. Cho R. Y. Kikuchi M. Murata T. Takahashi K. (2010). Assessment of EEG dynamical complexity in Alzheimer’s disease using multiscale entropy. Clin. Neurophysiol. 121, 1438–1446. 10.1016/j.clinph.2010.03.025 20400371 PMC2914820

[B232] Moeller J. J. Tu B. Bazil C. W. (2011). Quantitative and qualitative analysis of ambulatory electroencephalography during mild traumatic brain injury. Arch. Neurol. 68, 1595–1598. 10.1001/archneurol.2011.1080 22159059

[B233] Montgomery E. A. Fenton G. W. McClelland R. J. MacFlynn G. Rutherford W. H. (1991). The psychobiology of minor head injury. Psychol. Med. 21, 375–384 10.1017/S0033291700020481 1876643

[B234] Morrow J. R. Jackson A. W. (1993). How ‘significant’ is your reliability? Res. Q. Exerc. Sport 64, 352–355 10.1080/02701367.1993.10608821 8235058

[B235] Mullen K. M. Vohr B. R. Katz K. H. Schneider K. C. Lacadie C. Hampson M. (2011). Preterm birth results in alterations in neural connectivity at age 16 years. Neuroimage 54, 2563–2570. 10.1016/j.neuroimage.2010.11.019 21073965 PMC3020252

[B236] Müller R. Büttner P. (1994). A critical discussion of intraclass correlation coefficients. Stat. Med. 13, 2465–2476. 10.1002/sim.4780132310 7701147

[B237] Müller T. J. Federspiel A. Horn H. Lövblad K. Lehmann C. Dierks T. (2005). The neurophysiological time pattern of illusionary visual perceptual transitions: a simultaneous EEG and fMRI study. Int. J. Psychophysiol. 55, 299–312. 10.1016/j.ijpsycho.2004.09.004 15708643

[B238] Münte T. F. Heinze H. J. (1994). Brain potentials reveal deficits of language processing after closed head injury. Arch. Neurol. 51, 482–493. 10.1001/archneur.1994.00540170058017 8179498

[B239] Murias M. Webb S. J. Greenson J. Dawson G. (2007). Resting state cortical connectivity reflected in EEG coherence in individuals with autism. Biol. Psychiatry 62, 270–273. 10.1016/j.biopsych.2006.11.012 17336944 PMC2001237

[B240] Nakamura T. Hillary F. G. Biswal B. B. (2009). Resting network plasticity following brain injury. PLoS ONE 4:e8220. 10.1371/journal.pone.0008220 20011533 PMC2788622

[B241] Naunheim R. S. Casner T. (2010). Novel method for detecting brain abnormality in a patient with epidural hematoma: a case report. Am. J. Emerg. Med. 28, e1–e386. 10.1016/j.ajem.2009.05.008 20223405

[B242] Naunheim R. S. Treaster M. English J. Casner T. Chabot R. (2010a). Use of brain electrical activity to quantify traumatic brain injury in the emergency department. Brain Inj. 24, 1324–1329. 10.3109/02699052.2010.506862 20722504

[B243] Naunheim R. S. Treaster M. English J. Casner T. (2010b). Automated electroencephalogram (EEG) identifies abnormalities in the Emergency Department. Am. J. Emerg. Med. 29, 845–848. 10.1016/j.ajem.2010.03.010 20825903

[B244] Nikulin V. V. Brismar T. (2004). Comment on “Multscale entropy analysis of complex physiologic time series”. Phys. Rev. Lett. 92, 089803 10.1103/PhysRevLett.92.089803 14995828

[B245] Nolte G. Bai O. Wheaton L. Mari Z. Vorbach S. Hallet M. (2004). Identifying true brain interaction from EEG data using the imaginary part of coherency. Clin. Neurophsyiol. 115, 2292–2307. 10.1016/j.clinph.2004.04.029 15351371

[B246] Nolte G. Ziehe A. Nikulin V. V. Schlögl A. Krämer N. Brismar T. (2008). Robustly estimating the flow direction of information in complex physical systems. Phys. Rev. Lett. 100, 234101. 10.1103/PhysRevLett.100.234101 18643502

[B247] Nunez P. L. Silberstein R. B. Shi Z. P. Carpenter M. R. Srinivasan R. Tucker D. M. (1999). EEG coherency II: experimental comparisons of multiple measures. Clin. Neurophsyiol. 110, 469–486. 10.1016/S1388-2457(98)00043-1 10363771

[B248] Nunez P. L. Srinivasan R. Westdorp A. F. Wijesinghe R. S. Tucker D. M. Silberstein R. B. (1997). EEG coherency. I: statistics, reference electrode, volume conduction, Laplacians, cortical imaging and interpretation at multiple scales. Electroencephalogr. Clin. Neurophysiol. 103, 499–515. 10.1016/S0013-4694(97)00066-7 9402881

[B249] Nuwer M. (1997). Assessment of digital EEG, quantitative EEG, and EEG brain mapping: report of the American Academy of Neurology and the American Clinical Neurophysiology Society. Neurology 49, 277–292. 10.1212/WNL.49.1.277 9222209

[B250] Nuwer M. R. Hovda D. A. Schrader L. M. Vespa P. M. (2005). Routine and quantitative EEG in mild traumatic brain injury. Clin. Neurophysiol. 116, 2001–2025 10.1016/j.clinph.2005.05.008 16029958

[B251] O’Donnell B. F. Hetrick W. P. Vohs J. L. Krisnan G. P. Carroll C. A. Shekhar A. (2004). Neural synchronization deficits to auditory stimulation in bipolar disorder. Neuroreport 15, 1369–1372. 10.1097/01.wnr.0000127348.64681.b2 15167568

[B252] Olbrich H. M. Nau H. E. Zerbin D. Lanczos L. Lodemann E. Engelmeier M. P. (1986). Clinical application of event related potentials in patients with brain tumours and traumatic head injuries. Acta Neurochir. (Wien) 80, 116–122. 10.1007/BF01812285 3716890

[B253] O’Neil B. Prichep L. S. Naunheim R. Chabot R. (2012). Quantitative brain electrical activity in the initial screening of mild traumatic brain injuries. West. J. Emerg. Med. 13, 394–400. 10.5811/westjem.2011.12.6815 23359586 PMC3556946

[B254] Onofrj M. Curatola L. Malatesta G. Bazzano S. Colamartino P. Flugente T. (1991). Reduction of P3 latency during outcome of post-traumatic amnesia. Acta Neurol. Scand. 83, 273–279. 10.1111/j.1600-0404.1991.tb04700.x 2063648

[B255] Orekhova E. V. Stroganova T. A. Nygren G. Tsetlin M. M. Posikera I. W. Gillberg C. (2007). Excess of high frequency electroencephalogram oscillations in boys with autism. Biol. Psychiatry 62, 1022–1029. 10.1016/j.biopsych.2006.12.029 17543897

[B256] Papanicolaou A. C. Levin H. S. Eisenberg H. M. Moore B. D. Goethe K. E. High WM. Jr (1984). Evoked potential correlates of posttraumatic amnesia after closed head injury. Neurosurgery 14, 676–678. 10.1097/00006123-198406000-00005 6462402

[B257] Papo D. (2013). Time scales in cognitive neuroscience. Front. Physiol. 4:86 10.3389/fphys.2013.00086 23626578 PMC3630296

[B258] Park C.-A. Kown R.-J. Kim S. Jang H.-R. Chae J.-H. Kim T. (2007). “Decreased phase synchronization of the EEG in patients with major depressive disorder,” in IFMBE Proceedings, Vol. 14, eds Magjurevic R. Nagel J. H. (Seoul: World Congress on Medical Physics and Biomedical Engineering), 1095–1098.

[B259] Park C. H. Wang S. M. Lee H. K. Kweon Y. S. Lee C. T. Kim K. T. (2014). Affective state-dependent changes in the brain functional network in major depressive disorder. Soc. Cogn. Affect. Neurosci. 9, 1404–1412. 10.1093/scan/nst126 24249787 PMC4158376

[B260] Penttonen M. Buzsáki G. (2003). Natural logarithmic relationship between brain oscillators. Thalamus Relat. Syst. 2, 145–152 10.1016/S1472-9288(03)00007-4

[B261] Perdikis D. Huys R. Jirsa V. (2011a). Complex processes from dynamical architectures with time-scale hierarchy. PLoS ONE 6:e16589. 10.1371/journal.pone.0016589 21347363 PMC3037373

[B262] Perdikis D. Huys R. Jirsa K. (2011b). Time scale hierarchies in the functional organization of complex behaviors. PLoS Comput. Biol. 7:e1002198. 10.1371/journal.pcbi.1002198 21980278 PMC3182871

[B263] Perlstein W. M. Larson M. J. Dotson V. M. Kelly K. G. (2006). Temporal dissociation of components of cognitive control dysfunction in severe TBI: ERPs and the cued-Stroop task. Neuropsychologia 44, 260–274. 10.1016/j.neuropsychologia.2005.05.009 15979655

[B264] Pinault D. (2008). N-methyl d-aspartate receptor antagonists ketamine and MK-801 induce wake related aberrant γ oscillations in the rat neocortex. Biol. Psychiatry 63, 730–735. 10.1016/j.biopsych.2007.10.006 18022604

[B265] Pincus S. M. (1991). Approximate entropy as a measure of system complexity. Proc. Natl. Acad. Sci. U.S.A. 88, 2297–2301. 10.1073/pnas.88.6.2297 11607165 PMC51218

[B266] Pincus S. M. Goldberger A. L. (1994). Physiological time-series analysis: what does regularity quantify? Am. J. Physiol. 266, H1643–H1656. 8184944 10.1152/ajpheart.1994.266.4.H1643

[B267] Pollock V. E. Schneider L. S. Lyness S. A. (1991). Reliability of topographic quantitative EEG amplitude in healthy late-middle-aged and elderly subjects. Electroencephalogr. Clin. Neurophysiol. 79, 20–26. 10.1016/0013-4694(91)90152-T 1713548

[B268] Polo M. D. Newton P. Rogers D. Escera C. Butler S. (2002). ERPs and behavioural indices of long-term preattentive and attentive deficits following closed skull head injury. Neuropsychologia 40, 2350–2359. 10.1016/S0028-3932(02)00127-6 12417464

[B269] Ponten S. C. Bartolemi F. Stam C. J. (2007). Small-world networks and epilepsy: graph theoretical analysis of intracerebrally recorded mesial temporal lobe seizures. Clin. Neurophysiol. 118, 918–927. 10.1016/j.clinph.2006.12.002 17314065

[B270] Pontifex M. B. O’Connor P. M. Broglio S. P. Hillman C. H. (2009). The association between mild traumatic brain history and cognitive control. Neuropsychologia 47, 3210–3216. 10.1016/j.neuropsychologia.2009.07.021 19664646

[B271] Potter D. D. Barrett K. (1999). Assessment of mild head injury with ERPs and neuropsychological tasks. J. Psychophysiol. 13, 173–189 10.1027//0269-8803.13.3.173

[B272] Potter D. D. Bassett M. R. Jory S. H. Barrett K. (2001). Changes in event-related potentials in a three-stimulus auditory oddball task after mild head injury. Neuropsychologia 39, 1464–1472. 10.1016/S0028-3932(01)00057-4 11585614

[B273] Potter D. D. Jory S. H. Bassett M. R. A. Barrett K. Mychalkiw W. (2002). Effect of mild head injury on event related potentials of Stroop task performance. J. Int. Neuropsychol. Soc. 8, 828–837. 10.1017/S1355617702860118 12240747

[B274] Pratap-Chand R. Sinniah M. Salem F. (1988). Cognitive evoked potential (P300): a metric for cerebral concussion. Acta Neurol. Scand. 78, 185–189. 10.1111/j.1600-0404.1988.tb03643.x 3227804

[B275] Prichard D. Theiler J. (1994). Generating surrogate data for time series with several simultaneously measured variables. Phys. Rev. Lett. 73, 951–954 10.1103/PhysRevLett.73.951 10057582

[B276] Prichep L. S. Jacquin A. Filipenko J. Dastidar S. G. Zabele S. Vodencarevic A. (2012a). Classification of traumatic brain injury severity using informed data reduction in a series of binary classifier algorithms. IEEE Trans. Neural Syst. Rehabil. Eng. 20, 806–822. 10.1109/TNSRE.2012.2206609 22855231

[B277] Prichep L. S. McCrea M. Barr W. Powell M. Chabot R. J. (2012b). Time course of clinical and electrophysiological recovery after sport-related concussion. J. Head Trauma Rehabil. 28, 266–273. 10.1097/HTR.0b013e318247b54e 22588360

[B278] Prichep L. S. John E. R. Ferris S. H. Rausch L. Fang Z. Cancro R. (2006). Prediction of longitudinal cognitive decline in normal elderly with subjective complaints using electrophysiological imaging. Neurobiol. Aging 27, 471–481. 10.1016/j.neurobiolaging.2005.07.021 16213630

[B279] Ramdani S. Seigle B. Lagarde J. Bouchara F. Bernard P. L. (2009). On the use of sample entropy to analyze human postural sway data. Med. Eng. Phys. 31, 1023–1031. 10.1016/j.medengphy.2009.06.004 19608447

[B280] Ramos-Loyo J. González-Garrido A. A. Sánchez-Loyo L. M. (2009). Event-related potentials and event-related oscillations during identity and facial emotional processing in schizophrenia. Int. J. Psychophysiol. 71, 84–90. 10.1016/j.ijpsycho.2008.07.008 18727940

[B281] Rapp P. E. Cellucci C. J. Watanabe T. A. A. Albano A. M. Schmah T. I. (2001). Surrogate data pathologies and the false-positive rejection of the null hypothesis. Int. J. Bifurcat. Chaos 11, 983–997. 10.1142/S021812740100250X12769438

[B282] Rapp P. E. Curley K. C. (2012). Is a diagnosis of “mild traumatic brain injury” a category mistake? J. Trauma Acute Care Surg. 73(2 Suppl. 1), S13–S23. 10.1097/TA.0b013e318260604b 22847083

[B283] Rapp P. E. Rosenberg B. M. Keyser D. O. Nathan D. Toruno K. M. Cellucci C. J. (2013a). Patient characterization protocols for psychophysiological studies of traumatic brain injury and post-TBI psychiatric disorders. Front Neurol. 4:91. 10.3389/fneur.2013.00091 23885250 PMC3717660

[B284] Rapp P. E. Darmon D. M. Cellucci C. J. (2013b). “Hierarchical transition chronometries in the human central nervous system,” in Proceedings International Conference on Nonlinear Theory and Applications, Santa Fe, NM.

[B285] Rappaport M. Hemmerle A. V. Rappaport M. L. (1990). Intermediate and long latency SEPs in relation to clinical disability in traumatic brain in jury patients. Clin. Electroencephalogr. 21, 188–191. 10.1177/155005949002100406 2225467

[B286] Raz A. Vaadia E. Bergman H. (2000). Firing patterns and correlations of spontaneous discharge of pallidal neurons in the normal and the tremulous 1-methyl-4-phenyl-1,2,3,6-tetrahydropyridine vervet model of parkinsonism. J. Neurosci. 20, 8559–8571. 11069964 10.1523/JNEUROSCI.20-22-08559.2000 PMC6773163

[B287] Reinvang I. (1999). Cogntive event-related potentials in neuropsychological assessment. Neuropsychol. Rev. 9, 321–348 10.1023/A:1021638723486 10667449

[B288] Reinvang I. Nordby H. Nielsen C. S. (2000). Information processing deficits in head injury assessed with ERPs reflecting early and late processing stage. Neuropsychologia 38, 995–1005. 10.1016/S0028-3932(99)00153-0 10775710

[B289] Reshef D. N. Reshef Y. A. Finucane H. K. Grossman S. R. Mcvean G. Turnbaugh P. J. (2011). Detecting novel associations in large data sets. Science 334, 1518–1524 10.1126/science.1205438 PMC3325791 22174245

[B290] Reuter B. M. Linke D. B. (1989). “P300 and coma,” in Topographic Brain Mapping of EEG and Evoked Potentials, ed. Maurer K. (New York, NY: Springer-Verlag), 192–196.

[B291] Richardson M. J. Lopresti-Goodman S. Mancini M. Kay B. Schmidt R. C. (2008). Comparing the attractor strength of intra- and interpersonal interlimb coordination using cross recurrence analysis. Neurosci. Lett. 438, 340–345. 10.1016/j.neulet.2008.04.083 18487016

[B292] Richman J. S. Moorman J. R. (2000). Physiological time-series analysis using approximate entropy and sample entropy. Am. J. Physiol. Heart Circ. Physiol. 278, H2039–H2049. 10843903 10.1152/ajpheart.2000.278.6.H2039

[B293] Rigg J. L. Mooney S. R. (2011). Concussions and the military: issues specific to service members. PM R 3(10 Suppl. 2), S380–S386. 10.1016/j.pmrj.2011.08.005 22035680

[B294] Rippon G. Brock J. Brown C. Boucher J. (2007). Disordered connectivity in the autistic brain: challenges for the “new psychophysiology”. Int. J. Psychophysiol. 63, 164–172. 10.1016/j.ijpsycho.2006.03.012 16820239

[B295] Rizzo P. A. Amabile G. Caporali M. Spadaro M. Zanasi M. Morocutti C. (1978). A CNV study in a group of patients with traumatic head injuries. Electroencephalogr. Clin. Neurophysiol. 45, 281–285. 10.1016/0013-4694(78)90012-3 78838

[B296] Roche R. A. P. Dockree P. M. Garavan H. Foxe J. J. Robertson I. H. O’Mara S. M. (2004). EEG alpha power changes reflect response inhibition deficits after traumatic brain injury (TBI) in humans. Neurosci. Lett. 362, 1–5. 10.1016/j.neulet.2003.11.064 15147767

[B297] Rockstroh B. S. Wienbruch C. Ray W. J. Elbert T. (2007). Abnormal oscillatory brain dynamics in schizophrenia: a sign of deviant communication in an neural network? BMC Psychiatry 7:44. 10.1186/1471-244X-7-44 17760978 PMC2034549

[B298] Romano M. C. Thiel M. Kurths J. von Bloh W. (2004). Multivariate recurrence plots. Phys. Lett. A 330, 214–223 10.1016/j.physleta.2004.07.066

[B299] Roopun A. K. Cunningham M. O. Rucca C. Alter K. Traub R. D. Whittington M. A. (2008). Region-specific changes in gamma and beta2 rhythms in NMDA receptor dysfunction models of schizophrenia. Schizophr. Bull. 34, 962–973. 10.1093/schbul/sbn059 18544550 PMC2518640

[B300] Rosenbaum R. S. Furey M. L. Horwitz B. Grady C. L. (2008). Altered connectivity among emotion-related brain regions during short-term memory in Alzheimer’s disease. Neurobiol. Aging 31, 780–786. 10.1016/j.neurobiolaging.2008.06.002 18639365 PMC2842478

[B301] Rubinov M. Sporns O. Van Leeuwen C. Breakspear M. (2009). Symbiotic relationship between brain structure and dynamics. BMC Neurosci. 10:55. 10.1186/1471-2202-10-55 19486538 PMC2700812

[B302] Rugg M. D. Cowan C. P. Nagy M. E. Milner A. D. Jacobson I. Brooks D. N. (1988). Event related potentials from closed head injury patients in an auditory “oddball” task: evidence of dysfunction in stimulus categorization. J. Neurol. Neurosurg. Psychiatry 51, 691–698. 10.1136/jnnp.51.5.691 3404166 PMC1033079

[B303] Rugg M. D. Pickles C. D. Potter D. D. Doyle M. C. Pentland B. Roberts R. C. (1993). Cogntive brain potentials in a three stimulus auditory “oddball” task after closed head injury. Neuropsychologica 31, 373–393. 10.1016/0028-3932(93)90161-R 8502373

[B304] Rulkov N. F. Sushchik M. M. Tsimring L. S. Abarbanel H. D. I. (1995). Generalized synchronization of chaos in directionally coupled chaotic systems. Phys. Rev. E Stat. Phys. Plasmas Fluids Relat. Interdiscip. Topics 51, 980–994 10.1103/PhysRevE.51.980 9962737

[B305] Sabeti M. Katebi S. Boostani R. (2009). Entropy and complexity measures for EEG signal classification of schizophrenic and control participants. Artif. Intell. Med. 47, 263–274. 10.1016/j.artmed.2009.03.003 19403281

[B306] Salinsky M. C. Oken B. S. Morehead L. (1991). Test-retest reliability in EEG frequency analysis. Electroencephalogr. Clin. Neurophysiol. 79, 382–392. 10.1016/0013-4694(91)90203-G 1718711

[B307] Sangal R. B. Sangal J. M. (1996). Closed head injury patients with mild cognitive complaints without neurological or psychiatric findings have abnormal visual P300 latencies. Biol. Psychiatry 39, 305–307 10.1016/0006-3223(95)00447-5 8645780

[B308] Sazonov A. V. Ho C. K. Bergmans J. W. M. Arends J. B. A. M. Griep P. A. M. Verbitskiy E. A. (2009). An investigation of phase locking index for measuring interdependency of cortical source signals recorded in the EEG. Biol. Cyben. 100, 129–146. 10.1007/s00422-008-0283-4 19152066 PMC2792353

[B309] Schiff S. J. (2005). Dangerous phase. Neuroinformatics 3, 315–318. 10.1385/NI:3:4:315 16284414 PMC1469234

[B310] Schnitzler A. Gross J. (2005). Normal and pathological oscillatory communication in the brain. Nat. Rev. Neurosci. 6, 285–296. 10.1038/nrn1650 15803160

[B311] Schreiber T. (2000). Measuring information transfer. Phys. Rev. Lett. 85, 461–464. 10.1103/PhysRevLett.85.461 10991308

[B312] Segalowitz S. J. Bernstein D. M. Lawson S. (2001). P300 event-related potential decrements in well-functioning university students with mild head injury 6 years post-injury. Brain Cogn. 45, 342–356. 10.1006/brcg.2000.1263 11305878

[B313] Segalowitz S. J. Dywan J. Unsal A. (1997). Attentional factors in response time variability after traumatic brain injury: an ERP study. J. Int. Neuropsychol. Soc. 3, 95–107. 9126851

[B314] Segalowitz S. J. Unsal A. Dywan J. (1992). CNV evidence for the distinctiveness of frontal and posterior processes in traumatic brain injury. J. Clin. Exp. Neuropsychol. 14, 545–565. 10.1080/01688639208402844 1400918

[B315] Shannon C. E. (1948). A mathematical theory of communication. Bell Syst. Tech. J. 27, 379–423 10.1002/j.1538-7305.1948.tb01338.x

[B316] Shaw M. (2002). Abnormal functional connectivity in posttraumatic stress disorder. Neuroimage 15, 661–674. 10.1006/nimg.2001.1024 11848709

[B317] Shrout P. E. Fleiss J. L. (1979). Intraclass correlations: uses in assessing rater reliability. Psychol. Bull. 86, 420–428. 10.1037/0033-2909.86.2.420 18839484

[B318] Siegel S. J. Ehrlichman R. S. Phillips J. M. Liang Y. Turetsky B. I. Aillon D. D. (2006). Ketamine causes persistent oscillations similar to schizophrenia and reduced hippocampal glutamate concentrations in mice. Biol. Psychiatry 59, 245S.

[B319] Sims C. (1972). Money, income and causality. Am. Econ. Rev. 62, 540–552.

[B320] Sinai Y. A. G. (1959). On the concept of entropy of a dynamical system. Dokl. Acad. Nauk USSR 124, 768–781.

[B321] Slewa-Younan S. Green A. M. Baguley I. J. Felminham K. L. Haig A. R. Gordon E. (2002). Is ‘gamma’ (40 Hz) synchronous activity disturbed in patients with traumatic brain injury? Clin. Neurophysiol. 113, 1640–1646. 10.1016/S1388-2457(02)00239-0 12350441

[B322] Sloan E. P. Fenton G. W. (1993). EEG power spectra and cognitive change in geriatric psychiatry: a longitudinal study. Electroencephalogr. Clin. Neurophysiol. 86, 361–367. 10.1016/0013-4694(93)90131-E 7686470

[B323] Slobounov S. Sebastianelli W. Hallett M. (2012). Residual brain dysfunction observed one year post mild traumatic brain injury combined EEG and balance study. Clin. Neurophysiol. 123, 1755–1756. 10.1016/j.clinph.2011.12.022 22361265 PMC3513284

[B324] Smit D. J. Stam C. J. Posthuma D. Boomsma D. I. de Geus E. J. (2008). Heritability of “small-world” networks in the brain: a graph theoretical analysis of resting-state EEG functional connectivity. Hum. Brain Mapp. 29, 1368–1378. 10.1002/hbm.20468 18064590 PMC6870849

[B325] Solbakk A.-K. Reinvang I. Andersson S. (2002). Assessment of P3a and P3b after moderate to severe brain injury. Clin. Electroencephalogr. 33, 102–110. 10.1177/155005940203300306 12192659

[B326] Solbakk A.-K. Reinvang I. Nielsen C. Sundet K. (1999). ERP indicators of disturbed attention in mild closed head injury: a frontal lobe syndrome? Psychophysiology 36, 802–817. 10.1111/1469-8986.3660802 10554593

[B327] Solbakk A.-K. Reinvang I. Nielsen C. S. (2000). ERP indices of resource allocation difficulties in mild head injury. J. Clin. Exp. Neuropsychol. 22, 743–760. 10.1076/jcen.22.6.743.953 11320433

[B328] Solbakk A.-K. Reinvang I. Svebak S. Nielsen C. S. Sundet K. (2005). Attention to affective pictures in closed head injury: event-related brain potentials and cardiac responses. J. Clin. Exp. Neurospychol. 27, 205–223. 10.1080/13803390490515739 15903151

[B329] Spencer K. M. (2005). “Averaging, detection and classification of single-trial ERPs,” in Event Related Potentials: A Methods Handbook, ed. Handy T. C. (Cambridge, MA: MIT Press), 209–227.

[B330] Spencer K. M. Nestor P. G. Niznikiewicz M. A. Salisbury D. F. Shenton M. E. Mccarley R. W. (2003). Abnormal neural synchrony in schizophrenia. J. Neurosci. 23, 7407–7411. 12917376 10.1523/JNEUROSCI.23-19-07407.2003 PMC2848257

[B331] Spencer K. M. McCarley R. W. (2006). Neuropsychiatric abnormalities: a new vista from studies on fundamental properties of neural communication. Psychiatr. Times 25 Available at: http://www.psychiatrictimes.com/display/article/10168/46711

[B332] Spencer K. M. Nestor P. G. Perlmutter R. Niznuikiewicz M. A. Klump M. C. Frumin M. (2004). Neural synchrony indexes, disordered perception and cognition in schizophrenia. Proc. Natl. Acad. Sci. U.S.A. 101, 17288–17293. 10.1073/pnas.0406074101 15546988 PMC535363

[B333] Spencer K. M. Niznikiewicz M. A. Shenton M. E. McCarley R. W. (2008a). Sensory-evoked gamma oscillations in chronic schizophrenia. Biol. Psychiatry 63, 744–747. 10.1016/j.biopsych.2007.10.017 18083143 PMC2330275

[B334] Spencer K. M. Salisbury D. F. Shenton M. E. McCarley R. W. (2008b). Gamma-band auditory steady-state responses are impaired in first episode patients. Biol. Psychiatry 64, 369–375. 10.1016/j.biopsych.2008.02.021 18400208 PMC2579257

[B335] Spikman J. M. van der Naalt J. van Weerden T. W. van Zomeren A. H. (2004). Indices of slowness of information processing in head injury patients: tests for selective attention related to ERP latencies. J. Int. Neuropsychol. Soc. 10, 851–861. 10.1017/S1355617704106061 15637776

[B336] Sponheim S. R. McGuire K. A. Kang S. S. Davenport N. D. Aviyente S. Bernat E. M. (2011). Evidence of disrupted functional connectivity in the brain after combat-related blast injury. Neuorimage 54, S21–S29. 10.1016/j.neuroimage.2010.09.007 20851190

[B337] Stam C. J. (2005). Nonlinear dynamical analysis of EEG and MEG: review of an emerging field. Clin. Neurophysiol 116, 2266–2301. 10.1016/j.clinph.2005.06.011 16115797

[B338] Stam C. J. de Haan W. Daffertshofer A. Jones B. F. Manshanden I. van Cappellen van Walsum A. M. (2009). Graph theoretical analysis of magentoencephalographic functional connectivity in Alzheimer’s disease. Brain 132, 213–224. 10.1093/brain/awn262 18952674

[B339] Stam C. J. Jones B. F. Manshanden I. Van Cappellen Van Walsum A. M. Montez T. Verbunt J. P. (2006). Magnetoencephalographic evaluation of resting-state functional connectivity in Alzheimer’s disease. Neuroimage 32, 1335–1344. 10.1016/j.neuroimage.2006.05.033 16815039

[B340] Stam C. J. Jones B. F. Nolte G. Breakspear M. Scheltens P. H. (2007). Small-world networks and functional connectivity in Alzheimer’s disease. Cereb. Cortex 17, 92–99 10.1093/cercor/bhj127 16452642

[B341] Stam C. J. Van Der Made Y. Pijnenburg Y. A. Scheltens P. (2003). EEG synchronization in mild cognitive impairment and Alzheimer’s disease. Acta Neurol. Scand. 108, 90–96. 12859284 10.1034/j.1600-0404.2003.02067.x

[B342] Stam C. J. van Dijk B. W. (2002). Synchronizaiton likelihood: an unbiased measure of generalized synchronization in multivariate data sets. Physica D 163, 236–251. 10.1016/S0167-2789(01)00386-417023181

[B343] Steuer R. Molgedey L. Ebeling W. Jiménez-Montaño M.-A. (2001). Entropy and optimal partition for data analysis. Eur. Phys. J. B 19, 265–269 10.1007/s100510170335

[B344] Stoffers D. Bosboom J. L. W. Deijen J. B. Walters E. C. Berendse H. W. Stam C. J. (2007). Slowing of oscillatory brain activity is a stable characteristic of Parkinson’s disease without dementia. Brain 130, 1847–1860. 10.1093/brain/awm034 17412733

[B345] Stratford P. W. (1989). Confidence limits for your ICC. Phys. Ther. 69, 237–238. 2919194 10.1093/ptj/69.3.237

[B346] Symond M. B. Harris A. W. F. Gordon E. Williams L. M. (2005). “Gamma synchrony” in first-episode schizophrenia: a disorder of temporal connectivity? Am. J. Psychiatry 162, 459–465. 10.1176/appi.ajp.162.3.459 15741462

[B347] Takahashi T. Cho R. Y. Mizuno T. Kikuchi M. Murata T. Takahashi K. (2010). Antipsychotics reverse abnormal EEG complexity in drug-naive schizophrenia: a multiscale entropy analysis. Neuroimage 51, 173–182. 10.1016/j.neuroimage.2010.02.009 20149880 PMC2849166

[B348] Takens F. (1981). “Detecting strange attractors in turbulence,” in Lecture Notes in Mathematics, Vol. 898, eds Rand D. A. Young L. S. (New York, NY: Springer-Verlag), 365–381.

[B349] Tebano M. T. Cameroni M. Gallozzi G. Loizzo A. Palazzino G. Pezzini G. (1988). EEG spectral analysis after minor head injury in man. Electroencephalogr. Clin. Neurophysiol. 70, 185–189. 10.1016/0013-4694(88)90118-6 2456196

[B350] Thatcher R. W. (2000). EEG operant conditioning (biofeedback) and traumatic brain injury. Clin. Electroencephalogr. 31, 38–44. 10.1177/155005940003100110 10638351

[B351] Thatcher R. W. (2006). “Electroencephalography and mild traumatic brain injury,” in Foundations of Sports Related Brain Injuries, eds Slobounov S. M. Sebastianelli W. J. (New York, NY: Springer-Verlag), 241–266.

[B352] Thatcher R. W. Biver C. Gomez J. F. North D. Curtin R. Walker R. A. (2001). Estimation of the EEG power spectrum using MRI T2 relaxation time in traumatic brain injury. Clin. Neurophysiol. 112, 1729–1745. 10.1016/S1388-2457(01)00609-5 11514257

[B353] Thatcher R. W. Biver C. J. North D. M. (2003). Quantitative EEG and the Frye and Daubert standards of admissibility. Clin. Electroencephalogr. 34, 39–53. 12784902 10.1177/155005940303400203

[B354] Thatcher R. W. Cantor D. S. McAlaster R. Geisler F. Krause P. (1991). Comprehensive predictions of outcome in closed head injury: the development of prognostic equations. Ann. N. Y. Acad. Sci. 620, 82–104. 10.1111/j.1749-6632.1991.tb51576.x 2035948

[B355] Thatcher R. W. Moore N. John E. R. Duffy F. Hughes J. R. Krieger M. (1999). QEEG and traumatic brain injury: rebuttal of the American Academy of Neurology 1997 report by the EEG and Clinical Neuroscience Society. Clin. Electroencephalogr. 30, 94–98 10.1177/155005949903000304 10578471

[B356] Thatcher R. W. Walker R. A. Gerson I. Geisler F. (1989). EEG discriminant analyses of mild head trauma. Electroencephalogr. Clin. Neurophysiol. 73, 93–106. 10.1016/0013-4694(89)90188-0 2473888

[B357] Theiler J. Eubank S. Longtin A. Galdrikian B. Farmer J. D. (1992). Testing for nonlinearity in time series: the method of surrogate data. Physica 58D, 77–94.

[B358] Thornton K. (2003). The electrophysiological effects of a bain injury on auditory memory functioning: qEEG correlates of impaired memory. Arch. Clin. Neuropsychol. 18, 363–378. 10.1093/arclin/18.4.363 14591452

[B359] Thornton K. E. (1999). Exploratory investigation into mild brain injury and discriminant analysis with high frequency bands (32-64 Hz). Brain Inj. 13, 477–488. 10.1080/026990599121395 10462146

[B360] Tomarken A. Davidson R. J. Wheeler R. E. Kinney L. (1992). Psychometric properties of resting anterior EEG asymmetry: temporal stability and internal consistency. Psychophysiology 29, 576–592. 10.1111/j.1469-8986.1992.tb02034.x 1410187

[B361] Tomkins O. Feintuch A. Benifla M. Cohen A. Friedman A. Shelef I. (2011). Blood-brain barrier breakdown following traumatic brain injury: a possible role in posttraumatic epilepsy. Cardiovasc. Psychiatry Neurol. 2011:765923 10.1155/2011/765923 21436875 PMC3056210

[B362] Tononi G. Edelman G. M. (2000). Schizophrenia and the mechanisms of conscious integration. Brain Res. Rev. 31, 391–400. 10.1016/S0165-0173(99)00056-9 10719167

[B363] Tononi G. Sporns O. Edelman G. M. (1994). A measure for brain complexity: relating functional segregation and integration in the nervous system. Proc. Natl. Acad. Sci. U.S.A. 91, 5033–5037. 10.1073/pnas.91.11.5033 8197179 PMC43925

[B364] Trudeau D. L. Anderson J. Hansen L. M. Shagalov D. N. Schmoller J. Nugent S. (1998). Findings of mild traumatic brain injury in combat veterans with PTSD and a history of concussion. J. Neuropsychiatry Clin. Neurosci. 10, 308–313. 10.1176/jnp.10.3.308 9706538

[B365] Tsirka V. Simos P. G. Vakis A. Kanatsouli K. Vourkas M. Erimaki S. (2011). Mild traumatic brain injury: graph-model characterization of brain networks for episodic memory. Int. J Psychophysiol. 79, 89–96. 10.1016/j.ijpsycho.2010.09.006 20863861

[B366] Uhlhaas P. J. Haenschel C. Nikolic D. Singer W. (2008). The role of oscillations and synchrony in cortical networks and their putative relevance for the pathophysiology of schizophrenia. Schizophr. Bull. 34, 927–943. 10.1093/schbul/sbn062 18562344 PMC2632472

[B367] Uhlhaas P. J. Singer W. (2006). Neural synchrony in brain disorders: relevance for cognitive dysfunctions and pathophysiology. Neuron 52, 155–168. 10.1016/j.neuron.2006.09.020 17015233

[B368] Unsal A. Segalowitz S. J. (1995). Sources of P300 attenuation after head injury: single trial amplitude, latency jitter and EEG power. Psychophysiology 32, 249–256. 10.1111/j.1469-8986.1995.tb02953.x 7784533

[B369] Van Albada S. J. Rennie C. J. Robinson P. A. (2007). Variability of model-free and model-based quantitative measures of EEG. J. Integr. Neurosci. 6, 279–307. 10.1142/S0219635207001520 17622982

[B370] Van Albada S. J. Robinson P. A. (2007). Transformation of arbitrary distributions to the normal distribution with application to EEG test-retest reliability. J. Neurosci. Methods 161, 205–211. 10.1016/j.jneumeth.2006.11.004 17204332

[B371] van Dellen E. Douw L. Baayen J. C. Heimans J. J. Ponten S. C. Vandertop W. B. (2009). Long-term effects of temporal lobe epilepsy on local neural networks: a graph theoretical analysis of corticography recordings. PLoS ONE 4:e8081. 10.1371/journal.pone.0008081 19956634 PMC2778557

[B372] van der Stelt O. Belger A. (2007). Applications of electroencephalography to the study of cognitive and brain functions in schizophrenia. Schizophr. Bull. 33, 955–970. 10.1093/schbul/sbm016 17363840 PMC2632335

[B373] Vastano J. A. Swinney H. L. (1988). Information transport in spatiotemporal systems. Phys. Rev. Lett. 60, 1773–1776 10.1103/PhysRevLett.60.1773 10038137

[B374] Vidal C. Nicolson R. DeVito T. J. Hayashi K. M. Geaga J. A. Drost D. J. (2006). Mapping corpus callosum deficits in autism: an index of aberrant cortical connectivity. Biol. Psychiatry 60, 218–225. 10.1016/j.biopsych.2005.11.011 16460701

[B375] Vierling-Claassen D. Siekmeier P. Stufflebeam S. Kopell N. (2008). Modeling GABA alterations in schizophrenia: a link between impaired inhibiton and altered gamma and beta range auditory entrainment. J. Neurophysiol. 99, 2656–2671. 10.1152/jn.00870.2007 18287555 PMC2679675

[B376] Viggiano M. P. (1996). Event-related potentials in brain-injured patients with neuropsychological disorders: a review. J. Clin. Exp. Neurospsychol. 18, 631–647. 10.1080/01688639608408288 8941850

[B377] von Bierbrauer A. Weissenborn K. (1998). P300 after minor head injury (a follow-up examination). Acta Neurol. Belg. 98, 21–26. 9606435

[B378] von Bierbrauer A. Weissenborn K. Hinrichs H. Scholz M. Künkel H. (1992). Automatic (computer-assisted) EEG analysis in comparison with visual EEG analysis in patients following minor cranio-cerebral trauma (a follow-up study). EEG EMG Z. Elektroenzephalogr. Elektromyogr. Verwandte Geb. 23, 151–157. 1425393

[B379] Wang J. T. Young G. B. Connolly J. F. (2004). Prognostic value of evoked responses and event-related brain potentials in coma. Can. J. Neurol. Sci. 31, 438–450. 10.1017/S0317167100003619 15595246

[B380] Watanabe T. A. A. Cellucci C. J. Kohegyi E. Bashore T. R. Josiassen R. C. Greenbaun N. N. (2003). The algorithmic complexity of multichannel EEGs is sensitive to changes in behavior. Psychophysiology 40, 77–97. 10.1111/1469-8986.00009 12751806

[B381] Watson S. J. (ed.) (1995). Biology of Schizophrenia and Affective Disease. Washington, DC: Amer. Psychiatric Press.

[B382] Watts D. J. Strogatz S. H. (1998). Collective dynamics of small-world networks. Nature 393, 440–442 10.1038/30918 9623998

[B383] Welsh J. P. Ahn E. S. Placantonakis D. G. (2005). Is autism due to brain desynchronization? Int. J. Dev. Neurosci. 23, 253–263. 10.1016/j.ijdevneu.2004.09.002 15749250

[B384] Wendling F. Ansari-Asl K. Bartolomei F. Senhadji L. (2009). From EEG signals to brain connectivity: a model based evaluation of interdependence measures. J. Neurosci. Methods 183, 9–18. 10.1016/j.jneumeth.2009.04.021 19422854

[B385] Werner R. Vanderzant C. (1991). Multimodality evoked potential testing in acute mild closed head injury. Arch. Phys. Med. Rehabil. 72, 31–34. 1985621

[B386] Whittington M. A. (2008). Can brain rhythms inform on underlying pathology in schizophrenia? Biol. Psychiatry 63, 728–729 10.1016/j.biopsych.2008.02.007 18371495

[B387] Wickelgren I. (2005). Autistic brains out of synch? Science 308, 1856–1858 10.1126/science.308.5730.1856 15976282

[B388] Wiener N. (1956). “The theory of prediction,” in Modern Mathematics for Engineers, ed. Beckenbach E. F. (New York, NY: McGraw Hill), 165–190.

[B389] Williams B. R. Lazic S. E. Ogilvie R. D. (2008). Polysomnographic and quantitative EEG analysis of subjects with long-term insomnia complaints associated with mild traumatic brain injury. Clin. Neurophysiol. 119, 429–438. 10.1016/j.clinph.2007.11.003 18083618

[B390] Winterer G. Ziller M. Dorn H. Frick K. Mulert C. Wuebben Y. (2000). Schizophrenia: reduced signal-to-noise ratio and impaired phase-locking during information processing. Clin. Neurophysiol. 111, 837–849. 10.1016/S1388-2457(99)00322-3 10802455

[B391] Wu H. T. Liu C. C. Lo M. T. Hsu P. C. Liu A. B. Chang K. Y. (2013). Multiscale cross-approximate entropy analysis as a measure of complexity among the aged and diabetic. Comput. Math. Methods Med. 2013, 324325. 10.1155/2013/324325 23864905 PMC3705813

[B392] Xu J. Liu Z.-R. Liu R. (1994). The measure of sequence complexity for EEG studies. Chaos Solitons Fractals 4, 2111–2119. 10.1016/0960-0779(94)90125-29370086

[B393] Yuan Q. Zhou W. Li S. Cai D. (2011). Epileptic EEG classification based on extreme learning machine and nonlinear features. Epilepsy Res. 96, 29–38. 10.1016/j.eplepsyres.2011.04.013 21616643

[B394] Yun K. Park H. K. Kwon D. H. Kim Y. T. Cho S. N. Cho H. J. (2012). Decreased cortical complexity in methamphetamine abusers. Psychiatry Res. 201, 226–232. 10.1016/j.pscychresns.2011.07.009 22445216

[B395] Zawski v. Giggs. Commonwealth of Massachusetts, Superior Court Department C.A. No 08-2280.

[B396] Zhou Y. X. Dougherty J. H. Hubner K. F. Bai B. Cannon R. L. Hutson R. K. (2008). Abnormal connectivity in the posterior cingulate and hippocampus in early Alzheimer’s disease and mild cognitive impairment. Alzheimers Dement. 4, 265–270. 10.1016/j.jalz.2008.04.006 18631977

[B397] Zou G. Y. (2012). Sample size formulas for estimating intraclass correlation coefficients with precision and assurance. Stat. Med. 31, 3972–3981. 10.1002/sim.5466 22764084

[B398] Zourdakis G. Patidar U. Pollonini L. Situ N. Rezaie R. Castillo E. M. (2011). “Default brain connectivity network in mild traumatic brain injury – preliminary MEG results,” in 2011 1st Middle East Conference on Biomedical Engineering (MECBME) Sharjah.

