# Supplementary material for: Traumatic Brain Injury Detection Using Electrophysiological Methods
Source: Front Hum Neurosci. 2015 Feb 4;9:11. doi: 10.3389/fnhum.2015.00011 (PMC4316720; doi:10.3389/fnhum.2015.00011)
Supplement: Supplementary file 1 [file Data_Sheet_1.PDF]

IX. Appendices

Appendix A: Literature Search Keywords and Results

Initial Keywords	# of hits (PubMed)	# of hits (DTIC)
quantitative electroencephalography	3061	397
quantitative electroencephalography traumatic brain	50	122
quantitative electroencephalography injury	153	231
quantitative electroencephalography concussion	15	44
quantitative electroencephalography diagnosis brain injury	132	156

Note: Greyed box indicates search return was fully explored by research team

Appendix B: Index of Studies Used to Assess Efficacy of qEEG Measures as mTBI Detection Tools

PubMed or DTIC ID	Authors	Year	Title	Journal Vol, Pages	Brief Description	Type of qEEG
22107157	Barr WB, Prichep LS, Chabot R, Powell MR, McCrea M	2012	Measuring brain electrical activity to track recovery from sport-related concussion	Brain Inj. 26(1), 58-66	Study with 59 concussed athletes and 31 controls; qEEG at injury, 8 and 45 days post-injury	qEEG
17113930	Bonanni E, Borghetti D, Fabbrini M, Maestri M, Cignoni F, Sartucci F, Murri L	2006	Quantitative EEG analysis in post-traumatic anosmia	Brain Res Bull. 71(1-3), 69-75	25 patient study; all have anosmia caused by head trauma; evaluated using frequency band power and topographical mapping	stimulus-dependent (evoked)
17704845	Chen XP, Tao LY, Chen AC	2006	Electroencephalogram and evoked potential parameters examined in Chinese mild head injury patients for forensic medicine	Neurosci Bull. 22(3),165-70	Study of 60 concussed patients and 30 healthy controls	qEEG and stimulus-dependent qEEG
14652089	Coutin-Churchman P, Añez Y, Uzcátegui M, Alvarez L, Vergara F, Mendez L, Fleitas R	2003	Quantitative spectral analysis of EEG in psychiatry revisited: drawing signs out of numbers in a clinical setting	Clin Neurophysiol. 114(12), 2294-306	Large study with 340 patients and 67 normal subjects	qEEG
18850345	Elting JW, Maurits N, van Weerden T, Spikman J, De Keyser J, van der Naalt J	2008	P300 analysis techniques in cognitive impairment after brain injury: comparison with neuropsychological and imaging data	Brain Inj. 22(11), 870-81	33 patient study with 21 healthy controls; looking at P300 recording from ERP; note the full text is from a dissertation (draft of final manuscript); uses ROC statistics	stimulus-dependent (ERP)
2579798	Gasser T, Bächer P, Steinberg H	1985	Test-retest reliability of spectral parameters of the EEG	Electroencephalogr Clin Neurophysiol. 60(4), 312-9	This paper studies test-retest reliability of the EEG technology, but, looks at standard EEG	standard EEG
23199430	Gosselin N, Bottari C, Chen JK, Huntgeburth SC, De Beaumont L, Petrides M, Cheung B, Ptito A	2012	Evaluating the cognitive consequences of mild traumatic brain injury and concussion by using electrophysiology	Neurosurg Focus 33(6),E7: 1-7	44 patient study looking at ERP technology	stimulus-dependent (ERP)
21309680	Gosselin N, Bottari C, Chen JK, Petrides M, Tinawi S, de Guise E, Ptito A	2011	Electrophysiology and functional MRI in post-acute mild traumatic brain injury	J Neurotrauma 28(3), 329-41	14 mTBI patients compared to 23 control subjects; study uses fMRI and ERPs	stimulus-dependent (ERP)
18226956	Gosselin N, Lassonde M, Petit D, Leclerc S, Mongrain V, Collie A, Montplaisir J	2009	Sleep following sport-related concussions	Sleep Med. 10(1),35-46	Small study (10 patients 11 controls) focused on sleep patterns, but they also look at qEEG power bands	qEEG

PubMed or DTIC ID	Authors	Year	Title	Journal Vol, Pages	Brief Description	Type of qEEG
17765604	Gudmundsson S, Runarsson TP, Sigurdsson S, Eiriksdottir G, Johnsen K	2007	Reliability of quantitative EEG features	Clin Neurophysiol. 118(10), 2162-71	Evaluates test-retest reliability in 15 elderly healthy adults	qEEG
2127153	Haglund Y, Persson HE	1990	Does Swedish amateur boxing lead to chronic brain damage? 3. A retrospective clinical neurophysiological study.	Acta Neurol Scand. 82(6), 353-60	Exploratory study comparing 47 boxers (high and low match count) to 25 soccer players and 25 track and field athletes; evoked potentials showed no changes but standard EEG had a few minor changes	standard and stimulus-dependent (evoked) qEEG
10574286	Kondacs A, Szabó M	1999	Long-term intra-individual variability of the background EEG in normals	Clin Neurophysiol. 110(10), 1708-16	This paper assesses test-retest variability of the qEEG method; uses healthy participants only	qEEG
15689708	Korn A, Golan H, Melamed I, Pascual-Marqui R, Friedman A	2005	Focal cortical dysfunction and blood-brain barrier disruption in patients with Postconcussion syndrome	J Clin Neurophysiol. 22(1),1-9	17 patient study; used matched controls; correlated site of EEG abnormality with blood brain barrier lesion using SPECT	qEEG
18183510	Leon-Carrion J, Martin-Rodriguez JF, Damas-Lopez J, Martin JM, Dominguez-Morales Mdel R	2008	A QEEG index of level of functional dependence for people sustaining acquired brain injury: the Seville Independence Index (SINDI).	Brain Inj. 22(1), 61-74	This study uses 40 or so TBI patients and focuses on testing a discriminant function for qEEG data; classifies patients based on functional status rather than categorical diagnosis	qEEG
20873308	Ljesević B, Martinović Z, Popović M, Jović S	2010	Visual vs. quantitative electroencephalographic analysis in patients with and without posttraumatic epilepsy	Med Pregl. 63(1-2),40-6	This study compares standard EEG and qEEG. But, the study population is post traumatic epileptic patients; study is in Serbian and needs to be translated; this type of journal has questionable quality	standard and qEEG
7932452	McClelland RJ, Fenton GW, Rutherford W	1994	The postconcussional syndrome revisited	J R Soc Med. 87(9), 508-10	The evidence from two recent prospective studies (Montgomery 1991 and Fenton 1993) is reviewed to shed further light on factors of post-concussive syndrome; qEEG spectra were examined in both studies within 24 hrs injury and again at 6 weeks post-injury	qEEG

PubMed or DTIC ID	Authors	Year	Title	Journal Vol, Pages	Brief Description	Type of qEEG
20611046	McCrea M, Prichep L, Powell MR, Chabot R, Barr WB	2010	Acute effects and recovery after sport-related concussion: a neurocognitive and quantitative brain electrical activity study	J Head Trauma Rehabil. 25(4), 283-92	28 person study with 28 matched controls; uses high-school and college athletes	qEEG
22159059	Moeller JJ, Tu B, Bazil CW	2011	Quantitative and qualitative analysis of ambulatory electroencephalography during mild traumatic brain injury	Arch Neurol. 68(12),1595-8	Case study only	qEEG
1876643	Montgomery EA, Fenton GW, McClelland RJ, MacFlynn G, Rutherford WH	1991	The psychobiology of minor head injury	Psychol Med. 21(2), 375-84	26 patient study with qEEG as one component to an evaluation panel; patients evaluated at admission, 6 weeks, and 6 months post-injury; exploratory research (part of the Belfast study series)	qEEG
17892969	Näpflin M, Wildi M, Sarnthein J	2007	Test-retest reliability of resting EEG spectra validates a statistical signature of persons	Clin Neurophysiol. 118(11),2519-24	55 healthy adults tested with eyes closed for test-retest reliability	qEEG
18817882	Näpflin M, Wildi M, Sarnthein J	2008	Test-retest reliability of EEG spectra during a working memory task	Neuroimage 43(4), 687-93	Test-retest for ERP; 41 healthy adults	stimulus-dependent (ERP)
23359586	O'Neil B, Prichep LS, Naunheim R, Chabot R	2012	Quantitative brain electrical activity in the initial screening of mild traumatic brain injuries	West J Emerg Med. 13(5),394-400	119 patient study using handheld qEEG device (BrainScope device)	qEEG
22855231	Prichep LS, Jacquin A, Filipenko J, Dastidar SG, Zabele S, Vodencarević A, Rothman NS	2012	Classification of traumatic brain injury severity using informed data reduction in a series of binary classifier algorithms.	IEEE Trans Neural Syst Rehabil Eng. 20(6),806-22	May be good example for combination of smooth pursuit eye tracking and qEEG; explores algorithm development for qEEG technology	qEEG
a574126 (DTIC ID)	Ptito A, Cheung B, Chen JK, Gosselin S, Huntgeburth S, Leonard G, Petrides M.	2011	Investigation of the correlation between neurocognitive function with advanced magnetic resonance imaging (MRI), electroencephalography (EEG) in patients with traumatic brain injury exposure	Contract Report DRDC Toronto CR 2011-015	This study looks at about 10-15 patients with each carefully matched to a normal control; EEG analysis is conventional and linked to a visual working memory task; qualitative type analysis only	qEEG
22361265	Slobounov S, Sebastianelli W, Hallett M	2012	Residual brain dysfunction observed one year post-mild traumatic brain injury: combined EEG and balance study.	Clin Neurophysiol. 123(9),1755-6	Larger study (49 concussed); measures qEEG but also balance	qEEG

PubMed or DTIC ID	Authors	Year	Title	Journal Vol, Pages	Brief Description	Type of qEEG
20851190	Sponheim SR, McGuire KA, Kang SS, Davenport ND, Aviyente S, Bernat EM, Lim KO	2011	Evidence of disrupted functional connectivity in the brain after combat-related blast injury	Neuroimage 54 Suppl 1,S21-9	9 soldiers with mTBI compared to 8 control subjects; uses EEG phase synchronization measurement	qEEG
2456196	Tebano MT, Cameroni M, Gallozzi G, Loizzo A, Palazzino G, Pezzini G, Ricci GF	1988	EEG spectral analysis after minor head injury in man	Electroencephalogr Clin Neurophysiol. 70(2),185-9	18 patient study; early analysis of alpha and beta frequency shifts	qEEG
11514257	Thatcher RW, Biver C, Gomez JF, North D, Curtin R, Walker RA, Salazar A	2001	Estimation of the EEG power spectrum using MRI T(2) relaxation time in traumatic brain injury	Clin Neurophysiol. 112(9),1729-45	18 mild to severe patients studied with qEEG and qMRI; statistical issues have been raised about this study (unacknowledged and unaccounted-for between-group age differences (see Arciniegas 2011)	qEEG
2035948	Thatcher RW, Cantor DS, McAlaster R, Geisler F, Krause P	1991	Comprehensive predictions of outcome in closed head-injured patients. The development of prognostic equations	Ann N Y Acad Sci. 620, 82-101	161 patient study of multiple diagnostic technologies including qEEG	qEEG
11207333	Thatcher RW, North DM, Curtin RT, Walker RA, Biver CJ, Gomez JF, Salazar AM	2001	An EEG severity index of traumatic brain injury	J Neuropsychiatry Clin Neurosci. 13(1), 77-87	Severity index discriminant function analysis; 40 patients with mTBI, 25 moderate, and 43 severe (15 days to 4 years post-injury). Cross validated in 503 VA patients	qEEG
2473888	Thatcher RW, Walker RA, Gerson I, Geisler FH	1989	EEG discriminant analyses of mild head trauma	Electroencephalogr Clin Neurophysiol. 73(2), 94-106	Large study with 608 mTBI patients and 108 age-matched normal subjects; study explores development of a discriminant function; field has been contentious with this study	qEEG
10462146	Thornton KE	1999	Exploratory investigation into mild brain injury and discriminant analysis with high frequency bands (32-64 Hz)	Brain Inj. 13(7),477-88	91 patient study (32 mTBI and 52 normals); evaluates the Thatcher discriminant and their own high-frequency discriminant; author's own discriminant evaluation was considered exploratory	qEEG
14591452	Thornton KE	2003	The electrophysiological effects of a brain injury on auditory memory functioning. The QEEG correlates of impaired memory	Arch Clin Neuropsychol. 18(4), 363-78	Study with 85 mTBI patients and 56 normal subjects (17 days to 27 years post-injury); evaluates another discriminant function; also failed to corroborate discriminant from Thatcher 1989 paper	qEEG and stimulus-dependent qEEG

PubMed or DTIC ID	Authors	Year	Title	Journal Vol, Pages	Brief Description	Type of qEEG
21436875	Tomkins O, Feintuch A, Benifla M, Cohen A, Friedman A, Shelef I	2011	Blood-brain barrier breakdown following traumatic brain injury: a possible role in posttraumatic epilepsy	Cardiovasc Psychiatry Neurol. 2011, 765923	37 patient study with mostly ancillary investigations; some direct comparison between experimental and control group qEEG data	qEEG
9706538	Trudeau DL, Anderson J, Hansen LM, Shagalov DN, Schmoller J, Nugent S, Barton S	1998	Findings of mild traumatic brain injury in combat veterans with PTSD and a history of blast concussion	J Neuropsychiatry Clin Neurosci. 10(3), 308-13	Tested the Thatcher discriminant function in 43 veteran patients; blast injury patients were easiest to discriminate	qEEG
17622982	Van Albada SJ, Rennie CJ, Robinson PA	2007	Variability of model-free and model-based quantitative measures of EEG	J Integr Neurosci. 6(2), 279-307	Test-retest paper using normal subjects only; reports positive demonstration of test-retest for qEEG	qEEG
9606435	von Bierbrauer A, Weissenborn K	1998	P300 after minor head injury (a follow-up examination).	Acta Neurol Belg. 98(1), 21-6	15 patient study at 24 hours, 1, 3, 8 weeks after mTBI; looks at the P300 of ERP; results point to limited value and predictive capacity	stimulus-dependent (ERP)
1425393	von Bierbrauer A, Weissenborn K, Hinrichs H, Scholz M, Künkel H	1992	[Automatic (computer-assisted) EEG analysis in comparison with visual EEG analysis in patients following minor cranio-cerebral trauma (a follow-up study)].	EEG EMG Z Elektroenzephalogr Elektromyogr Verwandte Geb. 23(3),151-7	31 patient study suggests qEEG as good alternative to standard EEG; evaluates a discriminant function; uses jack-knife evaluation; note article is in German; see Newer 2005 review for extracted figures and explanation of results	standard and qEEG
8829722	Watson MR, Fenton GW, McClelland RJ, Lumsden J, Headley M, Rutherford WH	1995	The post-concussional state: neurophysiological aspects	Br J Psychiatry. 167(4), 514-21	26 patient study measured at admission, 10 days and 6 weeks later; no normative sample population; exploratory	qEEG and stimulus-dependent qEEG
18083618	Williams BR, Lazic SE, Ogilvie RD	2008	Polysomnographic and quantitative EEG analysis of subjects with long-term insomnia complaints associated with mild traumatic brain injury	Clin Neurophysiol. 119(2), 429-38	Study looked at 9 mTBI patients and 9 controls	qEEG
